# Adenosine and 1,N^6^-Ethenoadenosine-Derived Nucleolipids: Synthesis, Lipophilicity (log*P*), and Cytotoxic Activity Compared to Conventional Cytostatics in Glioma and Glioblastoma Cell Lines †

**DOI:** 10.3390/ijms27114922

**Published:** 2026-05-29

**Authors:** Mona Lünswilken, Eugenia Bender-Arnst, Fatima Barakat, Eugen Leinweber, Uwe Beginn, Gabriel A. Bonaterra, Ralf Kinscherf

**Affiliations:** 1 Department of Medical Cell Biology, Institute for Anatomy and Cell Biology, Philipps-University of Marburg, Robert-Koch-Str. 8, D-35032 Marburg, Germany; 2 Organic Chemistry I–Bioorganic Chemistry, Institute of Chemistry of New Materials, University of Osnabrück, Barbarastr. 7, D-49076 Osnabrück, Germanyubeginn@uni-osnabrueck.de (U.B.)

**Keywords:** nucleolipids, nucleoside analogues, glioma, glioblastoma, cytotoxicity, lipophilicity, structure–activity

## Abstract

A series of nucleolipid derivatives based on adenosine and 1,N^6^-ethenoadenosine was synthesized, and their cytotoxicity was evaluated in glioma and glioblastoma cell models. Twenty O-2′,3′-ketalized nucleolipid derivatives were prepared as symmetric and asymmetric adenosine analogs. The lipophilicity was determined using the octanol/water partition method, yielding log*P_OW_* values from −0.04 to 4.08. First, cytotoxicity was screened in rat/human glioma cell lines (BT4Ca/GOS-3) using a PrestoBlue™ viability assay, with 5-fluorouridine (5-FUrd) as the reference compound. Selected derivatives with the highest cytotoxicity were evaluated in human glioblastoma cell lines U87 and U251, and their efficacy was compared with the chemotherapeutic agent temozolomide (TMZ). PMA-differentiated human THP-1 macrophages were used to assess cytotoxic side effects in human immune-related cells. Several derivatives induced 90–100% cytotoxicity at 50 µM after 48 h, with cytotoxicity increasing with alkyl chain length and reaching a maximum for derivatives bearing medium-length chains (C_15_–C_17_). In contrast, shorter or longer chains caused reduced activity. Cytotoxicity was independent of symmetric or asymmetric ketal configuration, while 1,N^6^-ethenoadenosine ketal derivatives displayed higher activity than the corresponding adenosine ketals. These novel derivatives indicate that lipophilicity and alkyl chain length are responsible for the cytotoxic effect in glioma and glioblastoma, and that they are more effective than 5-FUrd and TMZ.

## 1. Introduction

Tumors of the central nervous system (CNS) represent one of the most aggressive and therapeutically challenging cancer types. Despite advances in oncology, brain tumors continue to be linked with poor prognosis and high mortality [1]. In pediatric oncology, CNS tumors make up about 27% of all cancers and are the second most common malignancy after leukemia [2,3,4]. Among intracranial tumors, gliomas are the most frequent primary malignant brain tumors and exhibit considerable molecular and biological heterogeneity [5,6]. Depending on their subtype, gliomas vary considerably in growth behavior and treatment response, complicating both, diagnosis and therapy.

Current treatment strategies for malignant brain tumors typically involve a combination of surgical resection, radiotherapy, and chemotherapy. However, complete surgical removal is often not feasible due to the infiltrative growth of glioma cells and the critical anatomical localization of tumors within the brain [7]. Consequently, chemotherapy remains an essential component of treatment. A major limitation of many anticancer agents is their limited ability to penetrate cellular membranes and their restricted transport across the blood–brain barrier (BBB), which leads to lower drug concentrations at the tumor site [8,9]. Therefore, improving physicochemical properties such as lipophilicity and membrane penetrability is a key strategy for the development of more effective brain tumor therapies.

Nucleoside analogs are an important class of antimetabolite drugs used in cancer chemotherapy. Among these, 5-fluorouridine (5-FUrd) exerts cytostatic and cytotoxic effects by interfering with RNA metabolism and nucleotide biosynthesis [10]. Structural modifications, particularly the lipophilization, can enhance the pharmacokinetic and biological properties of these nucleoside analogs by improving cellular uptake and their transport across the cell membrane [11,12,13,14].

Previous studies have shown that hydrophobization of pyrimidine nucleosides, such as uridine and 5-methyluridine, can significantly increase their in vitro cytotoxicity [15]. In particular, adding lipophilic substituents at the O-2′,3′-positions of the ribose, in the form of cyclic ketals, as well as attaching additional hydrophobic groups to the nucleobase, greatly influences both, physicochemical and biological properties [16,17]. Structure–activity relationship studies further suggest that the length and placement of lipophilic groups are crucial for determining cytotoxic potency and membrane permeability.

Besides canonical nucleosides, modified purine derivatives such as 1,N^6^-ethenoadenosine play an important role as nucleoside analogs in medicinal chemistry. The fluorescent nucleoside analog 1,N^6^-ethenoadenosine, was first introduced by Barrio et al. [18] and has since become a versatile platform for nucleoside modification and biological studies. Consequently, structural modifications of adenosine and ethenoadenosine present promising strategies for developing innovative nucleoside-based therapeutics [19,20,21,22].

In addition to therapeutic efficacy, the evaluation of potential side effects is an essential aspect of drug development. Macrophages play a central role in innate and adaptive immunity and are therefore frequently used as cellular models to assess cytotoxic effects on immune-related cells [23]. In this context, PMA-differentiated human THP-1 macrophages are a well-established in vitro model for studying immune responses and cytotoxic side effects [24].

Guided by previous studies, this work aimed at first to synthesize novel adenosine and 1,N^6^-ethenoadenosine nucleolipid derivatives bearing symmetric and asymmetric O-2′,3′-ketal substituents and second to test their cytotoxic activity in comparison with conventional cytostatics. The lipophilicity of these compounds was experimentally determined, and their cytotoxic activity was evaluated in human/rat glioma and glioblastoma cell lines (BT4Ca, GOS-3, U87, U251). Potential cytotoxic side effects were investigated by using PMA-differentiated THP-1 macrophages. Overall, this study provides new insights into the relationship between lipophilicity, alkyl chain length, and improved cytotoxic activity, thereby contributing to the development of nucleoside-based therapeutic strategies for brain tumors.

## 2. Results

### 2.1. Synthesis of Nucleolipid Derivatives

**Figure 1:**
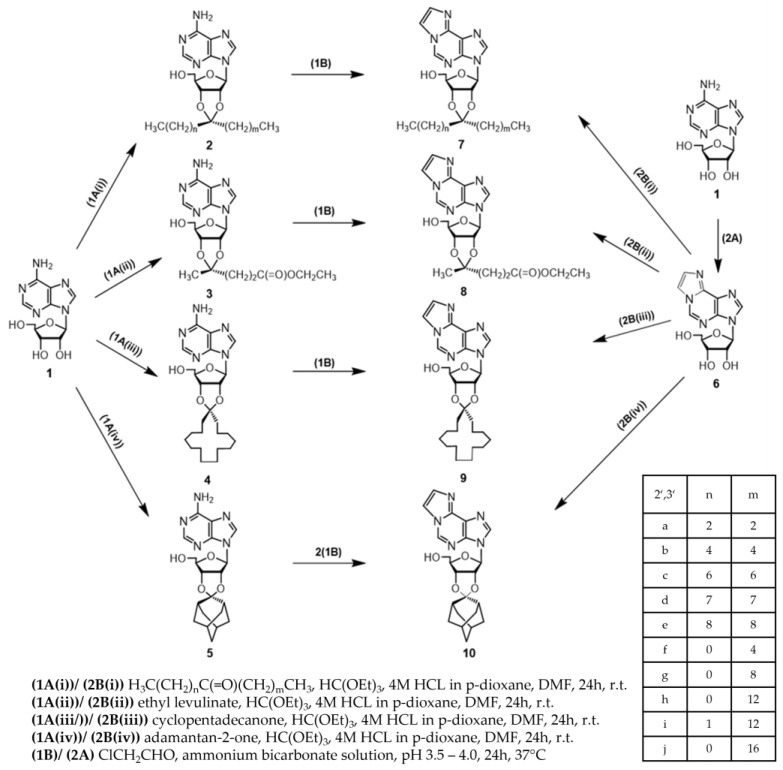
Synthetic pathways for the preparation of O-2′,3′-ketalized adenosine and 1,N^6^-ethenoadenosine nucleolipids. The scheme includes (A) ketalization of adenosine at the O-2′,3′-position and (B) ethenylation of adenosine and adenosine ketals at the 1,N^6^-position.

A series of nucleolipid derivatives based on adenosine and 1,N^6^-ethenoadenosine was synthesized via O-2′,3′-ketalization of the corresponding nucleosides (Figure 1). Adenosine served as the primary starting material, while 1,N^6^-ethenoadenosine was synthesized according to the procedure described by Barrio et al. [18].

In total, 26 nucleolipid derivatives were included in this study, comprising 21 newly synthesized compounds and a set of structurally related derivatives reported previously [13,14,15,17], to enable comprehensive structure–activity relationship analysis (Figure 1).

Ketalization at the O-2′,3′-position of the ribose moiety was carried out using lipophilic substituents with varying alkyl chain lengths in order to systematically modulate the lipophilicity of the nucleoside derivatives (Figure 1). Both symmetric and asymmetric ketal derivatives were synthesized, including compounds with the same alkyl chain lengths but different substitution patterns. The substituents covered a broad structural range, including linear alkyl chains (C_7_–C_19_), cyclic moieties such as pentanylidene groups, and bulky substituents, such as adamantylidene and ethyl levulinate residues (Figure 1 and Table 1).

The synthesized compounds were named according to a previously established nomenclature system for nucleolipids [14]. An overview of the compound numbering and corresponding NS/NL codes is provided in Table 1.

The corresponding 1,N^6^-ethenoadenosine nucleolipids were synthesized using two complementary synthetic approaches. In the first approach, preformed adenosine ketals were subjected to ethenylation with chloroacetaldehyde, yielding the corresponding ethenoadenosine derivatives. Alternatively, 1,N^6^-ethenoadenosine was synthesized first and then functionalized via ketalization at the O-2′,3′-position of the ribose moiety. While the first route provided access to a broader range of derivatives, the second approach allowed for a more direct synthesis of ethenoadenosine ketals.

Asymmetric ketal derivatives were obtained as diastereomeric mixtures that could not be separated under the regular chromatographic techniques. The structures of all synthesized compounds were confirmed by elemental analysis, electrospray ionization mass spectrometry (ESI-MS), ^1^H and ^13^C NMR spectroscopy, as well as UV/Vis and fluorescence spectroscopy for ethenoadenosine derivatives.

Detailed synthetic procedures and full analytical data for all compounds are provided in the Supplementary Materials. Symmetrical and asymmetrical nucleolipid derivatives are described in Sections S1 and S2, respectively, each including individual experimental procedures and compound-specific characterization data. Numbering schemes for all compound classes are shown in Supplementary Figures S1–S4.

### 2.2. Lipophilicity and Membrane Permeability

The lipophilicity of all nucleolipid derivatives was experimentally determined using octanol/water (log*P_OW_*) and cyclohexane/water (log*P_ChW_*) partition systems (Table 2). The obtained (log*P_OW_*) values ranged from −0.04 to 4.08, while (log*P_ChW_*) values varied between −2.98 and 2.48, indicating a broad spectrum of hydrophilic to highly lipophilic compounds.

To further characterize membrane-interaction properties, the difference between both partition systems (Δlog*P*) was evaluated. Δlog*P* values ranged from −0.83 to 3.92 and allowed estimation of hydrogen-bonding capacity and passive membrane permeability. Using the empirical relationship described by Young et al. [25], the calculated log(c_brain_/c_blood_) values ranged from −1.14 to 1.73, indicating pronounced differences in the predicted ability of the compounds to cross the blood–brain barrier.

**Figure 2:**
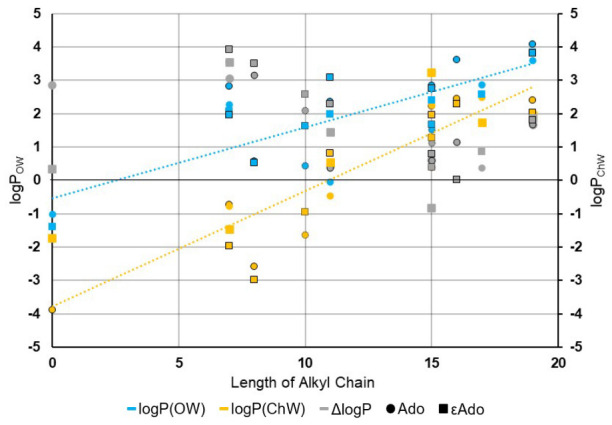
Comparison between alkyl chain length and log*P_OW_*, log*P_ChW_*, and Δlog*P* values of nucleolipid derivatives. Each data point represents an individual compound. Multiple values at identical alkyl chain lengths arise from structurally distinct derivatives (symmetric, asymmetric, and cyclic ketal derivatives) as well as from adenosine and 1,N^6^-ethenoadenosine analogs that share the same chain length but differ in substitution pattern and nucleobase structure. Circles represent adenosine derivatives (Ado), while squares represent 1,N^6^-ethenoadenosine derivatives (εAdo). Symmetric derivatives are shown without outlines, asymmetric derivatives are indicated by black outlines, and cyclic derivatives are represented by dashed outlines. The dotted lines represent linear trendlines for log*P_OW_* and log*P_ChW_*.

A clear relationship between lipophilicity and membrane permeability depending on alkyl chain length was observed (Figure 2/Table 2). Derivatives with short alkyl chains (C_7_) exhibited high Δlog*P* values (e.g., NL_5.4.0.0 (2a): 3.05; εNL_5.4.0.0 (7a): 3.54), indicating strong hydrogen-bonding interactions and decreased passive membrane permeability (Figure 2/Table 2). As the alkyl chain length increased (C_11_–C_17_), Δlog*P* values decreased significantly (e.g., NL_5.5.0.0 (2b): 0.42; NL_5.7.0.0 (2c): 0.60), accompanied by increasing log(c_brain_/c_blood_) values, suggesting enhanced permeability across biological membranes (Table 2).

The most favorable physicochemical properties were observed for derivatives with medium alkyl chain lengths (C_15_–C_17_). In particular, εNL_5.7.0.0 (7c) exhibited the lowest Δlog*P* value (−0.83), and the highest predicted BBB permeability (log(c_brain_/c_blood_) = 1.73) (Table 2). In contrast, further elongation of the alkyl chain (C_19_) resulted in increasing Δlog*P* values (e.g., NL_5.3.0.0 (2e): 1.66; εNL_5.3.0.0 (7e): 1.87), indicating reduced membrane permeability compared to medium-chain derivatives (Table 2).

Importantly, these trends were observed for both, symmetric and asymmetric derivatives, demonstrating that alkyl chain length, rather than substitution pattern, is the main factor influencing lipophilicity and membrane-interaction properties. Additionally, cyclic and bulky substituents showed distinct behavior.

Cyclized derivatives exhibited moderate Δlog*P* values, whereas bulky substituents such as adamantylidene groups were associated with increased Δlog*P* values and reduced permeability.

A clear dependence of lipophilicity and membrane permeability on alkyl chain length was observed (Figure 2). To facilitate visualization of the relationship between alkyl chain length and lipophilicity, linear regression trendlines were included for log*P_OW_* and log*P_ChW_* values (Figure 2). Both parameters show an overall increase with increasing alkyl chain length, indicating enhanced lipophilicity and reduced polarity of the derivatives. Derivatives with short alkyl chains (C_7_) exhibited high Δlog*P* values (e.g., NL_5.4.0.0 (2a): 3.05; εNL_5.4.0.0 (7a): 3.54), indicating strong hydrogen-bonding interactions and reduced passive membrane permeability. As the alkyl chain length increased from C_11_ to C_17_, Δlog*P* values decreased, accompanied by increasing log_(cbrain/cblood)_ values, suggesting enhanced permeability across biological membranes (Figure 2/Table 2). Notably, the combination of increasing log*P_OW_* and log*P_ChW_* values with Δlog*P* values approaching zero suggests a more favorable balance between lipophilicity and hydrogen-bonding capacity. This indicates improved partitioning into lipid membranes while maintaining sufficient polarity, which may facilitate passive membrane permeability, consistent with the observed optimal properties of medium-chain derivatives (C_15_–C_17_) (Figure 2 and Table 2).

Overall, these findings demonstrate that alkyl chain length and substituent structure are key determinants of lipophilicity and membrane penetrability. Medium-chain nucleolipid derivatives (C_15_–C_17_) exhibit the most favorable balance of physicochemical properties, suggesting an enhanced potential for cellular uptake and penetration of the blood–brain barrier.

### 2.3. Cytotoxic Activity of Nucleolipid Derivatives in Rat BT4Ca and Human GOS-3 Glioma Cell Lines

The cytotoxic activity of the synthesized nucleolipid derivatives was evaluated in rat BT4Ca and human GOS-3 glioma cells after 48 h of incubation using a PrestoBlue™ viability assay. Overall, the tested compounds showed clear structure-dependent effects on cell viability. The activity was strongly influenced by alkyl chain length, while the difference between symmetric and asymmetric ketal substitution was less pronounced. In general, short-chain derivatives and bulky substituents showed little or no cytotoxicity, whereas compounds with medium-length alkyl chains displayed the strongest effects. Additionally, several derivatives exhibited higher cytotoxicity than 5-FUrd (reference compound).

#### 2.3.1. Cytotoxic Effects on Rat BT4Ca Glioma Cells

**Figure 3:**
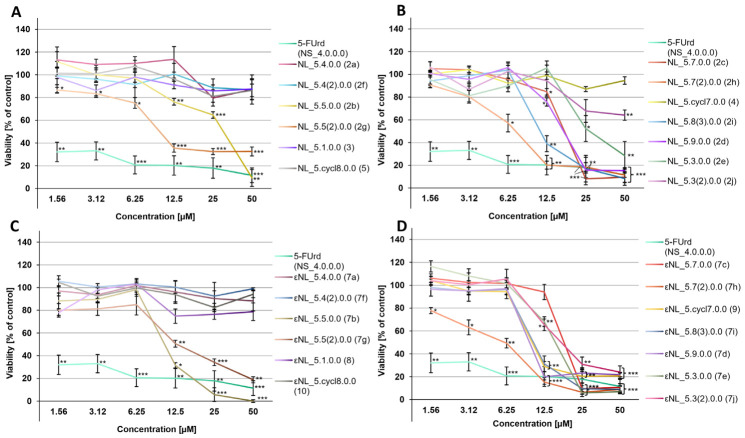
Viability (in % of control) of rat BT4Ca glioma cells after 48 h incubation with NS_4.0.0.0 (5-FUrd; reference control) or nucleolipid derivatives. (**A**) Short-chain and bulky adenosine derivatives: NL_5.4.0.0 (2a), NL_5.4_2_.0.0 (2f), NL_5.5.0.0 (2b), NL_5.5_2_.0.0 (2g), NL_5.1.0.0 (3), and NL_5.cycl8.0.0 (5). (**B**) Medium- and long-chain adenosine derivatives: NL_5.7.0.0 (2c), NL_5.7_2_.0.0 (2h), NL_5.cycl7.0.0 (4), NL_5.8_3_.0.0 (2i), NL_5.9.0.0 (2d), NL_5.3.0.0 (2e), and NL_5.3_2_.0.0 (2j). (**C**) Short-chain and bulky 1,N^6^-ethenoadenosine derivatives: εNL_5.4.0.0 (7a), εNL_5.4_2_.0.0 (7f), εNL_5.5.0.0 (7b), εNL_5.5_2_.0.0 (7g), εNL_5.1.0.0 (8), and εNL_5.cycl8.0.0 (10). (**D**) Medium- and long-chain 1,N^6^-ethenoadenosine derivatives: εNL_5.7.0.0 (7c), εNL_5.7_2_.0.0 (7h), εNL_5.cycl7.0.0 (9), εNL_5.8_3_.0.0 (7i), εNL_5.9.0.0 (7d), εNL_5.3.0.0 (7e), and εNL_5.3_2_.0.0 (7j). Values are given as mean ± SEM (in % viability of control [incubation with medium alone = 100% viability]); * *p* ≤ 0.05, ** *p* ≤ 0.01, *** *p* ≤ 0.001 (significance vs. negative control [equal concentration of DMSO to substance solution, 0.5%]); *n* = 4 independent experiments assayed in quadruplicate.

In rat BT4Ca cells, 5-FUrd induced pronounced significant cytotoxic effects across the entire concentration range, with reductions in viability of 67.9% at 1.56 µM and 66.9% at 3.12 µM (both *p* ≤ 0.01), 79.3% at 6.25 µM (*p* ≤ 0.001), 79.8% at 12.5 µM (*p* ≤ 0.01), and 82.1% and 88.5% at 25 and 50 µM (both *p* ≤ 0.001), as depicted in Figure 3A–D. For improved comparability, all cytotoxicity data are additionally summarized in Supplementary Table S1.

Symmetrical NL_5.4.0.0 (2a) did not exhibit relevant effects at low concentrations but instead increased viability by 13.1%, 9.0%, 10.1%, and 13.6% at 1.56–12.5 µM (Figure 3A). At higher concentrations, a moderate cytotoxicity of 20.3% and 12.9% was observed (Figure 3A). Similarly, asymmetrical NL_5.4_2_.0.0 (2f) showed only weak effects, with reductions in viability of 0.9%, 3.8%, and 8.5% at 12.5 µM, followed by reductions of 11.3% and 13.2% at higher concentrations (Figure 3A).

Symmetrical NL_5.5.0.0 (2b) showed no cytotoxicity at low concentrations but induced a significant reduction in viability of 23.8% at 12.5 µM (*p* ≤ 0.01), which increased to 35.3% at 25 µM (*p* ≤ 0.001) and 90.7% at 50 µM (*p* ≤ 0.01) (Figure 3A). Asymmetrical NL_5.5_2_.0.0 (2g) exhibited cytotoxic effects already at low concentrations, with reductions in viability of 13.4% and 16.5%, and significantly reduced viability by 24.7% at 6.25 µM (*p* ≤ 0.05), followed by pronounced significant (*p* ≤ 0.001) cytotoxicity of 64.4%, 67.5%, and 67.4% at higher concentrations (Figure 3A).

NL_5.1.0.0 (3) showed only moderate effects, with reduction ranging from 2.1% to 13.7% at lower concentrations and reaching 9.0%, 14.2%, and 12.5% at 12.5, 25, and 50 µM, respectively (Figure 3A). NL_5.cycl8.0.0 (5) exhibited only weak effects, with reductions in viability of 1.5% to 7.5% at low concentrations and 18.8% and 13.6% at higher concentrations (Figure 3A).

NL_5.7.0.0 (2c) showed no significant cytotoxicity up to 6.25 µM, but induced a 15.3% reduction in viability at 12.5 µM (*p* ≤ 0.05) (Figure 3B). At 25 µM and 50 µM, cytotoxicity increased sharply to 92.0% and 90.5% (both *p* ≤ 0.001), exceeding that of 5-FUrd by 9.9% and 2.0%, respectively (Figure 3B). Asymmetrical NL_5.7_2_.0.0 (2h) showed cytotoxic effects of 9.2% and 19.7% at low concentrations and significantly reduced viability by 42.8% at 6.25 µM (*p* ≤ 0.05) and 80.0% at 12.5 µM (*p* ≤ 0.01), followed by strong cytotoxicity of 81.6% and 88.7% at higher concentrations (*p* ≤ 0.001), reaching levels comparable to 5-FUrd (Figure 3B).

Cyclic NL_5.cycl7.0.0 (4) exhibited only marginal effects, not exceeding 7.7% at intermediate concentrations and 13.0% at 25 µM. In contrast, NL_5.8_3_.0.0 (2i) induced significant cytotoxicity of 60.9% at 12.5 µM (*p* ≤ 0.05), 83.0% at 25 µM (*p* ≤ 0.01), and 91.6% at 50 µM (*p* ≤ 0.001), reaching toxicity comparable to 5-FUrd (Figure 3B).

NL_5.9.0.0 (2d) showed minimal effects at low concentrations but significantly reduced viability by 24.2% at 12.5 µM (*p* ≤ 0.05) and by 84.2% and 84.8% at 25 and 50 µM (both *p* ≤ 0.001), exceeding 5-FUrd by 2.2% at 25 µM (Figure 3B). NL_5.3.0.0 (2e) showed variable effects at lower concentrations but significantly reduced viability by 71.5% at 50 µM (*p* ≤ 0.001) (Figure 3B). Asymmetrical NL_5.3_2_.0.0 (2j) induced moderate cytotoxicity, reaching a significant reduction of 35.9% at 50 µM (*p* ≤ 0.01) (Figure 3B).

Symmetrical 1,N^6^-ethenoadenosine derivative εNL_5.4.0.0 (7a) and asymmetrical εNL_5.4_2_.0.0 (7f) exhibited only minimal cytotoxicity across all concentrations, with reductions not exceeding 11.7% and 7.5%, respectively (Figure 3C). Similarly, εNL_5.1.0.0 (8) showed moderate cytotoxicity ranging from 2.0% to 25.2%, while εNL_5.cycl8.0.0 (10) induced only minor reductions of up to 17.5% (Figure 3C).

In contrast, εNL_5.5.0.0 (7b) displayed pronounced activity, reducing viability by 68.5% at 12.5 µM (*p* ≤ 0.05) and further to 94.2% and 97.7% at 25 and 50 µM (both *p* ≤ 0.001) (Figure 3C). At these higher concentrations, the effect clearly exceeded that of 5-FUrd by 12.2% and 13.8%, respectively (Figure 3C). A comparable trend was observed for εNL_5.5_2_.0.0 (7g), which reduced viability by 49.4% at 12.5 µM (*p* ≤ 0.01), followed by 65.7% and 81.2% at 25 and 50 µM (both *p* ≤ 0.001), thereby surpassing 5-FUrd by 18.8% at the highest concentration (Figure 3C).

Pentadecanyl derivatives exhibited the strongest effects. εNL_5.7.0.0 (7c) reduced viability by 90.4% at 25 µM and 89.2% at 50 µM (both *p* ≤ 0.001), exceeding 5-FUrd by 8.4% at 25 µM (Figure 3D). εNL_5.7_2_.0.0 (7h) already showed significant cytotoxicity at low concentrations, with reductions of 22.1% at 1.56 µM and 36.9% at 3.12 µM (both *p* ≤ 0.05), while 50.7% cytotoxicity at 6.25 µM was significant at the *p* ≤ 0.01 level (Figure 3D). At higher concentrations, cytotoxicity increased further to 84.6% at 12.5 µM (*p* ≤ 0.001), and to 93.6% and 91.0% at 25 and 50 µM, each reaching statistical significance at *p* ≤ 0.01, exceeding the effect of 5-FUrd by up to 11.6% (Figure 3D).

The cyclic derivative εNL_5.cycl7.0.0 (9) significantly reduced viability by 71.1% at 12.5 µM and 79.7% at 25 µM (both *p* ≤ 0.01), whereas the reduction of 79.2% at 50 µM was even more pronounced (*p* ≤ 0.001) (Figure 3D). At 25 µM, its activity was comparable to 5-FUrd (Figure 3D). Likewise, εNL_5.8_3_.0.0 (7i) induced strong cytotoxicity, reducing viability by 90.5% and 91.1% at 25 and 50 µM, respectively (both *p* ≤ 0.001), exceeding the reference compound by up to 8.4% (Figure 3D).

εNL_5.9.0.0 (7d) caused a marked reduction in viability of 76.6% at 12.5 µM (*p* ≤ 0.001), followed by 76.9% at 25 µM, which remained statistically significant at *p* ≤ 0.01, and 78.3% at 50 µM (*p* ≤ 0.001) (Figure 3D). Similarly, εNL_5.3.0.0 (7e) showed strong effects at higher concentrations, reducing viability by 94.1% at 25 µM and 93.2% at 50 µM (both *p* ≤ 0.001), thereby exceeding 5-FUrd by 12.1% and 4.7%, respectively (Figure 3D). Finally, εNL_5.3_2_.0.0 (7j) significantly reduced viability from 12.5 µM onward, with reductions of 34.6% (*p* ≤ 0.05), 69.2% (*p* ≤ 0.01), and 76.0% (*p* ≤ 0.001) at increasing concentrations (Figure 3D).

#### 2.3.2. Cytotoxic Effects on Human GOS-3 Glioma Cells

**Figure 4:**
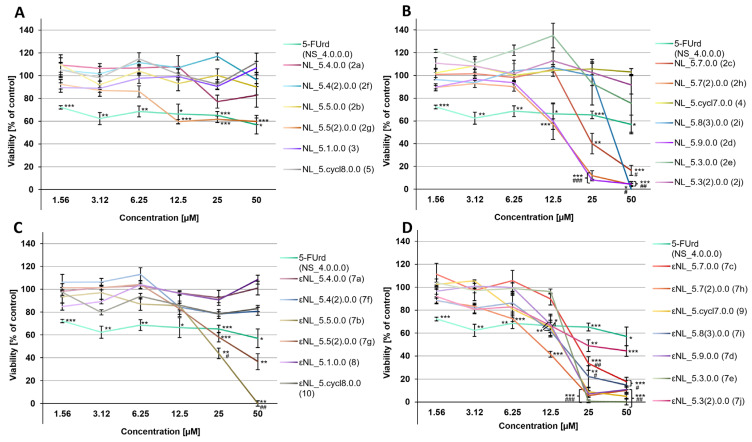
Viability (in % of control) of human GOS-3 glioma cells after 48 h incubation with NS_4.0.0.0 (5-FUrd; reference control) or nucleolipid derivatives. (**A**) Short-chain and bulky adenosine derivatives: NL_5.4.0.0 (2a), NL_5.4_2_.0.0 (2f), NL_5.5.0.0 (2b), NL_5.5_2_.0.0 (2g), NL_5.1.0.0 (3), and NL_5.cycl8.0.0 (5). (**B**) Medium- and long-chain adenosine derivatives: NL_5.7.0.0 (2c), NL_5.7_2_.0.0 (2h), NL_5.cycl7.0.0 (4), NL_5.8_3_.0.0 (2i), NL_5.9.0.0 (2d), NL_5.3.0.0 (2e), and NL_5.3_2_.0.0 (2j). (**C**) Short-chain and bulky 1,N^6^-ethenoadenosine derivatives: εNL_5.4.0.0 (7a), εNL_5.4_2_.0.0 (7f), εNL_5.5.0.0 (7b), εNL_5.5_2_.0.0 (7g), εNL_5.1.0.0 (8), and εNL_5.cycl8.0.0 (10). (**D**) Medium- and long-chain 1,N^6^-ethenoadenosine derivatives: εNL_5.7.0.0 (7c), εNL_5.7_2_.0.0 (7h), εNL_5.cycl7.0.0 (9), εNL_5.8_3_.0.0 (7i), εNL_5.9.0.0 (7d), εNL_5.3.0.0 (7e), and εNL_5.3_2_.0.0 (7j). Values are given as mean ± SEM (in % viability of control [incubation with medium alone = 100% viability]); * *p* ≤ 0.05, ** *p* ≤ 0.01, *** *p* ≤ 0.001 (significance vs. negative control [equal concentration of DMSO to substance solution, 0.5%]); ^#^ *p* ≤ 0.05, ^##^ *p* ≤ 0.01, ^###^ *p* ≤ 0.001 (significance vs. 5-FUrd [equal concentration to substance solution]); *n* = 4 independent experiments assayed in quadruplicate.

In human GOS-3 glioma cells, 5-FUrd (NS_4.0.0.0) already induced significant cytotoxicity across the entire concentration range after 48 h. Viability was reduced by 27.8% at 1.56 µM (*p* ≤ 0.001 vs. DMSO), by 37.6% at 3.12 µM (*p* ≤ 0.01), by 31.5% at 6.25 µM (*p* ≤ 0.01), by 33.6% at 12.5 µM (*p* ≤ 0.05), by 34.8% at 25 µM (*p* ≤ 0.001), and by 43.0% at 50 µM (*p* ≤ 0.05). These reference data are shown throughout Figure 4A–D. For improved comparability, all cytotoxicity data are additionally summarized in Supplementary Table S1.

Among the short-chain and bulky adenosine derivatives, symmetrical NL_5.4.0.0 (2a) did not exert relevant effects at lower concentrations but rather increased viability by 9.3%, 6.4%, 6.8%, and 7.9% at 1.56, 3.12, 6.25, and 12.5 µM, respectively (Figure 4A). At higher concentrations, viability was reduced by 22.8% at 25 µM and by 17.3% at 50 µM (Figure 4A). Likewise, asymmetrical NL_5.4_2_.0.0 (2f) showed no cytotoxicity, with viability increased by 4.9%, 1.6%, 11.5%, 6.9%, and 16.9% at 1.56, 3.12, 6.25, 12.5, and 25 µM, respectively, while only a minor reduction of 3.6% was observed at 50 µM (Figure 4A). Symmetrical NL_5.5.0.0 (2b) similarly showed no pronounced effects, with viability increased by 9.4% at 1.56 µM and 3.9% at 6.25 µM, while reductions of 8.0%, 6.9%, and 9.9% were observed at 3.12, 12.5, and 50 µM; at 25 µM, viability remained essentially unchanged, with a marginal increase of 0.3% (Figure 4A). NL_5.1.0.0 (3) also remained weakly active, with reductions of 10.5% and 11.2% at 1.56 and 3.12 µM, followed by only minor effects at 6.25 µM and 12.5 µM, where viability was reduced by 2.3% and 0.8%, respectively; at 25 µM, viability was still reduced by only 9.4%, whereas at 50 µM it was increased by 6.7% (Figure 4A). Similarly, NL_5.cycl8.0.0 (5) did not induce relevant cytotoxicity, with viability increased by 5.1%, 14.8%, 1.2%, and 11.4% at 1.56, 6.25, 12.5, and 50 µM, respectively, and reduced only marginally by 1.9% at 3.12 µM and by 7.6% at 25 µM (Figure 4A).

In contrast, asymmetrical NL_5.5_2_.0.0 (2g) displayed pronounced activity at higher concentrations. Viability was reduced by 7.5%, 13.0%, and 13.8% already at 1.56, 3.12, and 6.25 µM, respectively, and dropped significantly by 40.3% at 12.5 µM (*p* ≤ 0.001 vs. DMSO), making this compound 6.6% more cytotoxic than 5-FUrd at the same concentration (Figure 4A). At 25 and 50 µM, viability was reduced by 38.4% and 40.1%, respectively, in both cases with *p* ≤ 0.001 versus DMSO; at these concentrations, the effect was comparable to that of 5-FUrd (Figure 4A).

Among the medium- and long-chain adenosine derivatives, the symmetrical NL_5.7.0.0 (2c) showed the first marked effects only at higher concentrations. Viability remained essentially unchanged at 1.56–12.5 µM, with values corresponding to an increase of 0.9% at 1.56 µM, 1.4% at 3.12 µM, a reduction of 1.8% at 6.25 µM, and an increase of 5.2% at 12.5 µM (Figure 4B). However, at 25 µM viability was reduced by 59.9% (*p* ≤ 0.01 vs. DMSO), and the compound was 25.2% more cytotoxic than 5-FUrd at the same concentration (Figure 4B). At 50 µM, viability was reduced by 83.4% (*p* ≤ 0.001 vs. DMSO), and the compound remained significantly more cytotoxic than 5-FUrd, exceeding its effect by 40.5% (Figure 4B). Asymmetrical NL_5.7_2_.0.0 (2h) showed moderate reductions of 10.5%, 7.2%, and 9.8% at 1.56, 3.12, and 6.25 µM, respectively, before viability dropped significantly by 42.8% at 12.5 µM (*p* ≤ 0.001 vs. DMSO); at this concentration, the compound was also significantly more cytotoxic than 5-FUrd, exceeding its effect by 9.2%. At 25 and 50 µM, viability was reduced by 88.1% and 95.9%, respectively, both with *p* ≤ 0.001 versus DMSO (Figure 4B). These effects were likewise significantly stronger than those of 5-FUrd, with *p* ≤ 0.001 at 25 µM and *p* ≤ 0.05 at 50 µM; cytotoxicity exceeded that of 5-FUrd by 53.3% and 52.9%, respectively (Figure 4B). By contrast, cyclic NL_5.cycl7.0.0 (4) remained inactive across the full concentration range under test, with viability increased by 2.2%, 8.6%, 4.4%, 5.6%, and 3.1% at 1.56, 3.12, 12.5, 25, and 50 µM, respectively, while no change was observed at 6.25 µM (Figure 4B).

NL_5.8_3_.0.0 (2i) exerted little effect at low and intermediate concentrations, with viability reduced by 3.7% and 6.3% at 1.56 and 3.12 µM, and increased by 4.0% and 6.8% at 6.25 and 12.5 µM; at 25 µM, viability was essentially unchanged, with a reduction of 0.3% (Figure 4B). However, at 50 µM viability dropped to 0%, corresponding to a cytotoxic effect of 100.0%, which was significant versus both DMSO and 5-FUrd (both *p* ≤ 0.05) (Figure 4B). At this concentration, the compound was 57.0% more cytotoxic than 5-FUrd. Symmetrical NL_5.9.0.0 (2d) showed modest effects at low concentrations, with reductions of 10.4%, 3.4%, and 6.3% at 1.56, 3.12, and 6.25 µM, respectively (Figure 4B). At 12.5 µM, viability was reduced by 40.2%, although this effect was not significant versus DMSO (Figure 4B). At 25 and 50 µM, however, viability was reduced by 92.0% and 95.4%, respectively, both with *p* ≤ 0.001 versus DMSO. These reductions were also significant versus 5-FUrd, with *p* ≤ 0.001 at 25 µM and *p* ≤ 0.01 at 50 µM; at 50 µM, cytotoxicity exceeded that of 5-FUrd by 52.5% (Figure 4B). Symmetrical nonadecanyl derivative NL_5.3.0.0 (2e) showed no cytotoxicity at low and intermediate concentrations but instead increased viability by 21.6%, 10.8%, 22.2%, and 35.1% at 1.56, 3.12, 6.25, and 12.5 µM, respectively (Figure 4B). At 25 µM, viability was still reduced by 7.0%, and although it declined further at 50 µM, corresponding to a reduction of 24.5%; however, this effect was insignificant versus DMSO (Figure 4B). Similarly, asymmetrical NL_5.3_2_.0.0 (2j) remained inactive, with viability increased by 10.6%, 8.1%, 1.5%, 12.8%, and 2.68% at 1.56, 3.12, 6.25, 12.5, and 25 µM, respectively, and reduced only by 8.4% at 50 µM (Figure 4B).

Among the short-chain and bulky 1,N^6^-ethenoadenosine derivatives, εNL_5.4.0.0 (7a) caused only minor effects, with reductions of 1.8%, 3.3%, and 7.5% at 1.56, 12.5, and 25 µM, respectively, while viability was increased by 1.5%, 3.0%, and 1.0% at 3.12, 6.25, and 50 µM (Figure 4C). Asymmetrical εNL_5.4_2_.0.0 (7f) increased viability by 6.1%, 6.4%, and 13.1% at 1.56, 3.12, and 6.25 µM, respectively, but reduced viability by 15.6%, 21.7%, and 19.3% at 12.5, 25, and 50 µM (Figure 4C). Symmetrical εNL_5.5.0.0 (7b) showed only modest effects up to 12.5 µM, with reductions of 6.5%, 3.0%, 13.0%, and 14.4% at 1.56, 3.12, 6.25, and 12.5 µM, respectively (Figure 4C). However, at 25 µM viability was reduced by 56.3%, which was significant versus DMSO (*p* ≤ 0.01) and 5-FUrd (*p* ≤ 0.05); interestingly, at this concentration, the compound was 21.5% more cytotoxic than 5-FUrd (Figure 4C). At 50 µM, viability dropped to 0.0%, corresponding to a cytotoxic effect of 100.0%, which was significant versus both, DMSO and 5-FUrd (both *p* ≤ 0.01); here, cytotoxicity exceeded that of 5-FUrd by 57.0% (Figure 4C). Asymmetrical εNL_5.5_2_.0.0 (7g) remained largely inactive at low concentrations, with viability increased by 1.4% at 1.56 µM, unchanged at 3.12 µM, and increased by 4.1% at 6.25 µM. At 12.5 µM, viability was reduced by 17.1%, and this decrease was significant versus DMSO (Figure 4C). Stronger effects occurred at 25 and 50 µM, where viability was reduced by 41.9% (*p* ≤ 0.001 vs. DMSO) and 63.4% (*p* ≤ 0.01 vs. DMSO), respectively; at these concentrations, the compound was 7.2% and 20.4% more cytotoxic than 5-FUrd (Figure 4C).

εNL_5.1.0.0 (8) showed only moderate effects, reducing viability by 15.1% and 10.8% at 1.56 and 3.12 µM, while viability increased by 3.9% at 6.25 µM, was reduced by 3.7% at 12.5 µM and by 9.3% at 25 µM, and increased again by 8.4% at 50 µM (Figure 4C). Likewise, εNL_5.cycl8.0.0 (10) remained weakly active, with reductions of 2.7%, 20.3%, 6.1%, 14.0%, 21.7%, and 16.9% across the concentration range from 1.56 to 50 µM (Figure 4C).

The medium- and long-chain ethenoadenosine derivatives were markedly more potent. Symmetrical εNL_5.7.0.0 (7c) increased viability by 11.3% at 1.56 µM, reduced it slightly by 2.8% at 3.12 µM, and again increased it by 5.6% at 6.25 µM (Figure 4D). At 12.5 µM, viability was reduced by 10.3%, whereas at 25 and 50 µM it dropped sharply by 66.5% and 82.2%, respectively (Figure 4D). Both high-dose effects were significant versus DMSO (both *p* ≤ 0.001) and also versus 5-FUrd, with *p* ≤ 0.001 at 25 µM and *p* ≤ 0.05 at 50 µM (Figure 4D). At these concentrations, the compound was 31.8% and 39.2% more cytotoxic than 5-FUrd (Figure 4D). Asymmetrical εNL_5.7_2_.0.0 (7h) reduced viability by 11.8% and 16.7% at 1.56 and 3.12 µM, respectively, and by 27.5% at 6.25 µM, the latter being significant versus DMSO (*p* ≤ 0.01); at this concentration, its cytotoxicity was comparable to that of 5-FUrd (Figure 4D). At 12.5 µM, viability dropped significantly by 58.5% (*p* ≤ 0.001 vs. DMSO), and cytotoxicity exceeded that of 5-FUrd by 24.9% (Figure 4D). The strongest effects occurred at 25 and 50 µM, where viability was reduced by 94.5% and 89.7%, respectively, both with *p* ≤ 0.001 versus DMSO (Figure 4D). These effects were also significant versus 5-FUrd, with *p* ≤ 0.001 at 25 µM and *p* ≤ 0.05 at 50 µM; cytotoxicity exceeded that of 5-FUrd by 59.7% and 46.8%, respectively (Figure 4D). Cyclic εNL_5.cycl7.0.0 (9) increased viability by 2.6% and 5.5% at 1.56 and 3.12 µM, respectively, but reduced it by 19.1% at 6.25 µM and significantly by 35.0% at 12.5 µM (*p* ≤ 0.01 vs. DMSO), with cytotoxicity comparable to 5-FUrd (Figure 4D). At 25 and 50 µM, viability was reduced by 91.1% and 95.0%, respectively, both with *p* ≤ 0.001 versus DMSO (Figure 4D). These reductions were also significant versus 5-FUrd, with *p* ≤ 0.001 at 25 µM and *p* ≤ 0.01 at 50 µM; the compound was 56.4% and 52.1% more cytotoxic than 5-FUrd at the respective concentrations (Figure 4D).

Asymmetrical εNL_5.8_3_.0.0 (7i) reduced viability by 11.5% at 1.56 µM and by 18.3% at 3.12 µM, followed by a smaller reduction of 13.7% at 6.25 µM (Figure 4D). At 12.5 µM, viability decreased by 38.1% relative to DMSO (*p* ≤ 0.01), and cytotoxicity exceeded that of 5-FUrd by 4.5% (Figure 4D). At 25 and 50 µM, viability was reduced by 77.8% and 85.3%, respectively (Figure 4D). These effects were significant compared with DMSO (both *p* ≤ 0.01) and 5-FUrd (both *p* ≤ 0.05), with cytotoxicity exceeding that of 5-FUrd by 43.1% and 42.3%, respectively (Figure 4D). εNL_5.9.0.0 (7d) showed no pronounced effects up to 6.25 µM, with reductions in viability of 3.4% at 1.56 µM and increases of 0.8% and 0.3% at 3.12 and 6.25 µM (Figure 4D). At 12.5 µM, viability was significantly reduced by 32.6% versus DMSO (*p* ≤ 0.01), while cytotoxicity was similar to that of 5-FUrd (Figure 4D). At 25 and 50 µM, viability declined by 92.9% and 89.3%, respectively, both with *p* ≤ 0.001 versus DMSO (Figure 4D). These effects were also significant compared with 5-FUrd, with *p* ≤ 0.001 at 25 µM and *p* ≤ 0.01 at 50 µM, and the compound was 58.2% and 46.4% more cytotoxic than 5-FUrd at 25 µM and 50 µM, respectively (Figure 4D). Symmetrical εNL_5.3.0.0 (7e) remained inactive up to 12.5 µM, with viability increased by 3.9% at 1.56 µM and only slightly reduced by 3.0%, 1.2%, and 3.8% at 3.12, 6.25, and 12.5 µM. At 25 and 50 µM, however, viability dropped dramatically to 0.6% and 0.0%, corresponding to cytotoxic effects of 99.4% and 100.0%, respectively (Figure 4D). Both effects were significant versus DMSO (both *p* ≤ 0.001) and also versus 5-FUrd, with *p* ≤ 0.001 at 25 µM and *p* ≤ 0.01 at 50 µM; cytotoxicity exceeded that of 5-FUrd by 64.7% and 57.0%, respectively (Figure 4D). Finally, asymmetrical εNL_5.3_2_.0.0 (7j) reduced viability by 8.1%, 19.4%, and 17.1% at 1.56, 3.12, and 6.25 µM, respectively (Figure 4D). At 12.5 µM, viability was reduced significantly by 33.5% (*p* ≤ 0.05 vs. DMSO) (Figure 4D). At 25 and 50 µM, viability was reduced by 51.0% and 65.6%, corresponding to *p* ≤ 0.01 and *p* ≤ 0.001 versus DMSO, respectively; at these concentrations, the compound was 16.3% and 12.7% more cytotoxic than 5-FUrd (Figure 4D).

Overall, the results in both glioma cell lines demonstrate a clear dependence of cytotoxicity on alkyl chain length, with maximal activity observed for medium-chain derivatives (C_15_–C_17_). In contrast, shorter and longer chains show reduced effects. Furthermore, 1,N^6^-ethenoadenosine derivatives generally exhibit enhanced cytotoxicity compared to their adenosine counterparts.

### 2.4. Cytotoxic Activity of Nucleolipid Derivatives in Human U87 and U251 Glioblastoma Cell Lines

The cytotoxic activity of selected nucleolipid derivatives was further evaluated in human glioblastoma cell lines U87 and U251 after 48 h of incubation. Temozolomide (TMZ), used as a standard chemotherapeutic agent in glioblastoma treatment, served as a reference control. Overall, TMZ exhibited only minor or no cytotoxic effects in both cell lines, whereas several nucleolipid derivatives showed pronounced and concentration-dependent reductions in cell viability, frequently exceeding the activity of TMZ.

#### 2.4.1. Cytotoxic Effects on Human U87 Glioblastoma Cells

**Figure 5:**
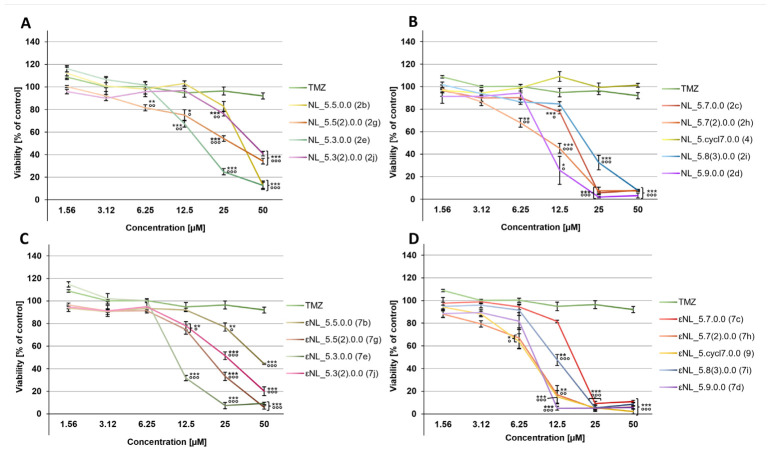
Viability (in % of control) of human U87 glioblastoma cells after 48 h incubation with TMZ (reference control) or nucleolipid derivatives. (**A**) Short- and long-chain adenosine derivatives: NL_5.5.0.0 (2b), NL_5.5_2_.0.0 (2g), NL_5.3.0.0 (2e), and NL_5.3_2_.0.0 (2j). (**B**) Medium- chain adenosine derivatives: NL_5.7.0.0 (2c), NL_5.7_2_.0.0 (2h), NL_5.cycl7.0.0 (4), NL_5.8_3_.0.0 (2i), NL_5.9.0.0 (2d). (**C**) Short- and long-chain 1,N^6^-ethenoadenosine derivatives: εNL_5.5.0.0 (7b), εNL_5.5_2_.0.0 (7g), εNL_5.3.0.0 (7e), and εNL_5.3_2_.0.0 (7j). (**D**) Medium-chain 1,N^6^-ethenoadenosine derivatives: εNL_5.7.0.0 (7c), εNL_5.7_2_.0.0 (7h), εNL_5.cycl7.0.0 (9), εNL_5.8_3_.0.0 (7i), εNL_5.9.0.0 (7d). Values are given as mean ± SEM (in % viability of control [incubation with medium alone = 100% viability]); * *p* ≤ 0.05, ** *p* ≤ 0.01, *** *p* ≤ 0.001 (significance vs. negative control [equal concentration of DMSO to substance solution, 0.5%]); ° *p* ≤ 0.05, °° *p* ≤ 0.01, °°° *p* ≤ 0.001 (significance vs. TMZ [equal concentration to substance solution]); *n* = 4 independent experiments assayed in quadruplicate.

In U87 glioblastoma cells, TMZ did not elicit significant cytotoxic effects after 48 h of treatment across the tested concentration range. At 1.56 µM, cell viability increased by 8.8%, while at 3.12 and 6.25 µM, viability remained essentially unchanged, with increases of 0.1% and 0.3%, respectively (Figure 5A–D). At higher concentrations, only minor reductions were observed: 5.2% at 12.5 µM, 3.6% at 25 µM, and 8.0% at 50 µM. These reference values are depicted in Figure 5A–D. For improved comparability, all cytotoxicity data are additionally summarized in Supplementary Table S1.

Among the short-chain adenosine derivatives, symmetrical NL_5.5.0.0 (2b), remained inactive at low concentrations, increasing viability by 12.1% at 1.56 µM and 3.1% at 12.5 µM, while only minor reductions of 0.8% and 1.9% were observed at 3.12 and 6.25 µM (Figure 5A). At higher concentrations, however, viability was reduced by 17.2% at 25 µM and by 87.0% at 50 µM (Figure 5A). The latter two effects were significant versus both DMSO and TMZ (both *p* ≤ 0.001), and at 50 µM, the compound was 79.0% more cytotoxic than TMZ (Figure 5A). Asymmetrical NL_5.5_2_.0.0 (2g) also showed concentration-dependent activity. Viability remained high at 1.56 µM, with only a 0.3% reduction, but then declined by 8.2% at 3.12 µM and by 18.5% at 6.25 µM (Figure 5A). At 12.5 µM, viability was significantly reduced by 24.9% versus both, DMSO and TMZ (*p* ≤ 0.05), and the compound was 19.7% more cytotoxic than TMZ (Figure 5A). At 25 µM, viability was reduced by 45.7%, an effect that was significant compared with DMSO and TMZ (*p* ≤ 0.001), exceeding the effect of TMZ by 42.1% (Figure 5A). The strongest effect was observed at 50 µM, where viability was reduced by 65.8%, again with *p* ≤ 0.001 versus both DMSO and TMZ, and with 57.8% higher cytotoxicity than TMZ (Figure 5A). NL_5.3.0.0 (2e) increased viability by 16.2%, 6.4%, and 1.7% at 1.56, 3.12, and 6.25 µM, respectively, but became strongly cytotoxic at higher concentrations (Figure 5A). Viability was reduced by 32.8% at 12.5 µM, 75.1% at 25 µM, and 87.2% at 50 µM; all three effects were significant compared with both DMSO and TMZ (*p* ≤ 0.001) (Figure 5A). At these concentrations, the effect exceeded that of TMZ by 27.6%, 71.5%, and 79.2%, respectively (Figure 5A). By contrast, NL_5.3_2_.0.0 (2j) remained weakly active up to 12.5 µM, reducing viability by 4.1% at 1.56 µM and by 10.1% at 3.12 µM, while viability was reduced by only 4.3% at 6.25 µM and by 3.2% at 12.5 µM. At 25 µM, viability declined by 23.7%, an effect significant versus DMSO (*p* ≤ 0.001) and TMZ (*p* ≤ 0.01), with the compound being 20.1% more cytotoxic than TMZ (Figure 5A). At 50 µM, viability was reduced by 59.1%, which was significant versus both DMSO and TMZ (*p* ≤ 0.001), and cytotoxicity exceeded that of TMZ by 51.1% (Figure 5A).

Among the medium- and long-chain adenosine derivatives, symmetrical NL_5.7.0.0 (2c) already showed increasing activity with rising concentration. Viability was reduced by 3.5% at 1.56 µM and by 10.1% at 3.12 µM, followed by reductions of 9.8% and 22.4% at 6.25 µM and 12.5 µM, respectively (Figure 5B). At 25 µM, viability dropped by 94.4%, and at 50 µM by 92.1%; both effects were significant versus DMSO and TMZ (*p* ≤ 0.001) (Figure 5B). At these concentrations, the compound was 90.8% and 84.1% more cytotoxic than TMZ, respectively (Figure 5B). Asymmetrical NL_5.7_2_.0.0 (2h) showed only minor effects at 1.56 and 3.12 µM, with reductions in viability of 2.0% and 13.5%, but became significantly active from 6.25 µM onward (Figure 5B). At 6.25 µM, viability was reduced by 32.0% (*p* ≤ 0.01 vs. both DMSO and TMZ) (Figure 5B). At 12.5 µM, viability was reduced by 54.7%; at 25 and 50 µM, by 92.5% and 92.6%, respectively; all three effects were significant versus both, DMSO and TMZ (*p* ≤ 0.001) (Figure 5B). At 25 and 50 µM, cytotoxicity exceeded that of TMZ by 88.9% and 84.6%, respectively (Figure 5B). In contrast, cyclic NL_5.cycl7.0.0 (4) remained inactive throughout, with only minor reductions in viability of 3.3% and 5.7% at 1.56 and 3.12 µM, no relevant change at 6.25 µM, and viability increases of 9.0%, 0.7%, and 1.3% at 12.5, 25, and 50 µM, respectively (Figure 5B).

NL_5.8_3_.0.0 (2i) reduced viability by 6.2% and 13.5% at 3.12 and 6.25 µM, while no relevant effect was seen at 1.56 µM, with increases of 1.7% (Figure 5B). At 12.5 µM, viability was reduced by 15.3%. Stronger effects occurred at 25 and 50 µM, where viability was reduced by 67.5% and 92.1%, respectively (Figure 5B). Both effects were significant versus the control groups (*p* ≤ 0.001), and cytotoxicity exceeded that of TMZ by 63.9% and 84.1%, respectively (Figure 5B). Symmetrical NL_5.9.0.0 (2d) reduced viability by 8.7%, 8.5%, and 5.7% at 1.56, 3.12, and 6.25 µM, respectively (Figure 5B). At 12.5 µM, viability decreased significantly by 69.1% compared with both DMSO and TMZ (*p* ≤ 0.05), exceeding the effect of TMZ by 69.1% (Figure 5B). At 25 and 50 µM, viability was reduced by 94.6% and 88.8%, respectively, and both effects were highly significant versus DMSO and TMZ (*p* ≤ 0.001) (Figure 5B). Interestingly, at these concentrations, cytotoxicity was also 94.6% and 88.8% higher than that of TMZ, respectively (Figure 5B).

Among the short-chain ethenoadenosine derivatives, symmetrical εNL_5.5.0.0 (7b) showed only minor effects up to 12.5 µM, with reductions of 6.4%, 9.5%, 6.6%, and 7.9% at 1.56, 3.12, 6.25, and 12.5 µM, respectively (Figure 5C). At 25 µM, viability was reduced by 23.1%, which was significant versus DMSO (*p* ≤ 0.01) and TMZ (*p* ≤ 0.05); at this concentration, the compound was 19.5% more cytotoxic than TMZ (Figure 5C). At 50 µM, viability declined by 55.7%, an effect that was significant compared with both DMSO and TMZ (*p* ≤ 0.001), and the effect exceeded that of TMZ by 47.7% (Figure 5C). Asymmetrical εNL_5.5_2_.0.0 (7g) reduced viability by 3.5%, 8.9%, and 8.6% at 1.56, 3.12, and 6.25 µM, respectively (Figure 5C). At 12.5 µM, viability was reduced by 24.4% compared with DMSO (*p* ≤ 0.01) and TMZ (*p* ≤ 0.05), and the compound was 20.2% more cytotoxic than TMZ (Figure 5C). At 25 and 50 µM, viability dropped by 66.8% and 94.3%, respectively, both with *p* ≤ 0.001 versus DMSO and TMZ (Figure 5C). Of note, at these concentrations, the effect exceeded that of TMZ by 63.2% and 86.3%, respectively (Figure 5C). εNL_5.3.0.0 (7e) showed no relevant toxicity at low concentrations, instead increasing viability by 14.7% at 1.56 µM and by 1.9% at 3.12 µM, while remaining essentially unchanged at 6.25 µM with a slight increase of 0.2% (Figure 5C). However, at 12.5 µM, viability was reduced by 68.3% compared with DMSO and TMZ (*p* ≤ 0.001), and cytotoxicity exceeded that of TMZ by 63.1% (Figure 5C). At 25 and 50 µM, viability was reduced by 92.6% and 90.7%, respectively, both with *p* ≤ 0.001 versus DMSO and TMZ; importantly, at these concentrations, the compound was 89.0% and 82.7% more cytotoxic than TMZ (Figure 5C). Likewise, the asymmetrical εNL_5.3_2_.0.0 (7j) remained weakly active at low concentrations, reducing viability by 5.4%, 8.8%, and 5.0% at 1.56, 3.12, and 6.25 µM, respectively (Figure 5C). At 12.5 µM, viability was reduced significantly by 22.0% versus DMSO (*p* ≤ 0.01) and TMZ (*p* ≤ 0.05), and cytotoxicity exceeded that of TMZ by 16.8% (Figure 5C). At 25 and 50 µM, viability declined by 48.5% and 79.9%, respectively, both with *p* ≤ 0.001 versus DMSO and TMZ, and the effects exceeded those of TMZ by 44.9% and 71.8%, respectively (Figure 5C).

Among the medium- and long-chain 1,N^6^-ethenoadenosine derivatives, symmetrical εNL_5.7.0.0 (7c) remained nearly inactive up to 12.5 µM, reducing viability by 2.4%, 1.0%, 5.7%, and 18.5% at 1.56, 3.12, 6.25, and 12.5 µM, respectively (Figure 5D). At 25 and 50 µM, however, viability dropped sharply by 90.8% and 89.1%, respectively, with both effects significant compared with DMSO and TMZ (*p* ≤ 0.001) (Figure 5D). Of note, at these concentrations, the compound was 87.2% and 81.1% more cytotoxic than TMZ. Asymmetrical εNL_5.7_2_.0.0 (7h) showed increasing activity already at lower concentrations, reducing viability by 12.0% at 1.56 µM and by 20.7% at 3.12 µM (Figure 5D). At 6.25 µM, viability declined significantly by 33.1% relative to both DMSO and TMZ (*p* ≤ 0.05), and cytotoxicity exceeded that of TMZ by 33.4% (Figure 5D). At 12.5 µM, viability dropped by 83.0%, a significant effect compared with DMSO and TMZ (*p* ≤ 0.01), and the compound was 77.8% more cytotoxic than TMZ (Figure 5D). At 25 and 50 µM, viability was reduced by 95.1% and 93.9%, respectively, both with *p* ≤ 0.001 versus DMSO and TMZ; At these concentrations, the effect exceeded that of TMZ by 91.5% and 85.9%, respectively (Figure 5D). Cyclic εNL_5.cycl7.0.0 (9) reduced viability by 5.8% and 11.8% at 1.56 and 3.12 µM, respectively (Figure 5D). At 6.25 µM, viability declined significantly by 36.0% relative to DMSO and TMZ (*p* ≤ 0.05), and cytotoxicity exceeded that of TMZ by 36.3% (Figure 5D). At 12.5 µM, viability was reduced by 85.0%, which was significant compared to both, DMSO and TMZ (*p* ≤ 0.001), and the compound was 79.7% more cytotoxic than TMZ (Figure 5D). At 25 and 50 µM, viability dropped by 94.7% and 97.9%, respectively, both with *p* ≤ 0.001 versus DMSO and TMZ; cytotoxicity exceeded that of TMZ by 91.1% and 89.9%, respectively (Figure 5D).

Asymmetrical εNL_5.8_3_.0.0 (7i) showed only weak effects up to 6.25 µM, with reductions of 5.0%, 4.2%, and 8.4% at 1.56, 3.12, and 6.25 µM, respectively (Figure 5D). At 12.5 µM, viability was reduced by 52.4%, which was significant versus DMSO (*p* ≤ 0.01) and TMZ (*p* ≤ 0.001); at this concentration, the compound was 47.2% more cytotoxic than TMZ (Figure 5D). At 25 and 50 µM, viability was reduced by 94.8% and 91.4%, respectively, both with *p* ≤ 0.001 versus DMSO and TMZ, and cytotoxicity exceeded that of TMZ by 91.2% and 83.4%, respectively (Figure 5D). Symmetrical εNL_5.9.0.0 (7d) reduced viability by 11.5% and 10.7% at 1.56 and 3.12 µM, followed by a reduction of 18.2% at 6.25 µM (Figure 5D). At 12.5 µM, viability decreased by 95.1%, a significant effect relative to both, DMSO and TMZ (*p* ≤ 0.001), and the effect exceeded that of TMZ by 89.9% (Figure 5D). At 25 and 50 µM, viability was reduced by 94.9% and 94.4%, respectively, again with *p* ≤ 0.001 versus DMSO and TMZ (Figure 5D). At these concentrations, the compound was 91.3% and 86.3% more cytotoxic than TMZ, respectively (Figure 5D).

#### 2.4.2. Cytotoxic Effects on Human U251 Glioblastoma Cells

**Figure 6:**
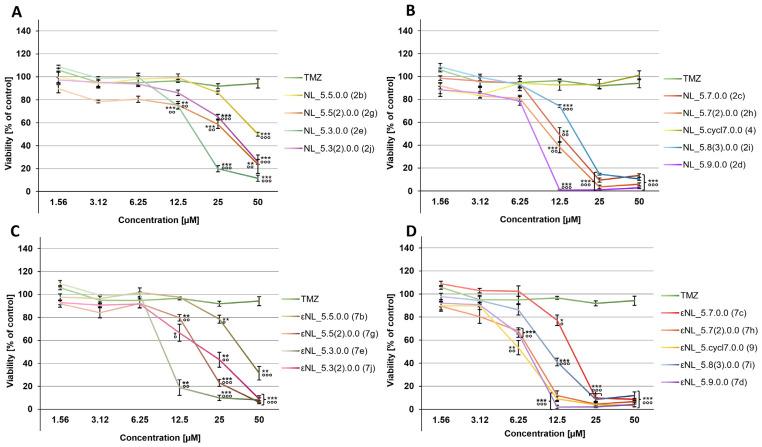
Viability (in % of control) of human U251 glioblastoma cells after 48 h incubation with TMZ (reference control) or nucleolipid derivatives. (**A**) Short- and long-chain adenosine derivatives: NL_5.5.0.0 (2b), NL_5.5_2_.0.0 (2g), NL_5.3.0.0 (2e), and NL_5.3_2_.0.0 (2j). (**B**) Medium- chain adenosine derivatives: NL_5.7.0.0 (2c), NL_5.7_2_.0.0 (2h), NL_5.cycl7.0.0 (4), NL_5.8_3_.0.0 (2i), NL_5.9.0.0 (2d). (**C**) Short- and long-chain 1,N^6^-ethenoadenosine derivatives: εNL_5.5.0.0 (7b), εNL_5.5_2_.0.0 (7g), εNL_5.3.0.0 (7e), and εNL_5.3_2_.0.0 (7j). (**D**) Medium-chain 1,N^6^-ethenoadenosine derivatives: εNL_5.7.0.0 (7c), εNL_5.7_2_.0.0 (7h), εNL_5.cycl7.0.0 (9), εNL_5.8_3_.0.0 (7i), εNL_5.9.0.0 (7d). Values are given as mean ± SEM (in % viability of control [incubation with medium alone = 100% viability]); * *p* ≤ 0.05, ** *p* ≤ 0.01, *** *p* ≤ 0.001 (significance vs. negative control [equal concentration of DMSO to substance solution, 0.5%]); ° *p* ≤ 0.05, °° *p* ≤ 0.01, °°° *p* ≤ 0.001 (significance vs. TMZ [equal concentration to substance solution]); *n* = 4 independent experiments assayed in quadruplicate.

In U251 glioblastoma cells, TMZ did not induce cytotoxic effects at any tested concentration after 48 h of treatment. Values remained stable across the entire concentration range, with values of 105.8%, 95.0%, 94.8%, 96.6%, 91.8%, and 94.2% at 1.56, 3.12, 6.25, 12.5, 25, and 50 µM, respectively, and no significant differences compared to the DMSO control. These reference data are illustrated in Figure 6A–D. For improved comparability, all cytotoxicity data are additionally summarized in Supplementary Table S1.

Among the short-chain adenosine derivatives, the symmetrical NL_5.5.0.0 (2b) exhibited only minor effects up to a concentration of 25 µM (Figure 6A). Cell viability was reduced by 0.3% at 1.56 µM, 6.3% at 3.12 µM, 1.8% at 6.25 µM, and 0.7% at 12.5 µM, while a more pronounced decline of 13.7% was observed at 25 µM (Figure 6A). At the highest concentration of 50 µM, viability decreased markedly to 50.1%, corresponding to a 49.9% reduction, which was significant compared to both, DMSO and TMZ (*p* ≤ 0.001) (Figure 6A). Interestingly, at this concentration, the compound exceeded the effect of TMZ by 44.1% (Figure 6A). Asymmetrical NL_5.5_2_.0.0 (2g) showed a clearer concentration-dependent response, reducing viability by 10.4%, 21.5%, and 19.5% at 1.56, 3.12, and 6.25 µM, respectively (Figure 6A). At 12.5 µM, cell viability was reduced by 24.8%, which was significant versus DMSO and TMZ (*p* ≤ 0.01), and cytotoxicity exceeded that of TMZ by 21.4%. At 25 µM, viability decreased to 58.7% (a reduction of 41.3%), and the highest efficacy was observed at 50 µM, where viability was diminished to 23.9%, corresponding to a 76.1% reduction; this was significant compared to both, DMSO and TMZ (*p* ≤ 0.01), and the effect surpassed that of TMZ by 70.3% (Figure 6A). NL_5.3.0.0 (2e) increased viability by 8.9% at 1.56 µM and remained near the control at 3.12 and 6.25 µM, with reductions of 1.3% and 0.4%, respectively (Figure 6A). At 12.5 µM, viability decreased by 25.6%, which was significant compared to DMSO (*p* ≤ 0.001) and TMZ (*p* ≤ 0.01), and the effect exceeded that of TMZ by 22.2% (Figure 6A). At 25 and 50 µM, cell viability dropped sharply to 19.9% and 11.5%, corresponding to reductions of 80.1% and 88.5%, respectively; both effects were significant versus DMSO and TMZ (*p* ≤ 0.001), and the effects exceeded those of TMZ by 71.9% and 82.6% (Figure 6A). Asymmetrical NL_5.3_2_.0.0 (2j) displayed minimal activity at lower concentrations, with reductions in viability of 2.7%, 4.8%, 6.4%, and 10.9% at 1.56, 3.12, 6.25, and 12.5 µM, respectively (Figure 6A). At 25 and 50 µM, viability was reduced by 34.8% and 74.1%, respectively; both effects were significant compared to DMSO and TMZ (*p* ≤ 0.001), and cytotoxicity exceeded that of TMZ by 26.7% and 68.3%, respectively (Figure 6A).

Among the medium- and long-chain adenosine derivatives, the symmetrical derivative NL_5.7.0.0 (2c) exhibited only minor effects at 1.56 and 3.12 µM, reducing cell viability by 1.4% and 4.0%, respectively, followed by a 5.8% reduction at 6.25 µM (Figure 6B). At 12.5 µM, a marked decrease in viability to 49.0% was observed, corresponding to a 51.0% reduction (Figure 6B). At 25 and 50 µM, viability further declined to 9.5% and 13.5%, corresponding to reductions of 90.5% and 86.5%, respectively (Figure 6B). The high-dose effects at 25 and 50 µM were statistically significant compared to both DMSO and TMZ (*p* ≤ 0.001), and cytotoxicity exceeded that of TMZ by 82.3% and 80.7%, respectively (Figure 6B). Asymmetrical NL_5.7_2_.0.0 (2h) induced moderate reductions in viability of 8.1%, 16.9%, and 19.1% at 1.56, 3.12, and 6.25 µM, respectively (Figure 6B). At 12.5 µM, viability dropped to 38.2%, representing a 61.8% reduction, which was significant compared to both DMSO and TMZ (*p* ≤ 0.001); at this concentration, cytotoxicity surpassed that of TMZ by 58.3% (Figure 6B). At 25 and 50 µM, viability declined to 3.5% and 5.9%, corresponding to reductions of 96.5% and 94.1%; both effects were significant compared to DMSO and TMZ (*p* ≤ 0.001) (Figure 6B). At 25 µM, the compound was again 58.3% more cytotoxic than TMZ, and at 50 µM, cytotoxicity exceeded that of TMZ by 88.3% (Figure 6B). In contrast, the cyclic derivative NL_5.cycl7.0.0 (4) remained inactive throughout the tested range, with viability values of 96.7%, 94.3%, 99.0%, 109.0%, 99.3%, and 101.3% from 1.56 to 50 µM, and no significant decreases (Figure 6B).

The asymmetrical derivative NL_5.8_3_.0.0 (2i) exhibited only minor effects at lower concentrations, increasing cell viability by 8.6% at 1.56 µM, remaining close to the control at 3.12 µM with a 0.7% reduction, and decreasing viability by 7.0% at 6.25 µM (Figure 6B). At 12.5 µM, viability was reduced by 25.9%, which was significant compared to both DMSO and TMZ (*p* ≤ 0.001), and cytotoxicity exceeded that of TMZ by 22.4% (Figure 6B). At 25 and 50 µM, viability dropped to 14.8% and 10.6%, corresponding to reductions of 85.2% and 89.4%, respectively; both effects were significant compared to DMSO and TMZ (*p* ≤ 0.001), and cytotoxicity exceeded that of TMZ by 77.1% and 83.6% (Figure 6B). Symmetrical NL_5.9.0.0 (2d) reduced cell viability by 11.5%, 14.3%, and 21.4% at 1.56, 3.12, and 6.25 µM, respectively (Figure 6B). At 12.5 µM, viability dropped dramatically to 0.9%, corresponding to a 99.1% reduction, which was significant compared to both, DMSO and TMZ (*p* ≤ 0.001); at this concentration, the compound was 95.7% more cytotoxic than TMZ (Figure 6B). At 25 and 50 µM, values remained very low at 1.0% and 2.7%, corresponding to cytotoxic effects of 99.0% and 97.3%, respectively; both effects were significant compared to DMSO and TMZ (*p* ≤ 0.001), and cytotoxicity exceeded that of TMZ by 90.8% and 91.5% (Figure 6B).

Among the short-chain 1,N^6^-ethenoadenosine derivatives, symmetrical εNL_5.5.0.0 (7b) showed little activity up to 12.5 µM, reducing viability by 2.4%, 3.5%, and 2.5% at 1.56, 3.12, and 12.5 µM, while viability increased by 1.7% at 6.25 µM (Figure 6C). At 25 and 50 µM, viability declined to 78.5% and 31.4%, respectively, corresponding to reductions of 21.5% and 68.6%, respectively; both effects were significant compared with DMSO and TMZ (*p* ≤ 0.01) (Figure 6C). At these concentrations, the effect exceeded that of TMZ by 13.4% and 62.8%, respectively (Figure 6C). Asymmetrical εNL_5.5_2_.0.0 (7g) reduced viability by 8.1% at 1.56 µM and by 15.9% at 3.12 µM, whereas at 6.25 µM, viability partly recovered, with only a 7.8% reduction (Figure 6C). At 12.5 µM, viability dropped to 79.7%, a 20.3% reduction (Figure 6C). At 25 and 50 µM, viability was reduced by 77.1% and 93.9%, respectively; both high-dose effects were significant versus DMSO and TMZ (*p* ≤ 0.001), and exceeded those of TMZ by 68.9% and 88.0%, respectively (Figure 6C). The symmetrical derivative εNL_5.3.0.0 (7e) increased viability by 9.4% at 1.56 µM and remained close to control at 3.12 and 6.25 µM, with values corresponding to a reduction of 0.8% and an increase of 0.1%, respectively (Figure 6C). At 12.5 µM, viability dropped sharply to 18.9%, corresponding to a cytotoxic effect of 81.1%. At 25 and 50 µM, viability declined further to 9.9% and 8.0%, corresponding to reductions of 90.1% and 92.0%, respectively (Figure 6C). These effects were significant compared with both, DMSO and TMZ (*p* ≤ 0.001), and the effects exceeded those of TMZ by 81.9% at 25 µM and 86.1% at 50 µM (Figure 6C). Asymmetrical εNL_5.3_2_.0.0 (7j) reduced viability by 7.2%, 9.3%, and 8.4% at 1.56, 3.12, and 6.25 µM, respectively (Figure 6C). At 12.5 µM, viability dropped to 66.6%, a 33.4% reduction (Figure 6C). At 25 and 50 µM, viability was reduced by 56.8% and 91.1%, respectively (Figure 6C). These reductions were significant versus DMSO and TMZ (*p* ≤ 0.001), and at 50 µM, cytotoxicity exceeded that of TMZ by 85.3% (Figure 6C).

Among the medium- and long-chain ethenoadenosine derivatives, symmetrical pentadecanyl derivative εNL_5.7.0.0 (7c) increased viability by 8.8%, 2.9%, and 2.5% at 1.56, 3.12, and 6.25 µM, respectively (Figure 6D). At 12.5 µM, viability dropped to 77.3%, corresponding to a 22.7% reduction, which was significant compared with DMSO and TMZ (*p* ≤ 0.05); at this concentration, cytotoxicity exceeded that of TMZ by 19.3% (Figure 6D). At 25 and 50 µM, viability declined sharply to 9.7% and 8.7%, corresponding to reductions of 90.3% and 91.3%, respectively; both effects were significant versus DMSO and TMZ (*p* ≤ 0.001), and cytotoxicity exceeded that of TMZ by 82.1% and 85.4%, respectively (Figure 6D). Asymmetrical εNL_5.7_2_.0.0 (7h) reduced viability by 10.6% at 1.56 µM and by 19.5% at 3.12 µM (Figure 6D). At 6.25 µM, viability dropped significantly to 67.9%, representing a 32.1% reduction; this effect was significant versus DMSO (*p* ≤ 0.001) and TMZ (*p* ≤ 0.01), and cytotoxicity exceeded that of TMZ by 26.9% (Figure 6D). At 12.5 µM, viability fell to 11.8%, corresponding to an 88.2% reduction, which was significant compared with both DMSO and TMZ (*p* ≤ 0.001); at this concentration, the compound was 84.7% more cytotoxic than TMZ (Figure 6D). At 25 and 50 µM, viability declined to 4.5% and 6.6%, corresponding to cytotoxic effects of 95.5% and 93.4%, respectively; both effects were significant versus DMSO and TMZ (*p* ≤ 0.001), and the effects exceeded those of TMZ by 87.4% and 87.5%, respectively (Figure 6D). Cyclic εNL_5.cycl7.0.0 (9) reduced viability by 10.3% and 10.4% at 1.56 and 3.12 µM, respectively (Figure 6D). At 6.25 µM, viability dropped significantly to 53.5%, corresponding to a 46.5% reduction compared with control (*p* ≤ 0.01 vs. DMSO and TMZ), and cytotoxicity exceeded that of TMZ by 41.4% (Figure 6D). At 12.5 µM, viability decreased further to 9.0%, corresponding to a 91.0% reduction, which was significant versus compared to DMSO and TMZ (*p* ≤ 0.001), and cytotoxicity exceeded that of TMZ by 87.6% (Figure 6D). At 25 and 50 µM, viability fell to 4.0% and 3.3%, corresponding to reductions of 96.0% and 96.7%; both effects were significant versus DMSO and TMZ (*p* ≤ 0.001), and cytotoxicity exceeded that of TMZ by 87.8% and 90.9%, respectively (Figure 6D).

εNL_5.8_3_.0.0 (7i) remained only weakly active at low concentrations, reducing viability by 2.2%, 5.6%, and 13.8% at 1.56, 3.12, and 6.25 µM, respectively (Figure 6D). At 12.5 µM, viability dropped to 41.0%, corresponding to a 59.0% reduction; this effect was significant compared with DMSO and TMZ (*p* ≤ 0.001), and the effect exceeded that of TMZ by 55.6% (Figure 6D). At 25 and 50 µM, viability declined further to 8.6% and 11.9%, corresponding to reductions of 91.4% and 88.1%, respectively (Figure 6D). Both effects were significant compared with DMSO and TMZ (*p* ≤ 0.001), and cytotoxicity exceeded that of TMZ by 83.3% at 25 µM and 82.2% at 50 µM (Figure 6D). The symmetrical derivative εNL_5.9.0.0 (7d) reduced viability by 8.0% at 1.56 µM and by 9.4% at 3.12 µM. At 6.25 µM, viability dropped significantly to 65.6%, corresponding to a 34.4% reduction, which was significant compared with DMSO and TMZ (*p* ≤ 0.001), and cytotoxicity exceeded that of TMZ by 29.2% (Figure 6D). At 12.5, 25, and 50 µM, viability declined dramatically to 2.0%, 2.2%, and 4.1%, corresponding to reductions of 98.0%, 97.8%, and 95.9%, respectively (Figure 6D). All three effects were significant versus DMSO and TMZ (*p* ≤ 0.001), and cytotoxicity exceeded that of TMZ by 94.6%, 89.6%, and 90.1%, respectively (Figure 6D).

Overall, the results in U87 cells demonstrate a strong dependence of cytotoxicity on alkyl chain length, with maximal activity observed for medium-chain derivatives (C_15_–C_17_). In contrast, shorter and longer chains showed reduced or more variable effects. A comparable pattern was observed in U251 cells, confirming this structure–activity relationship across both glioblastoma models. In addition, 1,N^6^-ethenoadenosine derivatives generally exhibited enhanced cytotoxicity compared to the corresponding adenosine analogs. Notably, several compounds clearly exceeded the cytotoxic activity of the reference compound TMZ, which showed only minor effects under the applied conditions.

### 2.5. Cytotoxic Effects on PMA-Differentiated Human THP-1 Macrophages

To evaluate potential cytotoxic side effects in non-tumor cells, the nucleolipid derivatives were investigated in PMA-differentiated human THP-1 macrophages.

**Figure 7:**
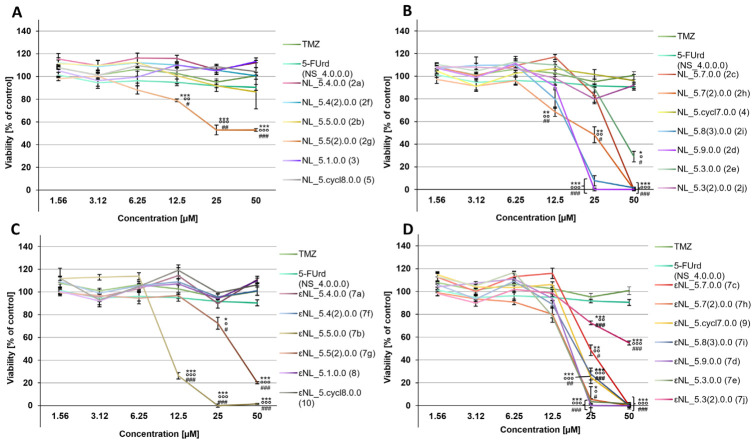
Viability (in % of control) of PMA-differentiated human THP-1 macrophages after 48 h incubation with NS_4.0.0.0 (5-FUrd; reference control), TMZ (reference control) or nucleolipid derivatives. (**A**) Short-chain and bulky adenosine derivatives: NL_5.4.0.0 (2a), NL_5.4_2_.0.0 (2f), NL_5.5.0.0 (2b), NL_5.5_2_.0.0 (2g), NL_5.1.0.0 (3), and NL_5.cycl8.0.0 (5). (**B**) Medium- and long-chain adenosine derivatives: NL_5.7.0.0 (2c), NL_5.7_2_.0.0 (2h), NL_5.cycl7.0.0 (4), NL_5.8_3_.0.0 (2i), NL_5.9.0.0 (2d), NL_5.3.0.0 (2e), and NL_5.3_2_.0.0 (2j). (**C**) Short-chain and bulky 1,N^6^-ethenoadenosine derivatives: εNL_5.4.0.0 (7a), εNL_5.4_2_.0.0 (7f), εNL_5.5.0.0 (7b), εNL_5.5_2_.0.0 (7g), εNL_5.1.0.0 (8), and εNL_5.cycl8.0.0 (10). (**D**) Medium- and long-chain 1,N^6^-ethenoadenosine derivatives: εNL_5.7.0.0 (7c), εNL_5.7_2_.0.0 (7h), εNL_5.cycl7.0.0 (9), εNL_5.8_3_.0.0 (7i), εNL_5.9.0.0 (7d), εNL_5.3.0.0 (7e), and εNL_5.3_2_.0.0 (7j). Values are given as mean ± SEM (in % viability of control [incubation with medium alone = 100% viability]); * *p* ≤ 0.05, ** *p* ≤ 0.01, *** *p* ≤ 0.001 (significance vs. negative control [equal concentration of DMSO to substance solution, 0.5%]); ^#^ *p* ≤ 0.05, ^##^ *p* ≤ 0.01, ^###^ *p* ≤ 0.001 (significance vs. 5-FUrd [equal concentration to substance solution]); ° *p* ≤ 0.05, °° *p* ≤ 0.01, °°° *p* ≤ 0.001 (significance vs. TMZ [equal concentration to substance solution]); *n* = 4 independent experiments assayed in quadruplicate.

First, the effects of the reference compounds were assessed. Treatment with 5-FUrd resulted in only minor reductions in cell viability across the tested concentration range, with values decreasing from 101.3% at 1.56 µM to 90.4% at 50 µM (Figure 7A–D). Similarly, TMZ showed no significant cytotoxicity, with viability ranging from 95.1% to 107.8% across all concentrations. These results confirm the low sensitivity of THP-1 macrophages toward the reference chemotherapeutics under the applied conditions. These reference data are shown throughout Figure 7A–D. For improved comparability, all cytotoxicity data are additionally summarized in Supplementary Table S1.

Among the short-chain and bulky adenosine derivatives, symmetrical NL_5.4.0.0 (2a) did not reduce macrophage viability at any tested concentration. Instead, viability remained elevated throughout, increasing by 15.4%, 9.6%, 16.3%, 15.9%, 5.9%, and 12.2% from 1.56 to 50 µM (Figure 7A). A similar pattern was observed for asymmetrical NL_5.4_2_.0.0 (2f), which increased viability by 12.1%, 8.7%, 12.1%, 10.2%, 5.6%, and 0.7% across the full concentration range (Figure 7A). The symmetrical derivative NL_5.5.0.0 (2b) also remained non-cytotoxic, with viability increased by 11.3%, 9.9%, and 12.6% at 1.56, 3.12, and 6.25 µM, followed by values close to control at higher concentrations, namely 100.6%, 91.8%, and 86.4% at 12.5, 25, and 50 µM (Figure 7A). Asymmetrical NL_5.1.0.0 (3) likewise did not impair viability, showing values of 105.0%, 96.4%, 99.5%, 110.1%, 105.1%, and 113.2% (Figure 7A). The same was observed for NL_5.cycl8.0.0 (5), which maintained viability above control or close to control at all concentrations, with values of 107.5%, 101.0%, 109.6%, 105.0%, 108.6%, and 104.3% (Figure 7A).

In contrast, asymmetrical NL_5.5_2_.0.0 (2g) was the first adenosine derivative to show marked cytotoxicity toward THP-1 macrophages. Viability remained close to control at 1.56 and 3.12 µM, corresponding to reductions of 2.0% and increases of 0.2%, respectively, but declined by 11.9% at 6.25 µM (Figure 7A). At 12.5 µM, viability was reduced by 21.0%, which was significant versus DMSO (*p* ≤ 0.001), 5-FUrd (*p* ≤ 0.05), and TMZ (*p* ≤ 0.01); at this concentration, the compound was 15.8% more cytotoxic than 5-FUrd and 23.6% more cytotoxic than TMZ (Figure 7A). At 25 µM, viability dropped to 53.0%, corresponding to a reduction of 47.0%; this effect was significant versus DMSO (*p* ≤ 0.001), 5-FUrd (*p* ≤ 0.01), and TMZ (*p* ≤ 0.001), and cytotoxicity exceeded that of 5-FUrd by 38.7% and that of TMZ by 42.2% (Figure 7A). At 50 µM, viability remained low at 52.9%, corresponding to a reduction of 47.1%; this effect was significant versus DMSO, 5-FUrd, and TMZ (all *p* ≤ 0.001), and cytotoxicity exceeded that of 5-FUrd by 37.5% and that of TMZ by 47.8% (Figure 7A).

Among the medium- and long-chain adenosine derivatives, symmetrical derivative NL_5.7.0.0 (2c) increased viability at lower concentrations, with values of 108.3%, 100.5%, 109.4%, and 117.0% at 1.56, 3.12, 6.25, and 12.5 µM, respectively (Figure 7B). At 25 µM, viability was reduced by 16.7%, and at 50 µM it dropped to 0.0%, corresponding to a cytotoxic effect of 100.0% (Figure 7B). This high-dose effect was significant versus DMSO, 5-FUrd, and TMZ (*p* ≤ 0.001), and cytotoxicity exceeded that of 5-FUrd by 90.4% and that of TMZ by 100.7% (Figure 7B). Asymmetrical NL_5.7_2_.0.0 (2h) showed weak effects up to 6.25 µM, with reductions of 2.8%, 8.7%, and 4.0% (Figure 7B). At 12.5 µM, viability dropped to 68.6%, corresponding to a reduction of 31.4%, which was significant versus DMSO, 5-FUrd, and TMZ (*p* ≤ 0.01); at this concentration, the compound was 26.6% more cytotoxic than 5-FUrd and 34.0% more cytotoxic than TMZ (Figure 7B). At 25 µM, viability declined to 48.3%, corresponding to a reduction of 51.7%; this effect was significant versus DMSO and TMZ (*p* ≤ 0.01) and versus 5-FUrd (*p* ≤ 0.05), and cytotoxicity exceeded that of 5-FUrd by 43.4% and that of TMZ by 46.8% (Figure 7B). At 50 µM, viability was 0.0%, corresponding to a cytotoxic effect of 100.0%; this effect was significant versus DMSO, 5-FUrd, and TMZ (*p* ≤ 0.001), and cytotoxicity exceeded that of 5-FUrd by 90.4% and that of TMZ by 100.6% (Figure 7B). By contrast, cyclic pentadecanyl derivative NL_5.cycl7.0.0 (4) remained non-cytotoxic, with viability values of 104.6%, 91.0%, 102.7%, 106.6%, 101.5%, and 96.1% across the full concentration range (Figure 7B).

The asymmetrical derivative NL_5.8_3_.0.0 (2i) increased viability by 6.8%, 9.7%, and 9.9% at 1.56, 3.12, and 6.25 µM, respectively, but became strongly cytotoxic at higher concentrations (Figure 7B). At 12.5 µM, viability dropped to 79.9%, corresponding to a 20.1% reduction (Figure 7B). At 25 µM, viability declined sharply to 7.7%, representing a cytotoxic effect of 92.3%; this effect was significant versus DMSO, 5-FUrd, and TMZ (*p* ≤ 0.001), and cytotoxicity exceeded that of 5-FUrd by 83.9% and that of TMZ by 87.4% (Figure 7B). At 50 µM, viability further decreased to 1.5%, corresponding to a 98.5% reduction; this effect was likewise significant versus DMSO, 5-FUrd, and TMZ (*p* ≤ 0.001), and cytotoxicity exceeded that of 5-FUrd by 88.9% and that of TMZ by 99.2% (Figure 7B). Symmetrical NL_5.9.0.0 (2d) showed values above or close to control up to 12.5 µM, namely 107.2%, 98.9%, 111.5%, and 91.0% (Figure 7B). At 25 µM, viability was 0.0%, corresponding to a cytotoxic effect of 100.0%, and at 50 µM, viability was 4.6%, corresponding to a reduction of 95.4% (Figure 7B). Both effects were significant versus DMSO, 5-FUrd, and TMZ (*p* ≤ 0.001); cytotoxicity exceeded that of 5-FUrd by 91.6% and 85.9%, and that of TMZ by 95.1% and 96.1%, respectively (Figure 7B). NL_5.3.0.0 (2e) remained non-cytotoxic up to 25 µM, with viability values of 110.5%, 104.6%, 112.6%, 109.9%, and 89.4% (Figure 7B). At 50 µM, however, viability dropped to 29.1%, corresponding to a 70.9% reduction; this effect was significant compared with DMSO, 5-FUrd, and TMZ (*p* ≤ 0.05), and cytotoxicity exceeded that of 5-FUrd by 61.3% and that of TMZ by 71.6% (Figure 7B). NL_5.3_2_.0.0 (2j) likewise remained largely inactive, with viability values of 108.4%, 108.0%, 106.7%, 98.1%, 79.9%, and 92.0% from 1.56 to 50 µM; although viability dropped by 20.1% at 25 µM, no significance was reported (Figure 7B).

Among the short-chain and bulky ethenoadenosine derivatives, symmetrical εNL_5.4.0.0 (7a) remained non-cytotoxic, with viability values of 100.2%, 95.6%, 102.8%, 114.3%, 89.9%, and 110.9% from 1.56 to 50 µM (Figure 7C). Similarly, asymmetrical εNL_5.4_2_.0.0 (7f) showed no cytotoxicity, with viability values of 109.2%, 99.0%, 105.6%, 109.0%, 95.6%, and 101.0% (Figure 7C). In contrast, symmetrical εNL_5.5.0.0 (7b) showed pronounced concentration-dependent toxicity. Viability was elevated at low concentrations, reaching 111.7%, 112.9%, and 113.8% at 1.56, 3.12, and 6.25 µM, respectively, but dropped sharply to 26.3% at 12.5 µM, a 73.7% reduction (Figure 7C). This effect was significant versus DMSO, 5-FUrd, and TMZ (*p* ≤ 0.001), and cytotoxicity exceeded that of 5-FUrd by 68.6% and TMZ by 76.3% (Figure 7C). At 25 µM, viability was 0.0%, corresponding to a cytotoxic effect of 100.0%, and at 50 µM it was 1.5%, corresponding to a reduction of 98.5%; both effects were significant versus DMSO, 5-FUrd, and TMZ (*p* ≤ 0.001) (Figure 7C). At these concentrations, cytotoxicity exceeded that of 5-FUrd by 91.6% and 88.9%, and that of TMZ by 95.1% and 99.2%, respectively (Figure 7C). The asymmetrical derivative εNL_5.5_2_.0.0 (7g) showed only minor effects up to 12.5 µM, reducing viability by 0.8%, 3.1%, 5.5%, and 3.3% (Figure 7C). At 25 µM, viability dropped to 72.5%, corresponding to a 27.5% reduction; this effect was significant compared with DMSO, 5-FUrd, and TMZ (*p* ≤ 0.05), and cytotoxicity exceeded that of 5-FUrd by 19.2% and that of TMZ by 22.7% (Figure 7C). At 50 µM, viability was further decreased to 20.2%, corresponding to a 79.8% reduction; this effect was significant compared with DMSO, 5-FUrd, and TMZ (*p* ≤ 0.001), and cytotoxicity exceeded that of 5-FUrd by 70.2% and that of TMZ by 80.5% (Figure 7C).

The asymmetrical derivative εNL_5.1.0.0 (8) and symmetrical derivative εNL_5.cycl8.0.0 (10) both showed no cytotoxicity, with viability remaining above 92% across all tested concentrations (Figure 7C).

Among the medium- and long-chain ethenoadenosine derivatives, symmetrical εNL_5.7.0.0 (7c) increased viability at low concentrations, reaching 112.6%, 100.1%, 113.1%, and 116.0% at 1.56, 3.12, 6.25, and 12.5 µM, respectively (Figure 7D). At 25 µM, viability dropped to 48.5%, corresponding to a reduction of 51.5%; this effect was significant versus DMSO and TMZ (*p* ≤ 0.01) and versus 5-FUrd (*p* ≤ 0.05), and cytotoxicity exceeded that of 5-FUrd by 43.2% and that of TMZ by 46.6% (Figure 7D). At 50 µM, viability was 0.0%, corresponding to a cytotoxic effect of 100.0%; this effect was significant versus DMSO, 5-FUrd, and TMZ (*p* ≤ 0.001), and cytotoxicity exceeded that of 5-FUrd by 90.4% and that of TMZ by 100.7% (Figure 7D). The asymmetrical derivative εNL_5.7_2_.0.0 (7h) showed only weak reductions at low concentrations, namely 1.1%, 6.5%, and 9.1% at 1.56, 3.12, and 6.25 µM. At 12.5 µM, viability dropped to 80.0%, a 20.0% reduction (Figure 7D). At 25 µM, viability decreased strongly to 5.5%, corresponding to a cytotoxic effect of 94.5%; this effect was significant versus DMSO, 5-FUrd, and TMZ (*p* ≤ 0.01), and cytotoxicity exceeded that of 5-FUrd by 86.2% and that of TMZ by 89.7% (Figure 7D). At 50 µM, viability was 0.0%, corresponding to a cytotoxic effect of 102.9%; this effect was significant versus DMSO, 5-FUrd, and TMZ (*p* ≤ 0.001), and cytotoxicity exceeded that of 5-FUrd by 93.3% and that of TMZ by 103.6% (Figure 7D). Cyclic εNL_5.cycl7.0.0 (9) increased viability at lower concentrations to 114.6%, 104.0%, 103.5%, and 106.1% at 1.56, 3.12, 6.25, and 12.5 µM (Figure 7D). At 25 µM, viability declined to 24.8%, corresponding to a reduction of 75.2%; this effect was significant versus DMSO and TMZ (*p* ≤ 0.001) and versus 5-FUrd (*p* ≤ 0.01), and cytotoxicity exceeded that of 5-FUrd by 66.8% and that of TMZ by 70.3% (Figure 7D). At 50 µM, viability dropped further to 0.0%, corresponding to a cytotoxic effect of 100.0%; this effect was significant versus DMSO, 5-FUrd, and TMZ (*p* ≤ 0.001), and cytotoxicity exceeded that of 5-FUrd by 90.4% and that of TMZ by 100.7% (Figure 7D).

Asymmetrical εNL_5.8_3_.0.0 (7i) showed no meaningful toxicity up to 12.5 µM, with viability values of 106.2%, 93.4%, 109.0%, and 88.9% (Figure 7D). At 25 µM, viability declined to 27.7%, corresponding to a 72.3% reduction; this effect was significant versus DMSO, 5-FUrd, and TMZ (*p* ≤ 0.001), and cytotoxicity exceeded that of 5-FUrd by 64.0% and that of TMZ by 67.5% (Figure 7D). At 50 µM, viability dropped to 0.0%, corresponding to a cytotoxic effect of 100.0%; this effect was also significant versus DMSO, 5-FUrd, and TMZ (*p* ≤ 0.001), and cytotoxicity exceeded that of 5-FUrd by 90.4% and that of TMZ by 100.6% (Figure 7D). The derivative εNL_5.9.0.0 (7d) increased viability at low and intermediate concentrations, reaching 104.1%, 107.3%, and 111.0% at 1.56, 3.12, and 6.25 µM, respectively, but dropped to 93.8% at 12.5 µM (Figure 7D). At 25 and 50 µM, viability dropped below 0.0%, corresponding to 100.0% cytotoxicity and indicating extremely strong cytotoxic effects. Both concentrations differed significantly from DMSO, 5-FUrd, and TMZ (*p* ≤ 0.001) (Figure 7D). Compared with 5-FUrd, the compound showed 91.6% stronger effects at 25 µM and 90.4% greater effect at 50 µM. Relative to TMZ, the effects were increased by 95.1% at 25 µM and by 100.7% at 50 µM (Figure 7D). εNL_5.3.0.0 (7e) also showed no relevant activity at lower concentrations, with viability values of 112.9%, 104.9%, and 116.8% at 1.56, 3.12, and 6.25 µM, before dropping to 78.4% at 12.5 µM, corresponding to a reduction of 21.6% (Figure 7D). At 25 and 50 µM, viability declined sharply to 3.5% and 1.6%, corresponding to cytotoxic effects of 96.5% and 98.4%, respectively (Figure 7D). Both concentrations were significant versus DMSO, 5-FUrd, and TMZ (*p* ≤ 0.001), and the effects exceeded those of 5-FUrd by 88.2% and 88.8% and those of TMZ by 91.7% and 99.1%, respectively (Figure 7D). Finally, asymmetrical εNL_5.3_2_.0.0 (7j) showed only minor effects up to 12.5 µM, with viability values of 98.2%, 90.0%, 101.8%, and 98.6% (Figure 7D). At 25 µM, viability dropped to 72.5%, corresponding to a reduction of 27.5%; this effect was significant versus DMSO and 5-FUrd (*p* ≤ 0.001) and versus TMZ (*p* ≤ 0.01), and cytotoxicity exceeded that of 5-FUrd by 19.1% and that of TMZ by 22.6% (Figure 7D). At 50 µM, viability declined further to 54.9%, corresponding to a reduction of 45.1%; this effect was significant versus DMSO, 5-FUrd, and TMZ (*p* ≤ 0.001), and the effect exceeded those of 5-FUrd and TMZ by 35.5% and 45.8%, respectively (Figure 7D).

Overall, the results demonstrate a strong dependence of cytotoxic effects in PMA-differentiated human THP-1 macrophages on alkyl chain length and molecular structure. While several medium-chain derivatives exhibit pronounced cytotoxicity at higher concentrations, short-chain and bulky derivatives show minimal or no effects. Importantly, although some compounds exhibit greater cytotoxicity than 5-FUrd and TMZ, this effect is primarily observed at higher concentrations and follows a clear structure–activity relationship, indicating that the observed activity is not solely due to nonspecific toxicity.

## 3. Discussion

Our novelly synthesized nucleoside derivatives based on adenosine and 1,N^6^-ethenoadenosine exhibit pronounced cytotoxicity in glioma and glioblastoma cell lines (BT4Ca, GOS-3, U87, U251) from humans or rats, clearly exceeding those of the reference compounds 5-FUrd and TMZ. The consistent activity observed across multiple tumor cell models of different origins underlines the robustness of this effect and highlights the broad antitumor potential of this compound class. Several of the nucleolipid derivatives under test induced a markedly stronger reduction in cell viability than both reference compounds, particularly at very low concentrations ≥ 12.5 µM, in some cases resulting in an almost complete loss of viable cells. These findings emphasize the potential of lipophilized nucleoside analogs as promising candidates for developing of novel therapeutic strategies targeting malignant brain tumors. The pharmacological and potentially therapeutical relevance of this compound class is further supported by an existing patent (EP 4 342 905 A1), underlining its innovative and translational potential.

In contrast to the reference compounds, which are small molecules with limited structural flexibility, the nucleolipid derivatives under test retain the nucleoside scaffold while being functionally modified by the introduction of lipophilic substituents. This structural concept combines the biological relevance of nucleosides with optimized physicochemical properties. For instance, 5-FUrd acts as a classical nucleoside analog, interfering with RNA metabolism and nucleotide biosynthesis [10], whereas TMZ is a small alkylating agent that can cross the blood–brain barrier due to its lipophilicity and stability [26]. However, the efficacy of TMZ is frequently limited by resistance development and metabolic reprogramming in glioblastoma cells, which contribute to tumor progression and therapy failure [27,28]. These limitations highlight the need for novel compounds with enhanced cellular uptake and sustained biological activity.

The analysis of lipophilicity revealed a clear dependence of membrane permeability on alkyl chain length. Derivatives with short alkyl chains (≤C_11_) exhibited high Δlog*P* values, indicating strong hydrogen-bonding interactions and reduced passive membrane permeability. Consequently, these compounds are likely unable to cross lipid bilayers, consistent with their limited biological activity. In contrast, derivatives with medium chain lengths (C_15_–C_17_) showed lower Δlog*P* values and a more favorable distribution between aqueous and lipophilic phases, suggesting increased membrane permeability and improved intracellular availability. These findings are consistent with established concepts describing the relationships among lipophilicity, hydrogen-bonding capacity, and membrane transport [29,30]. Furthermore, an optimal balance between hydrophilicity and lipophilicity represents a key determinant of drug-like properties and efficient cellular uptake [11,12,13,14,15,16,31,32].

Based on these physicochemical properties, the cytotoxicity data demonstrate a clear structure–activity relationship. Derivatives with medium-length alkyl chains (C_15_–C_17_) consistently exhibited the highest cytotoxic activity across all investigated tumor cell lines, whereas short-chain (≤C_11_) and long-chain (≥C_19_) derivatives showed markedly reduced effects. This bell-shaped relationship reflects an optimal hydrophilic–lipophilic balance required for efficient interaction with cellular membranes, adequate solubility, and effective engagement with intracellular targets [12,13,31]. In contrast, excessive lipophilicity in long-chain derivatives may lead to aggregation, membrane retention, or reduced bioavailability, thereby limiting their biological activity. Comparable trends have previously been described for lipophilized nucleosides and other amphiphilic drug candidates, supporting the general validity of this structure–activity relationship [12,13,14,15,16,31].

Importantly, the observed cytotoxicity cannot be solely attributed to ketalization. Short-chain ketal derivatives, particularly heptylidene-containing compounds (C_7_), exhibited little or no cytotoxic activity despite the presence of the ketal structure. In contrast, cytotoxicity increased progressively with increasing alkyl chain length, especially for undecylidene- to heptadecylidene-containing derivatives. This trend was consistently observed for both adenosine- and 1,N^6^-ethenoadenosine-derived nucleolipids, indicating that lipophilicity and membrane permeability, rather than ketalization itself, are the primary determinants of biological activity. The very low lipophilicity of the parent nucleosides adenosine (NS_5.0.0.0 (1)) and 1,N^6^-ethenoadenosine (εNS_5.0.0.0 (6)), reflected by negative log*P_OW_* values of −1.03 and −1.40, respectively, further supports this interpretation, especially also because Knies et al. [13] have already demonstrated a link between log*P_O_w* value and cytotoxicity, i.e., low or negative log*P_O_w* value was associated with a low cytotoxicity, whereas high log*P_O_w* value was associated with high cytotoxicity.

A key finding of this study is the concentration-dependent selectivity of the nucleolipid derivatives. The most active compounds (7c, 7d, 7h, 7i, 9) induced pronounced cytotoxicity in human/rat glioma and glioblastoma cells at concentrations of 6.25–12.5 µM. In contrast, a comparable reduction in cell viability in PMA-differentiated THP-1 macrophages was only observed at higher concentrations (≥25 µM). This indicates a degree of selectivity toward tumor cells, a crucial prerequisite for therapeutic application. The reduced sensitivity of macrophages at lower concentrations suggests that the observed cytotoxicity is not solely due to nonspecific membrane disruption but may also involve tumor-specific uptake mechanisms or differences in cellular metabolism.

This effect becomes even more apparent when compared to the reference compounds. Under our experimental conditions (1.56–50 µM, 48 h), TMZ did not induce a pronounced cytotoxic effect in either glioblastoma cell lines or PMA-differentiated THP-1 macrophages, while 5-FUrd exhibited cytostatic effects but overall lower activity compared to the nucleolipid derivatives. It should also be considered that many in vitro studies employ substantially higher TMZ concentrations, often exceeding 400 µM, to approximate in vivo exposure conditions [33]. However, such concentrations are far above those achievable in the central nervous system and are therefore of limited physiological relevance. Moreover, the use of high concentrations increases the risk of nonspecific effects, for example, due to the solvent DMSO [33]. In addition, TMZ-resistant cell populations require significantly higher drug concentrations to achieve comparable cytotoxicity, with reported increases of 4- to 40-fold in clonogenic cells [34]. This highlights the pronounced resistance problem and underscores the limitations of TMZ, particularly in long-term or repeated treatment settings. Against this background, compounds that are effective at substantially lower concentrations are of particular interest, as they may offer improved efficacy while reducing nonspecific toxicity.

In this context, the present results are particularly noteworthy, as the nucleolipid derivatives already exhibit strong cytotoxic effects at comparatively low concentrations and shorter incubation times. At the same time, the observation that cytotoxic effects in THP-1 macrophages occur only at higher concentrations suggests a partial therapeutic window. Nevertheless, the effects observed at higher concentrations suggest that off-target toxicity cannot be ruled out, underscoring the need for further structural optimization to improve tumor selectivity and expand the therapeutic window [23,24].

In addition to lipophilicity and alkyl chain length, modification of the nucleobase represents another important determinant of biological activity. 1,N^6^-ethenoadenosine-derived nucleolipids generally exhibited higher cytotoxic activity than their corresponding adenosine analogs. This effect may be attributed to the altered electronic structure, increased rigidity, and modified hydrogen-bonding properties of the etheno-modified purine system, which may enhance interactions with intracellular targets or influence cellular uptake mechanisms [18]. Previous studies have demonstrated that structural modification of nucleoside scaffolds can significantly enhance cytotoxic and antitumor activity [12,13,14,15,16,35], supporting the concept that lipophilicity and nucleobase modification act synergistically as key determinants of biological activity.

Overall, this study demonstrates that lipophilicity and alkyl chain length are the primary determinants of cytotoxic activity in nucleolipid derivatives. Medium-chain derivatives provide an optimal balance between membrane permeability, intracellular accumulation, and biological activity, making them particularly promising candidates for further development. Future studies should focus on improving tumor selectivity, elucidating molecular mechanisms of action, and evaluating in vivo efficacy, pharmacokinetics, and potential toxicity profiles in more complex biological systems.

## 4. Materials and Methods

### 4.1. General Experimental Procedures

All reagents and solvents were purchased from commercial suppliers (e.g., Sigma-Aldrich, St. Louis, MO, USA, Merck, Darmstadt, Germany, TCI Europe, Zwijndrecht, Belgium) and used as received without further purification unless otherwise stated.

Thin-layer chromatography (TLC) was performed on silica gel 60 F254 aluminum plates (Macherey-Nagel, Düren, Germany). Column chromatography was carried out using silica gel 60 (0.063–0.200 mm, J.T. Baker, Griesheim, Germany).

All synthesized compounds were characterized using standard analytical techniques. Nuclear magnetic resonance (NMR) spectra (^1^H, ^13^C, DEPT-135) were recorded on an AMX-500 spectrometer (Bruker, Rheinstetten, Germany) at 500 MHz (^1^H) and 126 MHz (^13^C). Chemical shifts (δ) are reported in ppm relative to [d_6_] DMSO (2.50 ppm for ^1^H, 39.50 ppm for ^13^C) using tetramethylsilane as internal standard. Coupling constants (J) are given in Hz.

High-resolution electrospray ionization mass spectrometry (HR-ESI-MS) measurements were performed on an Esquire HCT instrument (Bruker Daltonics, Leipzig, Germany).

UV/Vis spectra were recorded on a Cary 50 spectrophotometer (Varian, Darmstadt, Germany), and fluorescence spectra were obtained using an F-4500 spectrometer (Hitachi High Technologies, Tokyo, Japan).

Elemental analyses (C, H, N) were carried out on a VarioMICRO analyzer (Elementar, Hanau, Germany).

Cell viability measurements were performed using a SUNRISE™ microplate reader (Tecan Group Ltd., Männedorf, Switzerland).

### 4.2. Synthesis and Characterisation of Nucleolipid Derivatives

A series of nucleolipid derivatives based on adenosine and 1,N^6^-ethenoadenosine, featuring lipophilic groups at the O-2′,3′-position of the ribose, were synthesized. The approach involved ketalization of adenosine with aliphatic ketones of varying chain lengths, yielding both symmetric and asymmetric O-2′,3′-ketals. Additionally, cyclic and bulky groups—including pentanyliden, cycloalkyl, and adamantyliden structures—as well as a levulinic acid ethyl ester residue, were incorporated to systematically adjust lipophilicity.

Subsequent conversion into the corresponding 1,N^6^-ethenoadenosine derivatives was achieved by reaction with chloroacetaldehyde according to established procedures. A total of 20 novel nucleolipid derivatives were synthesized in this study, complemented by structurally related compounds previously reported by our group [13,14,15,16,17], which were included for comparative structure–activity relationship analysis.

All compounds were characterized by NMR spectroscopy, mass spectrometry, elemental analysis, UV/Vis, and fluorescence spectroscopy. Detailed synthetic procedures and full analytical data are provided in the Supplementary Materials.

### 4.3. Determination of Lipophilicity (logP)

The lipophilicity of the synthesized compounds was determined experimentally using biphasic partition systems. Log*P_OW_* values were obtained using a 1-octanol/water system, while log*P_ChW_* values were determined using a cyclohexane/water system to assess hydrogen-bonding contributions.

Samples of each compound (2 mg) were distributed between 1-octanol (50 mL) and water (50 mL) or cyclohexane (50 mL) and water (50 mL) and stirred for 1 h at room temperature (20–22 °C) to reach equilibrium. After phase separation, aliquots of each phase were transferred into quartz cuvettes and analyzed by UV/Vis spectroscopy.

Partition coefficients were calculated from the ratio of absorbance values at the wavelength of maximum absorption (λmax) in both phases and expressed as log10POW and log10PChW. The difference between both values (Δlog*P* = log*P_OW_* − log*P_ChW_*) was used as an indicator of hydrogen-bonding capacity and membrane permeability.

1-Octanol and cyclohexane were obtained from Sigma-Aldrich (Munich, Germany).

### 4.4. Cell Culture

In vitro experiments were performed using rat malignant neuroectodermal BT4Ca glioma cells (kindly provided by Dr. N. John, Hannover Medical School, Hannover, Germany), human astrocytoma/oligodendroglioma GOS-3 cells (ACC 408; DSMZ GmbH, Braunschweig, Germany), human glioblastoma U87 MG cells (HTB-14; ATCC, Manassas, VA, USA), and human glioblastoma U251 MG cells (300385; CLS Cell Lines Service GmbH, Eppelheim, Germany). In addition, the human monocytic leukemia cell line THP-1 (ACC 16; DSMZ GmbH, Braunschweig, Germany) was used.

BT4Ca and GOS-3 cells were cultured in high-glucose DMEM medium supplemented with sodium pyruvate (Capricorn Scientific GmbH, Ebsdorfergrund, Germany), whereas THP-1 cells were cultured in RPMI 1640 medium (Capricorn Scientific GmbH). All media were supplemented with 10% heat-inactivated fetal bovine serum (FBS), 100 U/mL penicillin, and 0.1 mg/mL streptomycin. U87 MG and U251 MG cells were additionally supplemented with non-essential amino acids (NEAA; Capricorn Scientific GmbH).

All cell lines were maintained at 37 °C in a humidified atmosphere containing 5% CO_2_.

### 4.5. THP-1 Differentiation

For differentiation into macrophage-like cells, THP-1 monocytes were seeded in 96-well plates at a density of 3 × 10^4^ cells per well and treated with phorbol 12-myristate 13-acetate (PMA; 100 ng/mL; Sigma-Aldrich, Munich, Germany) for 72 h. Following differentiation, cells were used for cytotoxicity experiments.

### 4.6. Cell Viability Assay

To evaluate cytotoxic and cytostatic effects, cells were seeded in 96-well plates at the following densities: 5 × 10^3^ (BT4Ca), 1 × 10^4^ (GOS-3), 5 × 10^3^ (U87 MG), and 7.5 × 10^3^ (U251 MG) cells per well. After 24 h incubation, cells were treated with test compounds at concentrations of 1.25, 3.12, 6.25, 12.5, 25, and 50 µM.

The following compounds were investigated: adenosine-derived nucleolipids NL_5.4.0.0 (2a), NL_5.5.0.0 (2b), NL_5.7.0.0 (2c), NL_5.9.0.0 (2d), NL_5.3.0.0 (2e), NL_5.4_2_.0.0 (2f), NL_5.5_2_.0.0 (2g), NL_5.7_2_.0.0 (2h), NL_5.8_3_.0.0 (2i), NL_5.3_2_.0.0 (2j), NL_5.1.0.0 (3), NL_5.cycl7.0.0 (4), and NL_5.cycl8.0.0 (5), as well as ethenoadenosine derivatives εNL_5.4.0.0 (7a), εNL_5.5.0.0 (7b), εNL_5.7.0.0 (7c), εNL_5.9.0.0 (7d), εNL_5.3.0.0 (7e), εNL_5.4_2_.0.0 (7f), εNL_5.5_2_.0.0 (7g), εNL_5.7_2_.0.0 (7h), εNL_5.8_3_.0.0 (7i), εNL_5.3_2_.0.0 (7j), εNL_5.1.0.0 (8), εNL_5.cycl7.0.0 (9), and εNL_5.cycl8.0.0 (10).

5-Fluorouridine (5-FUrd; TCI Europe, Zwijndrecht, Belgium) and temozolomide (TMZ; Sigma-Aldrich, Munich, Germany) were used as reference compounds.

After 48 h of incubation, cell viability was assessed using PrestoBlue™ reagent (Invitrogen, Darmstadt, Germany), added at a final concentration of 10%. After incubation (30 min for BT4Ca, 45 min for GOS-3, and 90 min for THP-1), absorbance was measured at 570 nm and 600 nm using a microplate reader. Cell viability was calculated as a percentage relative to untreated control cells (set to 100%). To exclude solvent effects, cells were additionally treated with medium containing 0.5% DMSO as a negative control. All experiments were performed in four independent replicates, each conducted in quadruplicate.

Statistical analysis was performed using Student’s *t*-test, and significance was defined as * *p* ≤ 0.05, ** *p* ≤ 0.01, *** *p* ≤ 0.001 vs. DMSO; ^#^ *p* ≤ 0.05, ^##^ *p* ≤ 0.01, ^###^ *p* ≤ 0.001 vs. 5-FUrd; ° *p* ≤ 0.05, °° *p* ≤ 0.01, °°° *p* ≤ 0.001 vs. TMZ.

## 5. Conclusions

This study involved synthesizing a series of lipophilized adenosine and 1,N6-ethenoadenosine nucleolipid derivatives, which were systematically tested in human/rat glioma and glioblastoma cell models. The results show that lipophilicity and alkyl chain length are crucial factors influencing the biological activity of these compounds.

Our results suggest that an optimal hydrophilic–lipophilic balance, particularly achieved by medium-length alkyl chains (C_15_–C_17_), is essential for effective cytotoxic activity. Deviations from this balance, either toward insufficient or excessive lipophilicity, appear to limit biological efficacy, highlighting the importance of fine-tuning physicochemical properties in nucleoside-based drug design.

Furthermore, the enhanced activity observed for 1,N^6^-ethenoadenosine derivatives indicates that nucleobase modification represents an additional strategy to improve cytotoxic potency. When combined with lipophilization, this approach may provide a rational framework for developing more effective nucleoside-derived therapeutics.

Importantly, the investigated nucleolipid derivatives demonstrated higher cytotoxic activity than established reference compounds such as 5-FUrd and TMZ, while showing comparatively lower effects in PMA-differentiated THP-1 macrophages. These findings suggest a favorable balance between antitumor efficacy and cellular selectivity, which is a critical parameter for further therapeutic development.

Overall, this research emphasizes the potential of lipophilized nucleoside analogs as a promising group of compounds for the treatment of malignant brain tumors. Future research should aim to clarify the underlying molecular mechanisms, enhance tumor selectivity, and assess in vivo efficacy and pharmacokinetic properties to enable clinical use of these compounds.

## 6. Patents

The compounds and synthetic strategies described in this study are related to an European patent entitled “Novel adenosine and 1,N^6^-ethenoadenosine derivatives for cancer treatment” (EP 4 342 905 A1).

The patent covers adenosine and 1,N^6^-ethenoadenosine derivatives bearing lipophilic substituents at the O-2′,3′-positions, including symmetric and asymmetric ketal structures with varying alkyl chain lengths, as well as their potential application in cancer therapy.

## Figures and Tables

**Table 1:** Nomenclature and coding system of synthesized adenosine and 1,N^6^-ethenoadenosine nucleolipid derivatives.

Compound	NS/NL	Compound	NS/NL
**Adenosine (1)**	NS_5.0.0.0	**1,N^6^-ethenodenosine (6)**	ɛNS_5.0.0.0 [18]
**2a**	NL_5.4.0.0 [14]	**7a**	ɛNL_5.4.0.0
**2b**	NL_5.5.0.0 [14]	**7b**	ɛNL_5.5.0.0
**2c**	NL_5.7.0.0	**7c**	ɛNL_5.7.0.0
**2d**	NL_5.9.0.0	**7d**	ɛNL_5.9.0.0
**2e**	NL_5.3.0.0 [13]	**7e**	ɛNL_5.3.0.0
**2f**	NL_5.4_2_.0.0	**7f**	ɛNL_5.4_2_.0.0
**2g**	NL_5.5_2_.0.0	**7g**	ɛNL_5.5_2_.0.0
**2h**	NL_5.7_2_.0.0	**7h**	ɛNL_5.7_2_.0.0
**2i**	NL_5.8_3_.0.0	**7i**	ɛNL_5.8_3_.0.0
**2j**	NL_5.3_2_.0.0	**7j**	ɛNL_5.3_2_.0.0
**3**	NL_5.1.0.0 [17]	**8**	ɛNL_5.1.0.0
**4**	NL_5.cycl7.0.0 [14]	**9**	ɛNL_5.cycl7.0.0
**5**	NL_5.cycl8.0.0	**10**	ɛNL_5.cycl8.0.0

**Table 2:** Experimentally determined (log*P_OW_*_)_, (log*P_ChW_*), Δlog*P*, and calculated log_(cbrain/cblood_) values of adenosine and ethenoadenosine nucleolipid derivatives.

NS/NL	log*P*_OW_	log*P_ChW_*	∆log*P*	log$\left(\frac{C_{brain}}{C_{blood}}\right)$
NS_5.0.0.0 (1)	−1.03	−3.88	2.85	−0.49
NL_5.4.0.0 (2a)	2.27	−0.79	3.05	−0.61
NL_5.5.0.0 (2b)	−0.04	−0.46	0.42	0.98
NL_5.7.0.0 (2c)	2.82	2.22	0.60	0.87
NL_5.9.0.0 (2d)	2.86	2.48	0.83	1.00
NL_5.3.0.0 (2e)	3.58	1.92	1.66	0,23
NL_5.4_2_.0.0 (2f)	2.82	−0.72	3.54	−0.91
NL_5.5_2_.0.0 (2g)	2.35	1.97	0.38	1.00
NL_5.7_2_.0.0 (2h)	2.83	2.24	0.59	0.87
NL_5.8_3_.0.0 (2i)	3.60	2.45	1.15	0.53
NL_5.3_2_.0.0 (2j)	4.08	2.39	1.68	0.21
NL_5.1.0.0 (3)	0.58	−2.58	3.15	−0.68
NL_5.cycl7.0.0 (4)	1.52	0.40	1.12	0.55
NL_5.cycl8.0.0 (5)	0.44	−1.65	2.08	−0.03
ɛNS_5.0.0.0 (6)	−1.40	−1.73	0.33	1.03
ɛNL_5.4.0.0 (7a)	2.06	−1.48	3.54	−0.91
ɛNL_5.5.0.0 (7b)	1.99	0.54	1.45	0.36
ɛNL_5.7.0.0 (7c)	2.40	3.23	−0.83	1.73
ɛNL_5.9.0.0 (7d)	2.57	1.72	0.85	0.72
ɛNL_5.3.0.0 (7e)	3.82	1.95	1.87	0.10
ɛNL_5.4_2_.0.0 (7f)	1.95	−1.97	3.92	−1.14
ɛNL_5.5_2_.0.0 (7g)	3.09	0.81	2.29	−0.15
ɛNL_5.7_2_.0.0 (7h)	2.75	1.95	0.80	0.75
ɛNL_5.8_3_.0.0 (7i)	2.29	2.29	0.01	1.23
ɛNL_5.3_2_.0.0 (7j)	3.82	2.01	1.81	0.14
ɛNL_5.1.0.0 (8)	0.53	−2.98	3.51	−0.89
ɛNL_5.cycl7.0.0 (9)	1.67	1.28	0.39	0.99
ɛNL_5.cycl8.0.0 (10)	1.63	−0.95	2.58	−0.33

## Data Availability

The data presented in this study are available from the corresponding author upon reasonable request.

## Supplementary Materials

The following supporting information can be downloaded at: https://www.mdpi.com/article/10.3390/ijms27114922/s1.

## Abbreviations

The following abbreviations are used in this manuscript:

5-FUrd	5-Fluorouridine
TMZ	Temozolomide
BBB	Blood–brain barrier
NS	Nucleoside
NL	Nucleollipid
PMA	Phorbol 12-myristate 13-acetate
FBS	Fetal bovine serum
DMEM	Dulbecco’s Modified Eagle Medium
RPMI	Roswell Park Memorial Institute medium
NEAA	Non-essential amino acids
log*P_OW_*	Octanol/water partition coefficient
log*P_ChW_*	Cyclohexane/water partition coefficient
Δlog*P*	Difference between log*P_OW_* and log*P_ChW_*
SEM	Standard error of the mean
DMSO	Dimethyl sulfoxide
