# Supplementary material for: Adenosine and 1,N6-Ethenoadenosine-Derived Nucleolipids: Synthesis, Lipophilicity (logP), and Cytotoxic Activity Compared to Conventional Cytostatics in Glioma and Glioblastoma Cell Lines
Source: Int J Mol Sci. 2026 May 29;27(11):4922. doi: 10.3390/ijms27114922 (PMC13256577; doi:10.3390/ijms27114922)
Supplement: Supplementary file 1 [file ijms-27-04922-s001.zip › ijms-4317772-supplementary.pdf]

Supplementary Material

Adenosine and 1,N^6-Ethenoadenosine-Derived Nucleolipids: Synthesis, Lipophilicity (log*P*), and Cytotoxic Activity Compared to Conventional Cytostatics in Glioma and Glioblastoma Cell Lines

Mona Lünswilken [1], Eugenia Bender-Arnst [2], Fatima Barakat [2], Eugen Leinweber [2], Uwe Beginn [2], Gabriel A. Bonaterra [§,1] and Ralf Kinscherf [§,1]

[1] Institute for Anatomy and Cell Biology, Department of Medical Cell Biology, Philipps-University of Marburg, Robert-Koch-Str. 8, D-35032 Marburg luenswil@staff.uni-marburg.de
[2] Organic Chemistry I – Bioorganic Chemistry, Institute of Chemistry of New Materials, University of Osnabrück, Barbarastr. 7, D-49076 Osnabrück
[§] Senior Authors

1. General Experimental Procedures

All reagents and solvents were purchased from commercial suppliers (e.g., Sigma-Aldrich, St. Louis, MO, USA, Merck, Darmstadt, Germany, TCI Europe, Zwijndrecht, Belgium) and used as received without further purification unless otherwise stated. Thin-layer chromatography (TLC) was performed on silica gel 60 F254 aluminum plates (Macherey-Nagel, Düren, Germany). Column chromatography was carried out using silica gel 60 (0.063–0.200 mm, J.T. Baker, Griesheim, Germany). All synthesized compounds were characterized using standard analytical techniques. Nuclear magnetic resonance (NMR) spectra (^{1}H, ^{13}C, DEPT-135) were recorded on an AMX-500 spectrometer (Bruker, Rheinstetten, Germany) at 500 MHz (^{1}H) and 126 MHz (^{13}C). Chemical shifts (δ) are reported in ppm relative to [d$_6$] DMSO (2.50 ppm for ^{1}H, 39.50 ppm for ^{13}C) using tetramethylsilane as internal standard. Coupling constants (*J*) are given in Hz. High-resolution electrospray ionization mass spectrometry (HR-ESI-MS) measurements were performed on an Esquire HCT instrument (Bruker Daltonics, Leipzig, Germany). UV/Vis spectra were recorded on a Cary 50 spectrophotometer (Varian, Darmstadt, Germany), and fluorescence spectra were obtained using an F-4500 spectrometer (Hi-tachi High Technologies, Tokyo, Japan). Elemental analyses (C, H, N) were carried out on a VarioMICRO analyzer (Elementar, Hanau, Germany). Cell viability measurements were performed using a SUNRISE™ microplate reader (Tecan Group Ltd., Männedorf, Switzerland).

2. Synthesis of Lipophilized Adenosine and 1,N⁶ Ethenoadenosine Nucleolipids

2.1. Symmetrically O-2′,3′-Ketalized Nucleolipids

2.1.1. O-2′,3′-Nucleolipids of Adenosine

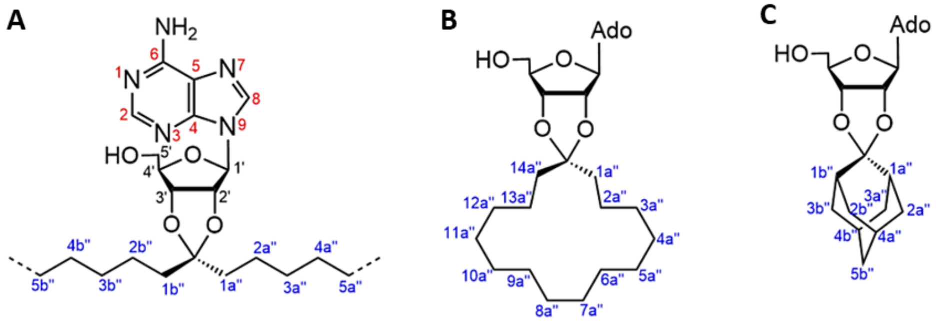

Supplementary Figure S1. Schematic illustration of the numbering of symmetrical O-2′,3′-ketalized adenosine (Ado) derivatives. (**A**) general numbering scheme for all linear alkyl chains. (**B**) Numbering of the pentadecanyliden cyclic ketal. (**C**) Numbering of the adamantanone-derived ketal.

((3aR,4R,6R,6aR)-6-(6-amino-9H-purin-9-yl)-2,2-dipropyltetrahydrofuro[3,4-d][1,3]dioxol-4-yl)methanol **(2a, NL_5.4.0.0)** *(adapted from Hammerbacher et al., 2018)*

Anhydrous Adenosine (1, NS_5.0.0.0; 1.0 g; 3.74 mmol) was dissolved in 8 mL of dry dimethylformamide (DMF) in the absence of amines. Heptan-4-one (0.55 mL; 4.0 mmol), triethyl orthoformate (0.662 mL; 4.0 mmol), and 5 mL of a 4 M HCl solution in 1,4-dioxane were subsequently added. The reaction mixture was stirred at room temperature for 24 hours. Upon completion, the mixture was transferred to 80 mL of CH_2Cl_2 and 10 mL of saturated sodium hydrogen carbonate solution to quench the reaction. The aqueous phase was extracted three times with 12.5 mL portions of CH_2Cl_2. The combined organic layers were dried over $MgSO_4$ for 1 hour, filtered, and concentrated in vacuo using a rotary evaporator. To remove residual DMF, the crude product was subjected to repeated evaporations with CH_2Cl_2. The resulting material was dried overnight under high vacuum at room temperature, then crystallized from CH_2Cl_2 at 4 °C, followed by an additional drying step under high vacuum. This procedure ensured the removal of all traces of DMF. The final solvent-free product was obtained as a white solid (1.135 g, 83 % yield).

R_f = 0.75 (SiO_2 60, CH_2Cl_2/MeOH 9:1; v/v).

UV/Vis (MeOH): λ_{max}: 260 nm (ε=14366.9 L·mol^{-1}·cm^{-1}).

logP_{OW} (ALOGPS 3.01): 2.32 ± 0.74.

^{1}H NMR (500 MHz, DMSO-d$_6$): δ 8.34 (s, 1H, H-C(2)), 8.16 (s, 1H, H-C(8)), 7.28 (s, 2H, NH_2), 6.13 (d, *J* = 3.0 Hz, 1H, H-C(1′)), 5.38 (dd, *J* = 2.5, 6.5 Hz, 1H, H-C(2′)), 5.13 (t, *J* = 5.0 Hz, 1H, HO-C(5′)), 4.97 (dd, *J* = 2.5, 6.5 Hz, 1H, H-C(3′)), 4.20–4.18 (m, 1H, H-C(4′)), 3.55–3.46 (m, 2H, H_2-C(5′)), 1.73–1.70 (m, 2H, H_2-C(1a″)), 1.57–1.54 (m, 2H, H_2-C(1b″)), 1.47–1.45 (m, 2H, H_2-C(2a″)), 1.31–1.27 (m, 2H, H_2-C(2b″)), 0.95 (t, *J* = 7.5 Hz, 3H, H_3-C(3a″)), 0.87 (t, *J* = 7.5 Hz, 3H, H_3-C(3b″)).

^{13}C NMR (126 MHz, DMSO-d$_6$): δ 156.05 (C(6)), 152.57 (C(2)), 148.80 (C(4)), 139.67 (C(8)), 118.99 (C(5)), 116.55 (C(acetal)), 89.49 (C(1′)), 86.83 (C(4′)), 83.54 (C(2′)), 81.53 (C(3′)), 61.52 (C(5′)), 16.96 (C(1a″), C(1b″)), 16.31 (C(2a″), C(2b″)), 14.09 (C(3a″)), 14.07 (C(3b″)).

ESI – MS: m/z 364.04 [M+H]$^+$ (calcd for $C_{17}H_{25}N_5O_4$: 363.41).

Elemental analysis calcd (%) $C_{17}H_{25}N_5O_4$: C 56.16, H 6.84, N 19.53; found: C 56.18, H 6.93, N 19.27.

((3aR,4R,6R,6aR)-6-(6-amino-9H-purin-9-yl)-2,2-dipentyltetrahydrofuro[3,4-d][1,3]dioxol-4-yl)methanol **(2b, NL_5.5.0.0)** *(adapted from Hammerbacher et al., 2018)*

Adenosine (1**,** NS_5.0.0.0; 1.0 g; 3.74 mmol) was dissolved in 15 mL of dry DMF, in the absence of amines. Undecan-6-one (0.819 mL; 4.0 mmol), triethyl orthoformate (0.662 mL), and 7.5 mL of a 4 M HCl solution

in 1,4-dioxane were then added. The reaction mixture was stirred at room temperature for 24 hours. Upon completion, the mixture was partitioned between 40 mL of CH_2Cl_2 and a saturated sodium hydrogen carbonate solution. The aqueous layer was extracted three times with 12.5 mL portions of CH_2Cl_2. The combined organic extracts were washed with 100 mL of water and dried over magnesium sulfate for one hour. After filtration, the solution was concentrated under reduced pressure using a rotary evaporator and subjected to several rounds of evaporation with CH_2Cl_2 to thoroughly remove residual DMF. The crude product was dried overnight under high vacuum at room temperature. Purification was achieved by column chromatography on silica gel (SiO_2 60, column: 6.5 cm × 7 cm; eluent: CH_2Cl_2/MeOH 9:1, v/v). The final product, isolated as a solvent-free white solid under high vacuum, was obtained in a yield of 0.9862 g (63 %).

R_f = 0.81 (SiO_2 60, CH_2Cl_2/MeOH 9:1; v/v).

UV/Vis (MeOH): λ_{max}: 260 nm (ε=11951.3 L·mol^{-1}·cm^{-1}).

logP_{OW} (ALOGPS 3.01): 3.60 ± 0.74.

^{1}H NMR (500 MHz, DMSO-d$_6$): δ 8.34 (s, 1H, H-C(2)), 8.15 (s, 1H, H-C(8)), 7.27 (s, 2H, NH_2), 6.13 (d, *J* = 2.5 Hz, 1H, H-C(1′)), 5.36 (dd, *J* = 2.5, 6.5 Hz, 1H, H-C(2′)), 5.12 (t, *J* = 5.0 Hz, 1H, HO-C(5′)), 4.96 (dd, *J* = 2.5, 6.5 Hz, 1H, H-C(3′)), 4.19–4.18 (m, 1H, H-C(4′)), 3.52–3.48 (m, 2H, H_2-C(5′)), 1.73–1.71 (m, 2H, H_2-C(1a″)), 1.56–1.54 (m, 2H, H_2-C(1b″)), 1.43–1.41 (m, 2H, H_2-C(2a″)), 1.31–1.24 (m, 10H, H_2-C(2b″), H_2-C(3a″), H_2-C(3b″), H_2-C(4a″), H_2-C(4b″)), 0.90–0.87 (m, 6H, H_3-C(5a″), H_3-C(5b″)).

^{13}C NMR (126 MHz, DMSO-d$_6$): δ 156.09 (C(6)), 152.63 (C(2)), 148.87 (C(4)), 139.72 (C(8)), 119.01 (C(5)), 116.75 (C(acetal)), 89.51 (C(1′)), 86.92 (C(4′)), 83.63 (C(2′)), 81.56 (C(3′)), 61.58 (C(5′)), 36.39 (C(1a″)), 36.26 (C(1b″)), 31.36 (C(3a″)), 31.34 (C(3b″)), 23.28 (C(2a″)), 22.61 (C(2b″)), 21.98 (C(4a″)), 21.94 (C(4b″)), 13.84 (C(5a″)), 13.79 (C(5b″)).

ESI – MS: m/z 420.11 [M+H]$^+$ (calcd for $C_{21}H_{33}N_5O_4$: 419.52).

Elemental analysis calcd (%) $C_{21}H_{33}N_5O_4$: C 59.79, H 7.65, N 16.4; found: C 60.12, H 7.93, N 16.69.

((3aR,4R,6R,6aR)-6-(6-amino-9H-purin-9-yl)-2,2-diheptyltetrahydrofuro[3,4-d][1,3]dioxol-4-yl)methanol **(2c, NL_5.7.0.0)**

Anhydrous adenosine (1, NS_5.0.0.0; 1.0 g; 3.74 mmol) was dissolved in 15 mL of dry, amine-free DMF. Pentadecan-8-one (0.906 g; 4.0 mmol), triethyl orthoformate (0.662 mL), and 7.5 mL of 4 M HCl in 1,4-dioxane were then added. The reaction mixture was stirred at room temperature for 24 hours. After completion, the mixture was partitioned between 60 mL of CH_2Cl_2 and a saturated aqueous $NaHCO_3$ solution. The aqueous layer was extracted three times with 12.5 mL portions of CH_2Cl_2. The combined organic layers were washed with 120 mL of water and dried over magnesium sulfate. The solution was filtered, and the solvent was removed under reduced pressure using a rotary evaporator. Residual DMF was eliminated by repeated co-evaporation with CH_2Cl_2. The resulting oily residue was dried overnight under high vacuum at room temperature. Purification was performed by column chromatography on silica gel (SiO_2 60, column: 6.5 cm × 7.5 cm; CH_2Cl_2/MeOH 9:1, v/v). Further drying under high vacuum overnight afforded the product as a white solid (0.9562 g, 54 % yield).

R_f = 0.82 (SiO_2 60, CH_2Cl_2/MeOH 9:1; v/v).

UV/Vis (MeOH): λ_{max}: 259 nm (ε=14648.0 L·mol^{-1}·cm^{-1}).

logP_{OW} (ALOGPS 3.01): 4.24 ± 0.74.

^{1}H NMR (500 MHz, DMSO-d$_6$): δ 8.34 (s, 1H, H-C(2)), 8.15 (s, 1H, H-C(8)), 7.28 (s, 2H, NH_2), 6.13 (d, *J* = 3.0 Hz, 1H, H-C(1′)), 5.36 (dd, *J* = 3.0, 6.5 Hz, 1H, H-C(2′)), 5.10 (t, *J* = 5.5 Hz, 1H, HO-C(5′)), 4.96 (dd, *J* = 2.5, 6.0 Hz, 1H, H-C(3′)), 4.20–4.18 (m, 1H, H-C(4′)), 3.56–3.46 (m, 2H, H_2-C(5′)), 1.74–1.71 (m, 2H, H_2-C(1a″)), 1.56–1.55 (m, 2H, H_2-C(1b″)), 1.43–1.42 (m, 2H, H_2-C(2a″)), 1.31–1.24 (m, 18H, H_2-C(2b″), H2-C(3a″), H_2-C(3b″), H_2-C(4a″–6a″), H_2-C(4b″–6b″)), 0.88–0.83 (m, 6H, H_3-C(7a″), H_3-C(7b″)).

^{13}C NMR (126 MHz, DMSO-d$_6$): δ 156.04 (C(6)), 152.53 (C(2)), 148.81 (C(4)), 139.61 (C(8)), 118.98 (C(5)), 116.65 (C(acetal)), 89.41 (C(1′)), 86.83 (C(4′)), 83.54 (C(2′)), 81.48 (C(3′)), 61.52 (C(5′)), 36.38 (C(1a″)), 36.20 (C(1b″)), 31.13 (C(2a″)), 31.08 (C(2b″)), 28.52 (C(3a″)), 28.45 (C(3b″)), 21.98 (C(4a″–6a″)), 21.94 (C(4b″–6b″)), 13.82 (C(7a″)), 13.78 (C(7b″)).

ESI – MS: m/z 476.26 $[M+H]^+$ (calcd for $C_{25}H_{41}N_5O_4$: 475.62).
Elemental analysis calcd (%) $C_{25}H_{41}N_5O_4 \cdot 0.15\ H_2O$: C 62.95, H 8.89, N 14.36; found: C 62.78, H 8.70, N 14.64.

((3aR,4R,6R,6aR)-6-(6-amino-9H-purin-9-yl)-2,2-dioctyltetrahydrofuro[3,4-d][1,3]-dioxol-4-yl)methanol ***(2d, NL_5.9.0.0)***

Anhydrous adenosine (1, NS_5.0.0.0; 1 g, 3.74 mmol) was dissolved in 15 mL of dry, amine-free DMF. Heptadecan-9-one (1.018 g, 4 mmol), triethyl orthoformate (0.662 mL, 4 mmol), and 7.5 mL of 4 M HCl in 1,4-dioxane were then added. The reaction mixture was stirred at room temperature for 24 hours. Upon completion, the mixture was partitioned between 80 mL of CH_2Cl_2 and a saturated aqueous sodium hydrogen carbonate solution. The aqueous layer was extracted three times with 12 mL portions of CH_2Cl_2. The combined organic phases were washed three times with 50 mL of water and dried over $MgSO_4$. After filtration, the organic solvents were removed in vacuo using a rotary evaporator, and any residual DMF was eliminated by repeated co-evaporation with CH_2Cl_2. The resulting crude product was dried overnight under high vacuum at room temperature. Purification was accomplished by column chromatography on silica gel (SiO_2 60, column: 6.5 cm × 11.5 cm; CH_2Cl_2/MeOH 9:1, v/v). The isolated, solvent-free product was obtained as a white solid (1.2997 g, 69 % yield).

R_f = 0.83 (SiO_2 60, CH_2Cl_2/MeOH 9:1; v/v).
UV/Vis (MeOH): λ_{max}: 260 nm (ε=13704.7 $L \cdot mol^{-1} \cdot cm^{-1}$).
logP_{OW} (ALOGPS 3.01): 4. 68 ± 0.74.
^1H NMR (500 MHz, DMSO-d_6): δ 8.33 (s, 1H, H-C(2)), 8.15 (s, 1H, H-C(8)), 7.28 (s, 2H, NH_2), 6.13 (d, *J* = 2.5 Hz, 1H, H-C(1′)), 5.36 (dd, *J* = 2.5, 6.0 Hz, 1H, H-C(2′)), 5.10 (t, *J* = 5.5 Hz, 1H, HO-C(5′)), 4.96 (dd, *J* = 2.5, 6.0 Hz, 1H, H-C(3′)), 4.20–4.18 (m, 1H, H-C(4′)), 3.55–3.47 (m, 2H, H_2-C(5′)), 1.74–1.71 (m, 2H, H_2-C(1a″)), 1.56–1.54 (m, 2H, H_2-C(1b″)), 1.43–1.42 (m, 2H, H_2-C(2a″)), 1.30–1.24 (m, 22H, H_2-C(2b″)–H_2-C(7b″), H_2-C(3a″)–H_2-C(7a″)), 0.87–0.82 (m, 6H, H_3-C(8a″), H_3-C(8b″)).
^{13}C NMR (126 MHz, DMSO-d_6): δ 156.03 (C(6)), 152.49 (C(2)), 148.78 (C(4)), 139.58 (C(8)), 118.97 (C(5)), 116.63 (C(acetal)), 89.41 (C(1′)), 86.81 (C(4′)), 83.53 (C(2′)), 81.45 (C(3′)), 61.50 (C(5′)), 36.36 (C(1a″)), 36.14 (C(1b″)), 31.16 (C(2a″)), 31.12 (C(2b″)), 30.51 (C(3a″)), 29.05 (C(3b″)), 28.79–28.51 (4 × C, C(4a″)–C(7a″)), 23.50–21.92 (4 × C, C(4b″)–C(7b″)), 13.78 (C(8a″)), 13.75 (C(8b″)).
ESI – MS: m/z 504.35 $[M+H]^+$ (calcd for $C_{27}H_{45}N_5O_4$: 503.68).
Elemental analysis calcd (%) for $C_{27}H_{45}N_5O_4 \cdot 0.05\ H_2O \cdot 0.15$ Heptadecan-9-one: C 65.25, H 9.102, N 12.80; found: C 64.38, H 9.01, N 13.90.

((3aR,4R,6R,6aR)-6-(6-amino-9H-purin-9-yl)-2,2-dinonyltetrahydrofuro[3,4-d][1,3]-dioxol-4-yl)methanol **(2e, NL_5.3.0.0)** *(adapted from Knies et al., 2016b)*

A solution of anhydrous adenosine (1, NS_5.0.0.0; 0.5 g, 1.87 mmol) in 12 mL of dry, amine-free DMF was prepared. Nonadecan-10-one (0.5675 g, 2 mmol), triethyl orthoformate (0.35 mL, 5.61 mmol), and 3.0 mL of 4 M HCl in 1,4-dioxane were sequentially added to the solution. The reaction was stirred at room temperature for 24 hours. Following completion, the mixture was distributed between 175 mL of CH_2Cl_2 and 50 mL of saturated aqueous $NaHCO_3$. The aqueous layer was extracted three times with 12.5 mL portions of CH_2Cl_2. The combined organic extracts were washed with 120 mL of water and dried over magnesium sulfate for one hour. The solution was then filtered and concentrated under reduced pressure using a rotary evaporator. To ensure complete removal of DMF, the residue was co-evaporated several times with CH_2Cl_2. The crude product was dried overnight under high vacuum at room temperature. Final purification was achieved by column chromatography on silica gel (SiO_2 60, column: 6.5 cm × 7.5 cm; eluent: CH_2Cl_2/MeOH 9:1, v/v). The pure, solvent-free product was isolated as a white solid (0.3983 g, 40 % yield).

R_f = 0.84 (SiO_2 60, CH_2Cl_2/MeOH 9:1; v/v).
UV/Vis (MeOH): λ_{max}: 260 nm (ε=13704.7 $L \cdot mol^{-1} \cdot cm^{-1}$).
logP_{OW} (ALOGPS 3.01): 5.13 ± 0.74.

^{1}H NMR (500 MHz, DMSO-d_6): δ 8.33 (s, 1H, H-C(2)), 8.14 (s, 1H, H-C(8)), 7.29 (s, 2H, NH_2), 6.13 (d, *J* = 2.5 Hz, 1H, H-C(1′)), 5.34 (dd, *J* = 2.5, 6.0 Hz, 1H, H-C(2′)), 5.13 (t, *J* = 5.0 Hz, 1H, HO-C(5′)), 4.96 (dd, *J* = 2.5, 6.5 Hz, 1H, H-C(3′)), 4.20–4.18 (m, 1H, H-C(4′)), 3.55–3.46 (m, 2H, H_2-C(5′)), 1.73–1.70 (m, 2H, H_2-C(1a″)), 1.55 (m, 2H, H_2-C(1b″)), 1.47–1.41 (m, 2H, H_2-C(2a″)), 1.30–1.23 (m, 26H, H_2-C(2b″), H2-C(3a″), H_2-C(3b″), H_2-C(4a″–8a″), H_2-C(4b″–8b″)), 0.87–0.82 (m, 6H, H_3-C(9a″), H_3-C(9b″)).
^{13}C NMR (126 MHz, DMSO-d_6): δ 155.97 (C(6)), 152.42 (C(2)), 148.79 (C(4)), 139.63 (C(8)), 118.97 (C(5)), 116.65 (C(acetal)), 89.42 (C(1′)), 86.83 (C(4′)), 83.54 (C(2′)), 81.46 (C(3′)), 61.51 (C(5′)), 36.46 (C(1a″)), 36.37 (C(1b″)), 31.19 (C(2a″)), 31.15 (C(2b″)), 23.25 (C(3a″)), 22.96 (C(3b″)), 22.06 (C(4a″–8a″)), 22.03 (C(4b″–8b″)), 13.80 (C(9a″)), 13.78 (C(9b″)).
ESI – MS: m/z 532.28 $[M+H]^+$ (calcd for $C_{29}H_{49}N_5O_4$: 531.73).
Elemental analysis calcd (%) for $C_{29}H_{49}N_5O_4$: C 65.52, H 8.96, N 12.91; found: C 65.51, H 9.29, N 13.17.

((3a′R,4′R,6′R,6a′R)-6-(6-amino-9H-purin-9-yl)tetrahydrospiro[cyclopenta-decane-1,2′-furo[3,4-d][1,3]-dioxol]-4-yl)methanol **(4, NL_5.cycl7.0.0)** *(adapted from Hammerbacher et al., 2018)*

Dry adenosine (1, NS_5.0.0.0; 1 g, 3.74 mmol) was dissolved in 10 mL of anhydrous DMF. Cyclopentadecanone (0.848 g, 4 mmol), triethyl orthoformate (0.662 g, 4 mmol), and 7.5 mL of 4 M HCl in 1,4-dioxane were added to the solution. The reaction mixture was stirred for 24 hours at room temperature. After the reaction was complete, the mixture was poured into a separatory funnel with 80 mL CH_2Cl_2 and 40 mL of saturated aqueous sodium hydrogen carbonate. The organic phase was extracted three times with 15 mL portions of CH_2Cl_2 and subsequently washed with water (3 × 50 mL). The combined organic phases were concentrated using a rotary evaporator, and the residue was co-evaporated multiple times with CH_2Cl_2 to remove residual DMF. Remaining DMF was evacuated under high vacuum with an oil pump to afford a white solid. The crude product was purified by column chromatography on silica gel (SiO_2 60, 6.5 cm × 11.5 cm; eluent: CH_2Cl_2/MeOH, 9:1, v/v), yielding the principal product as a white, solvent-free solid (0.358 g, 43 % yield).

R_f = 0.82 (SiO_2 60, CH_2Cl_2/MeOH 9:1; v/v).
UV/Vis (MeOH): λ_{max}: 259 nm (ε=12063.7 L·mol^{-1}·cm^{-1}).
logP_{OW} (ALOGPS 3.01): 4.25 ± 0.74.
^{1}H NMR (500 MHz, DMSO-d_6): δ 8.33 (s, 1H, H-C(2)), 8.15 (s, 1H, H-C(8)), 7.28 (s, 2H, NH_2), 6.11 (d, *J* = 2.5 Hz, 1H, H-C(1′)), 5.35 (dd, *J* = 3.0, 6.5 Hz, 1H, H-C(2′)), 5.15 (t, *J* = 5.5 Hz, 1H, HO-C(5′)), 4.95 (dd, *J* = 6.0, 2.5 Hz, 1H, H-C(3′)), 4.20–4.18 (m, 1H, H-C(4′)), 3.57–3.47 (m, 2H, H_2-C(5′)), 1.79–1.76 (m, 2H, H_2-C(14a″)), 1.60–1.57 (m, 2H, H_2-C(1a″)), 1.40–1.24 (m, 24H, H_2-C(2a″)–H_2-C(13a″)).
^{13}C NMR (126 MHz, DMSO-d_6): δ 156.06 (C(6)), 152.54 (C(2)), 148.77 (C(4)), 139.68 (C(8)), 119.03 (C(5)), 116.77 (C(acetal)), 89.49 (C(1′)), 86.54 (C(4′)), 83.04 (C(2′)), 81.15 (C(3′)), 61.53 (C(5′)), 36.52 (C(14a″)), 34.16 (C(1a″)), 26.90 (C(13a″)), 26.86 (C(2a″)), 26.34 (C(12a″)), 26.32 (C(3a″)), 26.15–21.99 (8 × C, C(4a″)–C(11a″)).
ESI – MS: m/z 474.52 $[M+H]^+$ (calcd for $C_{25}H_{39}N_5O_4$: 473.30).
Elemental analysis calcd (%) for $C_{25}H_{39}N_5O_4$ · 0.8 H_2O · 0.2 Cycolpentadecanone: C 62.85, H 8.44, N 13.05; found: C 63.11, H 8.74, N 13.14.

((1R,3S,3a′R,4′R,6′R,6a′R)-4′-(6-amino-9H-purin-9-yl)tetrahydrospiro[adamantane-2,2′-furo[3,4-d][1,3]dioxol]-6′-yl)methanol **(5, NL_5.cycl8.0.0)**

Adenosine (1, NS_5.0.0.0; 1.0 g, 3.74 mmol) was dissolved in 13 mL of dry, amine-free DMF. Adamantan-2-one (0.9088 g, 4.0 mmol), triethyl orthoformate (0.662 mL, 4.0 mmol), and 6 mL of 4 M HCl in 1,4-dioxane were then added. The reaction mixture was stirred at room temperature for 24 hours. Upon completion, the mixture was partitioned between 100 mL of CH_2Cl_2 and 30 mL of saturated aqueous sodium hydrogen carbonate. The aqueous layer was extracted three times with 12.5 mL portions of CH_2Cl_2. The combined organic phases were washed with 120 mL of water and dried over magnesium sulfate. After filtration, the organic solution was concentrated under reduced pressure with a rotary evaporator, and any residual DMF

was removed by repeated co-evaporation with CH_2Cl_2. The crude product was dried overnight under high vacuum at room temperature. Crystallization from CH_2Cl_2 at 4 °C, followed by additional drying under high vacuum, yielded the purified product as a white solid (0.5746 g, 38 % yield).

R_f = 0.78 (SiO_2 60, CH_2Cl_2/MeOH 9:1; v/v).

UV/Vis (MeOH): λ_{max}: 259 nm (ε=14110.2 L·mol^{-1}·cm^{-1}).

logP_{OW} (ALOGPS 3.01): 1.43 ± 0.74.

^{1}H NMR (500 MHz, DMSO-d$_6$): δ 8.34 (s, 1H, H-C(2)), 8.16 (s, 1H, H-C(8)), 7.30 (s, 2H, NH_2), 6.13 (d, *J* = 3.0 Hz, 1H, H-C(1′)), 5.40 (dd, *J* = 3.0, 6.5 Hz, 1H, H-C(2′)), 5.18 (t, *J* = 6.0 Hz, 1H, HO-C(5′)), 4.99 (dd, *J* = 2.0, 6.0 Hz, 1H, H-C(3′)), 4.23–4.20 (m, 1H, H-C(4′)), 3.59–3.49 (m, 2H, H_2-C(5′)), 1.97–1.61 (m, 14H, H_2-C(1a″), H_2-C(1b″), H_2-C(2a″), H_2-C(2b″), H_2-C(3a″), H_2-C(3b″), H_2-C(4a″), H_2-C(4b″), H_3-C(5b″)).

^{13}C NMR (126 MHz, DMSO-d$_6$): δ 156.09 (C(6)), 152.56 (C(2)), 148.79 (C(4)), 139.78 (C(8)), 119.05 (C(5)), 115.88 (C(acetal)), 89.61 (C(1′)), 86.63 (C(4′)), 82.80 (C(2′)), 81.01 (C(3′)), 61.58 (C(5′)), 38.04 (C(1a″)), 36.38 (C(2a″)), 35.27 (C(1b″)), 34.52 (C(2b″), C(3b″)), 34.07 (C(3a″)), 33.89 (C(5b″)), 26.14 (C(4b″), C(4a″)).

ESI – MS: m/z 400.04 [M+H]$^+$ (calcd for $C_{20}H_{25}N_5O_4$: 399.44).

Elemental analysis calcd (%) for $C_{20}H_{25}N_5O_4$: C 60.63, H 6.21, N 17.48; found: C 60.14, H 6.31, N 17.53.

2.1.2. O-2′,3′-Nucleolipids of 1,N^6-Ethenodenosine

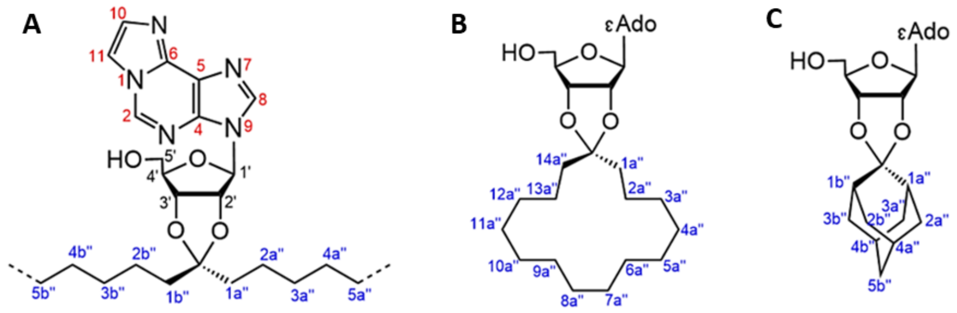

Supplementary Figure S2. Schematic illustration of the numbering of symmetrical O-2′,3′-ketalized 1,N^6-ethenoadenosine (εAdo) derivatives. (**A**) general numbering scheme for all linear alkyl chains. (**B**) Numbering of the pentadecanyliden cyclic ketal. (**C**) Numbering of the adamantanone-derived ketal.

(2R,3S,4R,5R)-2-(hydroxymethyl)-5-(3H-imidazo[2,1-i]purin-3-yl)tetrahydrofuran-3,4-diol Hydrochloride **(6, εNS_5.0.0.0, 1,N^6-Ethenoadenosine)** *(adapted from Barrio et al., 1972)*

Anhydrous adenosine (1, NS_5.0.0.0; 1.0 g, 3.74 mmol) was dissolved in 40 mL of a distilled chloroacetaldehyde solution (2–3 M). The pH of the reaction mixture was adjusted to 4–4.5 by the addition of 200 µL of a 1 M ammonium hydrogen carbonate solution. The reaction was stirred at 37 °C for 24 hours. Following completion, the solvent was removed under reduced pressure using a rotary evaporator (with the water bath maintained below 35 °C). Remaining water and chloroacetaldehyde were eliminated by repeated co-evaporation with ethanol. The crude product was recrystallized from a mixture of methanol and diethyl ether (MeOH/Et_2O). The resulting solid was triturated with diethyl ether and filtered to give the pure, solvent-free product as a white solid (1.0372 g, 95 % yield).

R_f = 0.08 (SiO_2 60, CH_2Cl_2/MeOH 9:1; v/v).

UV/Vis (MeOH): λ_{max}: 229 nm (ε=29302.0 L·mol^{-1}·cm^{-1}).

Fluorescence (MeOH): λ_{Em}: 406 nm, λ_{Ex}: 232 nm.

logP_{OW} (ALOGPS 3.01): -1.0 ± 0.74.

^{1}H NMR (500 MHz, DMSO-d$_6$): δ 9.66 (s, 1H, H-C(2)), 8.95 (s, 1H, H-C(8)), 8.49 (d, *J* = 2.0 Hz, 1H, H-C(10)), 8.11 (d, *J* = 2.0 Hz, 1H, H-C(11)), 6.13 (d, *J* = 5.0 Hz, 1H, H-C(1′)), 5.84–4.66 (m, 3H, H-O(5′), H-O(3′), H-O(2′)), 4.59 (t, *J* = 5.0 Hz, 1H, H-C(2′)), 4.23 (t, *J* = 4.5 Hz, 1H, H-C(3′)), 4.03 (q, *J* = 7.5 Hz, 1H, H-C(4′)), 3.73–3.60 (m, 2H, H_2-C(5′)).

^{13}C NMR (126 MHz, DMSO-d_6): δ 142.81 (C(6)), 142.23 (C(8)), 137.68 (C(2)), 137.24 (C(4)), 123.24 (C(5)), 111.31 (C(acetal)), 114.29 (C(11)), 88.22 (C(1′)), 85.80 (C(4′)), 74.48 (C(2′)), 70.02 (C(3′)), 60.89 (C(5′)).
ESI – MS: m/z 291.97 $[M+H]^+$, 583.07 $[2M+H]^+$ (calcd for $C_{12}H_{13}N_5O_4$: 291.26).
Elemental analysis calcd (%) for $C_{12}H_{13}N_5O_4 \cdot HCl$: C 43.67, H 4.57, N 21.34; found: C 43.98, H 4.30, N 21.97.

((3aR,4R,6R,6aR)-6-(3H-imidazo[2,1-i]purin-3-yl)-2,2-dipropyltetrahydrofuro[3,4-d][1,3]dioxol-4-yl)methanol Hydrochloride **(7a, εNL_5.4.0.0)**

Synthesis pathway 1: Dry NL_5.4.0.0 (2a, 0.1302 g, 0.358 mmol) was dissolved in 10 mL of distilled chloroacetaldehyde solution (2–3 M). The pH was adjusted to 4–4.5 by the addition of 200 μL of 1 M ammonium hydrogen carbonate solution. The reaction mixture was stirred at 37 °C for 24 hours. After completion, the solvent was removed under reduced pressure using a rotary evaporator (with the water bath kept below 35 °C), and the remaining residue was further evaporated with ethanol. The mixture was then partitioned between CH_2Cl_2 and an aqueous sodium hydrogen carbonate solution. Extraction was continued until the aqueous phase showed minimal fluorescence. The combined organic layers were washed with 120 mL of water and dried over magnesium sulfate for 1 hour. Following filtration, the crude product was concentrated using a rotary evaporator and dried overnight under high vacuum. Final purification was achieved by column chromatography on silica gel (SiO_2 60, column: 5.5 cm × 5 cm; CH_2Cl_2/MeOH 9:1, v/v). The solvent-free, isolated product was obtained as a yellowish oil (0.0609 g, 27 % yield) after drying under high vacuum.

Synthesis pathway 2: Dry ethenoadenosine (6, εNS_5.0.0.0, 0.174 g, 0.6 mmol) was dissolved in 7 mL of dry, amine-free dimethylformamide. Heptan-4-one (0.0655 mL, 0.7 mmol), triethyl orthoformate (0.12 mL), and 2 mL of 4 M HCl in 1,4-dioxane were added sequentially. The mixture was stirred at room temperature for 24 hours. After completion, the reaction mixture was partitioned between 80 mL of CH_2Cl_2 and 40 mL of saturated aqueous sodium hydrogen carbonate. The aqueous layer was extracted with CH_2Cl_2 until the fluorescence was no longer detectable. The combined organic layers were washed with 120 mL of water and dried over magnesium sulfate. After filtration, the crude product was concentrated using a rotary evaporator. Any remaining DMF was removed at 30 °C utilizing a Kugelrohr short-path distillation apparatus. The main product was purified by column chromatography on silica gel (SiO_2 60, column: 5.5 cm × 5 cm; CH_2Cl_2/MeOH 9:1, v/v) and freeze-dried from 1,4-dioxane. Following overnight drying under high vacuum, the product was obtained as a white solid (0.065 g, 28 % yield).

R_f 0.67 (SiO_2 60, CH_2Cl_2/MeOH 9:1; v/v).
UV/Vis (MeOH): λ_{max}: 229 nm (ε=33148.8 L·mol^{-1}·cm^{-1}).
Fluorescence (MeOH): λ_{Em}: 409 nm, λ_{Ex}: 235 nm.
logP_{OW} (ALOGPS 3.01): 1.71 ± 0.74.
^1H NMR (500 MHz, DMSO-d_6): δ 9.31 (s, 1H, H-C(2)), 8.53 (s, 1H, H-C(8)), 8.09 (d, *J* = 1.5 Hz, 1H, H-C(10)), 7.57 (d, *J* = 1.5 Hz, 1H, H-C(11)), 6.29 (d, *J* = 2.5 Hz, 1H, H-C(1′)), 5.41 (dd, *J* = 2.5, 6.5 Hz, 1H, H-C(2′)), 5.04–5.00 (m, 2H, H-C(3′), H-O(5′)), 4.26–4.24 (m, 1H, H-C(4′)), 3.56–3.50 (m, 2H, H_2-C(5′)), 1.75–1.72 (m, 2H, H_2-C(1a″)), 1.59–1.55 (m, 2H, H_2-C(1b″)), 1.52–1.44 (m, 2H, H_2-C(2a″)), 1.34–1.27 (m, 2H, H_2-C(2b″)), 0.96 (t, *J* = 7.5 Hz, 3H, H_3-C(3a″)), 0.87 (t, *J* = 7.5 Hz, 3H, H_3-C(3b″)).
^{13}C NMR (126 MHz, DMSO-d_6): δ 140.36 (C(6)), 139.92 (C(8)), 137.83 (C(2)), 137.04 (C(4)), 123.08 (C(5)), 116.59 (C(acetal)), 112.12 (C(11)), 89.83 (C(1′)), 87.20 (C(4′)), 84.12 (C(2′)), 81.52 (C(3′)), 61.45 (C(5′)), 16.95 (C(1a″), C(1b″)), 16.28 (C(2a″), C(2b″)), 14.08 (C(3a″)), 14.05 (C(3b″)).
ESI – MS: m/z 388.11 $[M+H]^+$, 775.22 $[2M+H]^+$ (calcd for $C_{19}H_{25}N_5O_4$: 387.43).
Elemental analysis calcd (%) for $C_{19}H_{25}N_5O_4 \cdot 0.35\ HCl \cdot 0.15\ H_2O$ · 0.1 Heptan-4-one: C 57.20, H 6.687, N 16.82; found: C 57.11, H 6.58, N 16.90.

((3aR,4R,6R,6aR)-6-(3H-imidazo[2,1-i]purin-3-yl)-2,2-dipentyltetrahydrofuro[3,4-d][1,3]dioxol-4-yl)methanol Hydrochloride **(7b, εNL_5.5.0.0)**

Synthesis pathway 1: Anhydrous compound 2b (NL_5.5.0.0, 0.1078 g, 0.257 mmol) was dissolved in 1.5 mL of an aqueous chloroacetaldehyde solution (50 % in H_2O). Sodium acetate (0.0783 g) was added to buffer the reaction mixture to a pH of approximately 4–4.5. The reaction was stirred at 37 °C for 24 hours. Subsequently, the solvent was removed under reduced pressure using a rotary evaporator (with the water bath maintained below 35 °C), and any remaining water or chloroacetaldehyde was eliminated by repeated co-evaporation with ethanol. The resulting mixture was then partitioned between CH_2Cl_2 and an aqueous sodium hydrogen carbonate solution. Extraction was continued until the aqueous phase exhibited minimal fluorescence. The combined organic layers were washed with 120 mL of water and dried over magnesium sulfate. After filtration, the crude product was concentrated using a rotary evaporator. The oily residue was dried overnight under high vacuum. Purification by column chromatography on silica gel (SiO_2 60, column: 5.5 cm × 5 cm; CH_2Cl_2/MeOH 95:5, v/v) afforded the main product as a white solid after overnight drying under high vacuum (0.0573 g, 50 % yield).

Synthesis pathway 2: Dry ethenoadenosine (6, εNS_5.0.0.0, 0.174 g, 0.6 mmol) was dissolved in 10 mL of dry, amine-free dimethylformamide. Undecan-6-one (0.099 mL, 0.7 mmol), triethyl orthoformate (0.12 mL), and 3 mL of 4 M HCl in 1,4-dioxane were added sequentially. The reaction mixture was stirred at room temperature for 24 hours. Upon completion, the mixture was partitioned between 60 mL of CH_2Cl_2 and 40 mL of saturated aqueous sodium hydrogen carbonate. The aqueous layer was extracted with CH_2Cl_2 until fluorescence was no longer detectable. The combined organic layers were washed with 120 mL of water and dried over magnesium sulfate. After filtration, the crude product was concentrated using a rotary evaporator. Any remaining DMF was removed at 30 °C using a Kugelrohr short-path distillation apparatus. The main product was purified by column chromatography on silica gel (SiO_2 60, column: 5.5 cm × 6 cm; CH_2Cl_2/MeOH 95:5, v/v). After overnight drying under high vacuum, the product was obtained as a white solid (0.0584 g, 22 % yield).

R_f 0.72 (SiO_2 60, CH_2Cl_2/MeOH 9:1; v/v).

UV/Vis (MeOH): λ_{max}: 229.0 nm (ε=38972.6 $L \cdot mol^{-1} \cdot cm^{-1}$).

Fluorescence (MeOH): λ_{Em}: 408 nm, λ_{Ex}: 235 nm.

logP_{OW} (ALOGPS 3.01): 3.28 ± 0.74.

^{1}H NMR (500 MHz, DMSO-d_6) δ 9.30 (s, 1H, H-C(2)), 8.53 (s, 1H, H-C(8)), 8.09 (d, *J* = 1.5 Hz, 1H, H-C(10)), 7.56 (d, *J* = 1.0 Hz, 1H, H-C(11)), 6.29 (d, *J* = 2.5 Hz, 1H, H-C(1′)), 5.40 (dd, *J* = 2.5, 6.5 Hz, 1H, H-C(2′)), 5.05–4.99 (m, 2H, H-C(3′), H-O(5′)), 4.26–4.23 (m, 1H, H-C(4′)), 3.55–3.50 (m, 2H, H_2-C(5′)), 1.76–1.73 (m, 2H, H_2-C(1a″)), 1.59–1.56 (m, 2H, H_2-C(1b″)), 1.49–1.43 (m, 2H, H_2-C(2a″)), 1.34–1.27 (m, 10H, H_2-C(2b″), H_2-C(3a″), H_2-C(3b″), H_2-C(4a″), H_2-C(4b″)), 1.36–1.25 (m, 6H, H_3-C(5a″), H_3-C(5b″)).

^{13}C NMR (126 MHz, DMSO-d_6) δ 140.37 (C(6)), 139.88 (C(8)), 137.87 (C(2)), 137.03 (C(4)), 132.73 (C(10)), 123.07 (C(5)), 116.72 (C(acetal)), 112.12 (C(11)), 89.75 (C(1′)), 87.21 (C(4′)), 84.13 (C(2′)), 81.51 (C(3′)), 61.45 (C(5′)), 36.28 (C(1a″), C(1b″)), 31.31 (C(2a″)), 31.28 (C(2b″)), 23.21 (C(3a″)), 22.53 (C(3b″)), 21.93 (C(4a″)), 21.88 (C(4b″)), 13.78 (C(5a″)), 13.73 (C(5b″)).

ESI – MS: m/z 444.12 $[M+H]^+$, 887.30 $[2M+H]^+$ (calcd for $C_{23}H_{33}N_5O_4$: 443.54).

Elemental analysis calcd (%) for $C_{23}H_{33}N_5O_4 \cdot 0.4\ HCl \cdot 0.05\ H_2O$ · 0.1 Undecan-6-one: C 60.97, H 7.44, N 14.53; found: C 60.8, H 7.56, N 14.71.

((3aR,4R,6R,6aR)-6-(3H-imidazo[2,1-i]purin-3-yl)-2,2-diheptyltetrahydrofuro[3,4-d][1,3]dioxol-4-yl)methanol Hydrochloride **(7c, εNL_5.7.0.0)**

*Synthesis pathway 1: The a*nhydrous compound 2c (NL_5.7.0.0, 0.1906 g, 0.4 mmol) was dissolved in 10 mL of distilled chloroacetaldehyde solution (2–3 M). To adjust the pH of the reaction mixture to 4–4.5, 200 µL

of ammonium hydrogen carbonate solution (1 M) was added. The mixture was stirred at 37 °C for 24 hours. Afterward, the solvent was removed using a rotary evaporator (ensuring the water bath did not exceed 35 °C), and remaining water/chloroacetaldehyde was eliminated by repeated co-evaporation with ethanol. The residue was partitioned between CH_2Cl_2 and aqueous $NaHCO_3$ solution, and extraction continued until the aqueous phase showed little fluorescence. The combined organic layers were washed with 120 mL H_2O and dried over $MgSO_4$. After filtration, the crude product was concentrated in a rotary evaporator. The resulting oily residue was dried overnight under high vacuum. The main product was purified by column chromatography on silica gel (SiO_2 60, column: 6.5 cm x 5 cm; CH_2Cl_2/MeOH, 93:7 v/v). After a final overnight drying under high vacuum, the purified product was obtained as a white solid (0.0839 g, 42 %).

Synthesis pathway 2: Dry ethenoadenosine (6, εNS_5.0.0.0, 0.174 g; 0.6 mmol) was dissolved in 7 mL of dry, amine-free dimethylformamide. Pentadecan-8-one (0.1585 g; 0.7 mmol), triethyl orthoformate (0.12 mL), and 4 M HCl in 1,4-dioxane (2 mL) were added to the solution. The mixture was stirred at room temperature for 24 hours. The reaction mixture was then partitioned between 60 mL of CH_2Cl_2 and 40 mL of saturated aqueous NaHCO3 solution. The aqueous phase was extracted with CH_2Cl_2 until fluorescence diminished. The combined organic layers were washed with 120 mL H_2O and dried over $MgSO_4$ for 1 hour. After filtration, the crude product was concentrated with a rotary evaporator. Residual DMF was removed at 30 °C using a Kugelrohr tube apparatus. The main product was purified by column chromatography on silica gel (SiO_2 60, column: 6.5 cm x 5 cm; CH_2Cl_2/MeOH, 93:7 v/v). The final product, isolated and solvent-free, was obtained as a white solid in high vacuum (0.0662 g, 22 %).

R_f 0.76 (SiO_2 60, CH_2Cl_2/MeOH 9:1; v/v).

UV/Vis (MeOH): λ_{max}: 229 nm (ε=33756.0 L·mol^{-1}·cm^{-1}).

Fluorescence (MeOH): λ_{Em}: 408 nm, λ_{Ex}: 233 nm.

logP_{OW} (ALOGPS 3.01): 4.34 ± 0.74.

^{1}H NMR (500 MHz, DMSO-d_6) δ 9.30 (s, 1H, H-C(2)), 8.53 (s, 1H, H-C(8)), 8.09 (d, *J* = 1.5 Hz, 1H, H-C(10)), 7.57 (d, *J* = 1.5 Hz, 1H, H-C(11)), 6.28 (d, *J* = 2.0 Hz, 1H, H-C(1′)), 5.40 (dd, *J* = 2.5, 6.5 Hz, 1H, H-C(2′)), 5.04–4.99 (m, 2H, H-C(3′), H-O(5′)), 4.25–4.22 (m, 1H, H-C(4′)), 3.55–3.50 (m, 2H, H_2-C(5′)), 1.76–1.73 (m, 2H, H_2-C(1a″)), 1.58–1.56 (m, 2H, H_2-C(1b″)), 1.45–1.45 (m, 2H, H_2-C(2a″)), 1.32–1.25 (m, 18H, H_2-C(2b″), H_2-C(3a″), H_2-C(3b″), H_2-C(4a″–6a″), H_2-C(4b″–6b″)), 0.89–0.85 (m, 6H, H_3-C(7a″), H_3-C(7b″)).

^{13}C NMR (126 MHz, DMSO-d_6) δ 140.39 (C(6)), 139.91 (C(8)), 137.90 (C(2)), 137.05 (C(4)), 132.76 (C(10)), 123.09 (C(5)), 116.76 (C(acetal)), 112.16 (C(11)), 89.77 (C(1′)), 87.22 (C(4′)), 84.14 (C(2′)), 81.51 (C(3′)), 61.47 (C(5′)), 36.34 (C(1a″)), 36.29 (C(1b″)), 31.16 (C(2a″)), 31.10 (C(2b″)), 28.64–17.41 (C(3a″), C(3b″), C(4a″–6a″), C(4b″–6b″)), 13.86 (C(7a″)), 13.82 (C(7b″)).

ESI – MS: m/z 500.26 $[M+H]^+$, 999.47 $[2M+H]^+$ (calcd for $C_{27}H_{41}N_5O_4$: 499.65).

Elemental analysis calcd (%) for $C_{27}H_{41}N_5O_4$ · 0.55 HCl · 0.1 Pentadecan-8-one: C 63.23, H 8.094, N 13.02; found: C 63.12, H 8.28, N 12.91.

((3aR,4R,6R,6aR)-6-(3H-imidazo[2,1-i]purin-3-yl)-2,2-dioctyltetrahydrofuro[3,4-d][1,3]dioxol-4-yl)methanol Hydrochloride **(7d, εNL_5.9.0.0)**

*Synthesis pathway 1: The d*ry compound 2d (NL_5.9.0.0, 0.1041 g, 0.21 mmol) was dissolved in 10 mL of distilled chloroacetaldehyde solution (2–3 M). The pH of the solution was adjusted to 4–4.5 by adding 200 µL of ammonium bicarbonate solution (1 M). The mixture was stirred at 37 °C for 24 hours. Afterwards, the solvent was removed using a rotary evaporator (keeping the water bath below 35 °C), and the residue was repeatedly co-evaporated with ethanol to eliminate any remaining aqueous chloroacetaldehyde. The crude product was then dried overnight under high vacuum. Purification was achieved by column chromatography on silica gel (SiO_2 60, column: 8 cm × 5 cm; CH_2Cl_2/MeOH, 9:1 v/v). After drying under high vacuum, the isolated, solvent-free product was obtained as a white solid (0.065 g, 59 %).

Synthesis pathway 2: Anhydrous ethenoadenosine (6, εNS_5.0.0.0, 0.2002 g; 0.69 mmol) was dissolved in 8 mL of dry, amine-free dimethylformamide. Heptadecan-9-one (0.1780 g; 0.7 mmol), triethyl orthoformate (0.16 mL), 4 M HCl in 1,4-dioxane (4 mL), and CH_2Cl_2 (1.5 mL) were then added. The mixture was stirred at room temperature for 24 hours. The reaction mixture was subsequently partitioned between 60 mL CH_2Cl_2 and 40 mL saturated aqueous NaHCO3 solution. The aqueous phase was extracted with CH_2Cl_2 until fluorescence was no longer observed. The combined organic phases were dried over $MgSO_4$ for 1 hour. After filtration, the crude product was concentrated in a rotary evaporator and dried overnight under high vacuum. Purification was performed by column chromatography on silica gel (SiO_2 60, column: 6.5 cm × 5 cm; CH_2Cl_2/MeOH, 9:1 v/v). The final, solvent-free product was obtained as a white solid under high vacuum (0.0874 g, 24 %).

R_f 0.79 (SiO_2 60, CH_2Cl_2/MeOH 9:1; v/v).

UV/Vis (MeOH): λ_{max}: 229 nm (ε=28007.2 L·mol^{-1}·cm^{-1}).

Fluorescence (MeOH): λ_{Em}: 409 nm, λ_{Ex}: 233 nm.

logP_{OW} (ALOGPS 3.01): 4.76 ± 0.74.

^{1}H NMR (500 MHz, DMSO-d$_6$) δ 9.30 (s, 1H, H-C(2)), 8.53 (s, 1H, H-C(8)), 8.09 (d, *J* = 1.5 Hz, 1H, H-C(10)), 7.57 (d, *J* = 1.5 Hz, 1H, H-C(11)), 6.28 (d, *J* = 2.5 Hz, 1H, H-C(1′)), 5.39 (dd, *J* = 2.5, 6.5 Hz, 1H, H-C(2′)), 5.03 (t, *J* = 5.0 Hz, 1H, HO-C(5′)), 5.01–4.99 (m, 2H, H-C(3′), H-O(5′)), 4.25–4.22 (m, 1H, H-C(4′)), 3.56–3.50 (m, 2H, H_2-C(5′)), 1.76–1.72 (m, 2H, H_2-C(1a″)), 1.59–1.56 (m, 2H, H_2-C(1b″)), 1.46–1.42 (m, 2H, H_2-C(2a″)), 1.31–1.23 (m, 22H, H_2-C(2b″)–H_2-C(7b″), H_2-C(3a″)–H_2-C(7a″)), 0.87–0.84 (m, 6H, H_3-C(8a″), H_3-C(8b″)).

^{13}C NMR (126 MHz, DMSO-d$_6$) δ 140.35 (C(6)), 139.89 (C(8)), 137.87 (C(2)), 137.00 (C(4)), 132.68 (C(10)), 123.06 (C(5)), 116.71 (C(acetal)), 112.12 (C(11)), 89.74 (C(1′)), 87.19 (C(4′)), 84.11 (C(2′)), 81.46 (C(3′)), 61.43 (C(5′)), 36.31 (C(1a″)), 36.21 (C(1b″)), 31.16 (C(2a″)), 31.13 (C(2b″)), 30.52 (C(3a″)), 29.06 (C(3b″)), 28.80–28.46 (4 × C, C(4a″)–C(7a″)), 23.52–21.93 (4 × C, C(4b″)–C(7b″)), 13.80 (C(8a″)), 13.77 (C(8b″)).

ESI – MS: m/z 528.35 [M+H]$^+$, 1055.7 [2M+H]$^+$ (calcd for $C_{29}H_{45}N_5O_4$: 527.70).

Elemental analysis calcd (%) for $C_{29}H_{45}N_5O_4$ · 0.35 HCl · 0.5 H_2O · 0.05 Pentadecan-8-one: C 63.79, H 8.71, N 12.37; found: C 63.77, H 8.61, N 12.46.

((3aR,4R,6R,6aR)-6-(3H-Imidazo[2,1-i]purin-3-yl)-2,2-dinonyltetrahydrofuro-[3,4-d][1,3]dioxol-4-yl)methanol Hydrochloride **(7e, εNL_5.3.0.0)** *(adapted from Knies, 2017)*

Synthesis pathway 1: The anhydrous compound 2e (NL_5.3.0.0, 0.1357 g, 0.255 mmol) was dissolved in 10 mL of distilled chloroacetaldehyde solution (2–3 M). The pH was adjusted to 4–4.5 by adding 200 µL of ammonium hydrogen carbonate solution (1 M). The mixture was stirred at 37 °C for 24 hours. Subsequently, the solvent was removed using a rotary evaporator (with the water bath kept below 35 °C), and the remaining water/chloroacetaldehyde was eliminated by repeated co-evaporation with ethanol. The residue was then partitioned between CH_2Cl_2 and aqueous $NaHCO_3$ solution, and extraction continued until the aqueous phase showed almost no fluorescence. The combined organic layers were washed with 120 mL H_2O and dried over $MgSO_4$ for 1 hour. After filtration, the crude product was concentrated in a rotary evaporator and dried overnight under high vacuum. Purification was achieved by column chromatography on silica gel (SiO_2 60, column: 5.5 cm × 5 cm; CH_2Cl_2/MeOH, 9:1 v/v). The isolated, solvent-free product was obtained as a white solid (0.0489 g, 35 %) after high vacuum drying.

Synthesis pathway 2: Dry adenosine (6, εNS_5.0.0.0, 0.174 g; 0.6 mmol) was dissolved in 10 mL of dry, amine-free dimethylformamide. Nonadecan-10-one (0.1977 g; 0.7 mmol), triethyl orthoformate (0.12 mL), and 4 M HCl in 1,4-dioxane (3 mL) were added. The mixture was stirred at room temperature for 24 hours. The reaction mixture was then partitioned between 60 mL CH_2Cl_2 and 40 mL saturated aqueous $NaHCO_3$ solution. The aqueous phase was extracted with CH2Cl2 until fluorescence had disappeared. The combined

organic layers were dried over $MgSO_4$ for 30 minutes and concentrated using a rotary evaporator. Residual DMF was removed at 30 °C with a Kugelrohr tube apparatus. Purification was performed by column chromatography on silica gel (SiO_2 60, column: 5.5 cm × 6 cm; CH_2Cl_2/MeOH, 93:7 v/v). The final, solvent-free product was isolated as a white solid under high vacuum (0.1087 g, 33 %).

R_f 0.80 (SiO_2 60, CH_2Cl_2/MeOH 9:1; v/v).

UV/Vis (MeOH): λ_{max}: 229 nm (ε=35056.9 $L \cdot mol^{-1} \cdot cm^{-1}$).

Fluorescence (MeOH): λ_{Em}: 409 nm, λ_{Ex}: 233 nm.

logP_{OW} (ALOGPS 3.01): 5.17 ± 0.74.

^{1}H NMR (500 MHz, DMSO-d$_6$) δ 9.30 (s, 1H, H-C(2)), 8.53 (s, 1H, H-C(8)), 8.09 (d, *J* = 1.5 Hz, 1H, H-C(10)), 7.56 (d, *J* = 1.5 Hz, 1H, H-C(11)), 6.28 (d, *J* = 2.5 Hz, 1H, H-C(1′)), 5.39 (dd, *J* = 2.5, 6.5 Hz, 1H, H-C(2′)), 5.04–4.99 (m, 2H, H-C(3′), H-O(5′)), 4.25–4.22 (m, 1H, H-C(4′)), 3.54–3.51 (m, 2H, H_2-C(5′)), 1.75–1.72 (m, 2H, H_2-C(1a″)), 1.58–1.56 (m, 2H, H_2-C(1b″)), 1.46–1.44 (m, 2H, H_2-C(2a″)), 1.31–1.22 (m, 26H, H_2-C(2b″), H_2-C(3a″), H_2-C(3b″), H_2-C(4a″–8a″), H_2-C(4b″–8b″)), 0.86–0.82 (m, 6H, H_3-C(9a″), H_3-C(9b″)).

^{13}C NMR (126 MHz, DMSO-d$_6$) δ 140.37 (C(6)), 139.87 (C(8)), 137.85 (C(2)), 136.99 (C(4)), 132.73 (C(10)), 123.09 (C(5)), 116.71 (C(acetal)), 112.10 (C(11)), 89.74 (C(1′)), 87.19 (C(4′)), 84.11 (C(2′)), 81.46 (C(3′)), 61.44 (C(5′)), 36.32 (C(1a″)), 36.19 (C(1b″)), 31.19 (C(2a″)), 31.14 (C(2b″)), 29.04–21.94 (C(3a″), C(3b″), C(4a″–8a″), C(4b″–8b″)), 13.79 (C(9a″)), 13.76 (C(9b″)).

ESI – MS: 556.35 $[Mw+H]^+$, 1111.65 $[2Mw+H]^+$ (calcd for $C_{31}H_{49}N_5O_4$: 555.75).

Elemental analysis calcd (%) for $C_{31}H_{49}N_5O_4 \cdot 0.25\ HCl \cdot 0.25\ H_2O \cdot 0.05$ Nonadecan-10-one: C 65.77, H 9.01, N 12.05; found: C 65.77, H 8.92, N 12.00.

((3a′R,4′R,6′R,6a′R)-4′-(3H-imidazo[2,1-i]purin-3-yl)tetrahydrospiro[cyclopentadecane-1,2′-furo[3,4-d][1,3]dioxol]-6′-yl)methanol Hydrochloride **(9, εNL_5.cycl7.0.0)**

*Synthesis pathway 1: The d*ry compound 4 (NL_5.cycl7.0.0, 0.1271 g, 0.26 mmol) was dissolved in 10 mL of distilled chloroacetaldehyde solution (2–3 M). The pH was adjusted to 4–4.5 by adding 200 µL of ammonium bicarbonate solution (1 M). The mixture was stirred at 37 °C for 24 hours. Afterwards, the solvent was removed using a rotary evaporator (making sure the water bath did not exceed 35 °C), and the residue was repeatedly co-evaporated with ethanol to remove any remaining aqueous chloroacetaldehyde. The crude product was then dried overnight under high vacuum. Purification was achieved by column chromatography on silica gel (SiO_2 60, column: 7 cm × 5 cm; CH2Cl2/MeOH, 9:1 v/v). The final, solvent-free product was obtained as a white solid (0.072 g, 56 %) after high vacuum drying.

Synthesis pathway 2: Anhydrous ethenoadenosine (6, εNS_5.0.0.0, 0.2000 g; 0.69 mmol) was dissolved in 8 mL of dry, amine-free dimethylformamide. Cyclopentadecanone (0.1564 g; 0.7 mmol), triethyl orthoformate (0.16 mL), 4 M HCl in 1,4-dioxane (3 mL), and CH_2Cl_2 (0.5 mL) were then added. The mixture was stirred at room temperature for 24 hours. The reaction mixture was then partitioned between 60 mL CH2Cl2 and 40 mL saturated aqueous $NaHCO_3$ solution. The aqueous phase was extracted with CH_2Cl_2 until fluorescence was no longer observed. The combined organic layers were dried over $MgSO_4$ for 1 hour. After filtration, the crude product was concentrated with a rotary evaporator and dried overnight under high vacuum. Purification was performed by column chromatography on silica gel (SiO_2 60, column: 6.5 cm × 5 cm; CH_2Cl_2/MeOH, 9:1 v/v). The final, solvent-free product was isolated as a white solid under high vacuum (0.1172 g, 34 %).

R_f 0.76 (SiO_2 60, CH_2Cl_2/MeOH 9:1; v/v).

UV/Vis (MeOH): λ_{max}: 229 nm (ε=30631.1 $L \cdot mol^{-1} \cdot cm^{-1}$).

Fluorescence (MeOH): λ_{Em}: 409 nm, λ_{Ex}: 234 nm.

logP_{OW} (ALOGPS 3.01): 4.21 ± 0.74.

^{1}H NMR (500 MHz, DMSO-d$_6$) δ 9.31 (s, 1H, H-C(2)), 8.52 (s, 1H, H-C(8)), 8.09 (d, *J* = 1.5 Hz, 1H, H-C(10)), 7.57 (d, *J* = 1.5 Hz, 1H, H-C(11)), 6.27 (d, *J* = 2.5 Hz, 1H, H-C(1′)), 5.38 (dd, *J* = 3.0, 6.5 Hz, 1H, H-C(2′)), 5.04 (t, *J* = 5.5 Hz, 1H, HO-C(5′)), 4.98 (dd, *J* = 3.0, 6.5 Hz, 1H, H-C(3′)), 4.25–4.22 (m, 1H, H-C(4′)), 3.58–3.50 (m, 2H, H$_2$-C(5′)), 1.81–1.78 (m, 2H, H$_2$-C(1a″)), 1.61–1.58 (m, 2H, H$_2$-C(14a″)), 1.41–1.30 (m, 24H, H$_2$-C(2a″)–H$_2$-C(13a″)).
^{13}C NMR (126 MHz, DMSO-d$_6$) δ 140.35 (C(6)), 139.93 (C(8)), 137.85 (C(2)), 137.03 (C(4)), 132.70 (C(10)), 123.10 (C(5)), 116.84 (C(acetal)), 112.13 (C(11)), 89.75 (C(1′)), 86.93 (C(4′)), 83.65 (C(2′)), 81.13 (C(3′)), 61.42 (C(5′)), 36.46 (C(1a″)), 34.18 (C(14a″)), 26.90 (C(2a″)), 26.86 (C(13a″)), 26.32 (C(3a″)), 26.31 (C(12a″)), 26.14–21.98 (8 × C, C(4a″)–C(11a″)).
ESI – MS: m/z 498.31 [M+H]$^+$, 995.6 [2M+H]$^+$ (calcd for $C_{27}H_{39}N_5O_4$: 497.63).
Elemental analysis calcd (%) for $C_{27}H_{39}N_5O_4 \cdot 0.2$ HCl: C 64.24, H 7.93, N 13.77; found: C 64.23, H 7.83, N 13.87.

((1R,3S,3a′R,4′R,6′R,6a′R)-4′-(3H-imidazo[2,1-i]purin-3-yl)tetrahydrospiro[adamantane-2,2′-furo[3,4-d][1,3]dioxol]-6′-yl)methanol Hydrochloride **(10, εNL_5.cycl8.0.0)**

*Synthesis pathway 1: The d*ry compound 5 (NL_5.cycl8.0.0, 0.0484 g, 0.121 mmol) was dissolved in 10 mL of distilled chloroacetaldehyde solution (2–3 M). The pH was adjusted to 4–4.5 by adding 200 µL of ammonium hydrogen carbonate solution (1 M). The reaction mixture was stirred at 37 °C for 24 hours. Subsequently, the solvent was removed using a rotary evaporator (ensuring the water bath remained below 35 °C), and any remaining water/chloroacetaldehyde was eliminated through repeated co-evaporation with ethanol. The mixture was then partitioned between CH_2Cl_2 and aqueous $NaHCO_3$ solution, with extraction continuing until the aqueous phase showed almost no fluorescence. The combined organic phases were washed with 120 mL H_2O and dried over $MgSO_4$ for 1 hour. After filtration and concentration using a rotary evaporator, the crude product was dried overnight under high vacuum. Purification was performed by column chromatography on silica gel (SiO_2 60, column: 5.5 cm × 5 cm; CH_2Cl_2/MeOH, 9:1 v/v). The isolated, solvent-free product was obtained as a white solid (0.0164 g, 32 %) after drying under high vacuum.

Synthesis pathway 2: Dry ethenoadenosine (6, εNS_5.0.0.0, 0.174 g; 0.6 mmol) was dissolved in 5 mL of dry, amine-free dimethylformamide. Adamantan-2-one (0.1050 g; 0.7 mmol), triethyl orthoformate (0.12 mL), and 4 M HCl in 1,4-dioxane (1.5 mL) were added. The mixture was stirred at room temperature for 24 hours. The reaction mixture was then partitioned between 60 mL CH_2Cl_2 and 40 mL saturated aqueous $NaHCO_3$ solution. The aqueous phase was extracted with CH_2Cl_2 until fluorescence was no longer observed. The combined organic layers were washed with 120 mL H_2O and dried over $MgSO_4$ for 1 hour. After filtration and concentration with a rotary evaporator, the crude product was dried overnight under high vacuum. Residual DMF was removed at 30 °C using a Kugelrohr tube apparatus. Purification was performed by column chromatography on silica gel (SiO_2 60, column: 5.5 cm × 5 cm; CH_2Cl_2/MeOH, 9:1 v/v). The final, solvent-free product was obtained as a white solid under high vacuum (0.0698 g, 27 %).

R_f 0.71 (SiO_2 60, CH_2Cl_2/MeOH 9:1; v/v).
UV/Vis (MeOH): λ_{max}: 229 nm (ε=26543.7 L·mol^{-1}·cm^{-1}).
Fluorescence (MeOH): λ_{Em}: 409 nm, λ_{Ex}: 233 nm.
logP_{OW} (ALOGPS 3.01): 1.85 ± 0.74.
^{1}H NMR (500 MHz, DMSO-d$_6$) δ 9.31 (s, 1H, H-C(2)), 8.54 (s, 1H, H-C(8)), 8.09 (d, *J* = 1.5 Hz, 1H, H-C(10)), 7.57 (d, *J* = 1.5 Hz, 1H, H-C(11)), 6.29 (d, *J* = 2.5 Hz, 1H, H-C(1′)), 5.44 (dd, *J* = 3.0, 6.0 Hz, 1H, H-C(2′)), 5.06–5.03 (m, 2H, H-C(3′), H-O(5′)), 4.26–4.24 (m, 1H, H-C(4′)), 3.57–3.51 (m, 2H, H$_2$-C(5′)), 2.00–1.63 (m, 14H, H$_2$-C(1a″), H$_2$-C(1b″), H$_2$-C(2a″), H$_2$-C(2b″), H$_2$-C(3a″), H$_2$-C(3b″), H$_2$-C(4a″), H$_2$-C(4b″), H$_3$-C(5b″)).
^{13}C NMR (126 MHz, DMSO-d$_6$) δ 140.36 (C(6)), 140.05 (C(8)), 137.87 (C(2)), 137.07 (C(4)), 132.73 (C(10)), 123.11 (C(5)), 115.97 (C(acetal)), 112.15 (C(11)), 89.80 (C(1′)), 87.06 (C(4′)), 83.40 (C(2′)), 80.99 (C(3′)), 61.45 (C(5′)), 38.02 (C(1a″)), 36.37 (C(2a″)), 35.32 (C(1b″)), 34.53 (C(2b″), C(3a″)), 34.05 (C(3b″)), 33.93 (C(5b″)), 26.12 (C(4a″), C(4b″)).
ESI – MS: m/z 424.11 [M+H]$^+$, 847.26 [2M+H]$^+$ (calcd for $C_{22}H_{25}N_5O_4$: 423.4).
Elemental analysis calcd (%) for $C_{22}H_{25}N_5O_4 \cdot 0.35$ HCl · 0.3 H_2O · 0.05 Adamantan-2-one: C 60.27, H 6.18, N 15.41; found: C 60.17, H 5.98, N 15.59.

2.2. Asymmetrically O-2′,3′-Ketalized Nucleolipids

2.2.1. O-2′,3′-Nucleolipids of Adenosine

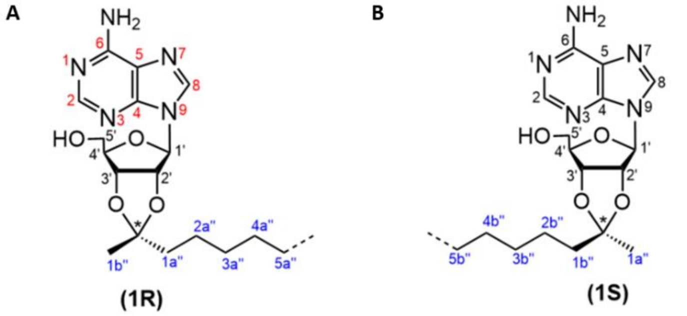

Supplementary Figure S3. Schematic illustration of the numbering of asymmetrical O-2′,3′-ketalized adenosine (Ado) derivatives. (**A**) general numbering scheme for 1R diastereoisomer. (**B**) general numbering scheme for 1S diastereoisomer.

((2R/2S,3aR,4R,6R,6aR)-6-(6-amino-9H-purin-9-yl)-2-methyl-2-pentyltetrahydrofuro[3,4-d][1,3]dioxol-4-yl)methanol ***(2f,*** **NL_5.4₂.0.0, diastereoisomeric mixture, (1R) + (1S))**

Anhydrous adenosine (1, NS_5.0.0.0; 0.8 g; 2.99 mmol) was dissolved in 6.5 mL of dry, amine-free dimethylformamide. Heptan-2-one (0.430 mL; 3.0 mmol), triethyl orthoformate (0.44 mL), and 4 M HCl in 1,4-dioxane (3 mL) were then added. The reaction mixture was stirred at room temperature for 24 hours. It was subsequently partitioned between 60 mL CH_2Cl_2 and saturated aqueous $NaHCO_3$ solution. The aqueous layer was extracted three times with 12.5 mL CH_2Cl_2 each. The combined organic layers were dried over $MgSO_4$ for 30 minutes, filtered, and concentrated using a rotary evaporator. Remaining DMF was removed by repeated co-evaporation, followed by overnight drying under high vacuum. The crude product was purified by column chromatography on silica gel (SiO_2 60, column: 6.5 × 8 cm; CH_2Cl_2/MeOH, 95:5 v/v), yielding the main product as a white solid (0.450 g, 41 %).

R_f 0.76 (SiO_2 60, CH_2Cl_2/MeOH 9:1; v/v).

UV/VIS (MeOH): λ_{max}: 260 nm (ε=15474.1 L·mol^{-1}·cm^{-1}).

logP_{OW} (ALOGPS 3.01): 2.44 ± 0.74.

^{1}H NMR (500 MHz, DMSO-d$_6$, 30 °C) δ 8.34 (s, 1H, H-C(2)), 8.16 (s, 1H, H-C(8)), 7.29 (s, 2H, NH_2), 6.14 (d, *J* = 3.0 Hz, 1H, H-C(1′), (1S)), 6.12 (d, *J* = 2.5 Hz, 1H, H-C(1′), (1R)), 5.38 (dd, *J* = 3.0, 6.0 Hz, 1H, H-C(2′), (1R)), 5.34 (dd, *J* = 3.0, 6.0 Hz, 1H, H-C(2′), (1S)), 5.17 (t, *J* = 6.0 Hz, 1H, HO-C(5′), (1S)), 5.14 (t, *J* = 5.5 Hz, 1H, HO-C(5′), (1R)), 4.98 (dd, *J* = 2.5, 6.0 Hz, 1H, H-C(3′), (1R)), 4.95 (dd, *J* = 2.5, 6.5 Hz, 1H, H-C(3′), (1S)), 4.23–4.19 (m, 1H, H-C(4′), (1R) + (1S)), 3.58–3.48 (m, 2H, H_2-C(5′), (1R) + (1S)), 1.75 (dt, *J* = 7.5, 10.5 Hz, 2H, H_2-C(1a″), (1R)), 1.57 (dt, *J* = 6.5, 10.5 Hz, 2H, H_2-C(1b″), (1S)), 1.50 (s, 3H, H_3-C(acetal), (1S)), 1.48–1.43 (m, 2H, H_2-C(2a″), (1R), H_2-C(2b″), (1S)), 1.32–1.23 (m, 4H, H_2-C(3a″), H_2-C(4a″), (1R), H_2-C(3b″), H_2-C(4b″), (1S)), 1.28 (s, 3H, H_3-C(acetal), (1R)), 0.89 (t, *J* = 7.0 Hz, 3H, H_3-C(5a″), (1R)), 0.85 (t, *J* = 7.0 Hz, 3H, H_3-C(5b″), (1S)).

^{13}C NMR (126 MHz, DMSO-d$_6$, 30 °C) δ 156.05 (C(6)), 152.55 (C(2), (1R) + (1S)), 148.81 (C(4), (1R)), 148.78 (C(4), (1S)), 139.64 (C(8), (1S)), 139.61 (C(8), (1R)), 119.03 (C(5), (1R) + (1S)), 115.01 (C(acetal), (1S)), 114.63 (C(acetal), (1R)), 89.61 (C(1′), (1S)), 89.38 (C(1′), (1R)), 86.58 (C(4′), (1S)), 86.52 (C(4′), (1R)), 83.53 (C(2′), (1S)), 83.13 (C(2′), (1R)), 81.61 (C(3′), (1S)), 81.12 (C(3′), (1R)), 61.53 (C(5′), (1R) + (1S)), 39.97 (C(1a″), (1R)), 38.21 (C(1b″), (1S)), 31.27 (C(3a″), (1R), C(3b″), (1S)), 24.97 (C(acetal), (1S)), 23.44 (C(2b″), (1S)), 23.29 (C(acetal), (1R)), 22.84 (C(2a″), (1R)), 21.93 (C(4a″), (1R)), 21.91 (C(4b″), (1S)), 13.78 (C(5a″), (1R)), 13.74 (C(5b″), (1S)).

ESI – MS: m/z 364.14 [M+H]$^+$ (calcd for $C_{17}H_{25}N_5O_4$: 363.41).

Elemental analysis calcd (%) for $C_{17}H_{25}N_5O_4$: C 56.15, H 6.858, N 19.14; found: C 56.18, H 6.93, N 19.27.

((2R/2S,3aR,4R,6R,6aR)-6-(6-amino-9H-purin-9-yl)-2-methyl-2-nonyltetrahydrofuro[3,4-d][1,3]dioxol-4-yl)methanol **(2g, NL_5.5$_2$.0.0, diastereoisomeric mixture, (1R) + (1S))**

A solution of dry adenosine (1, NS_5.0.0.0; 1.0 g, 3.74 mmol) in 5 mL dry, amine-free dimethylformamide was prepared. Subsequently, undecan-2-one (0.825 mL, 4 mmol), triethyl orthoformate (0.662 mL, 4 mmol), and 4 M HCl in 1,4-dioxane (7.5 mL) were added. The mixture was stirred at room temperature for 24 hours. After completion, the reaction mixture was partitioned between 80 mL CH_2Cl_2 and 40 mL saturated aqueous $NaHCO_3$ solution. The aqueous phase was extracted three times with 12.5 mL CH_2Cl_2. The combined organic layers were dried over $MgSO_4$ for 30 minutes, filtered, and concentrated by rotary evaporation. Remaining DMF was removed via repeated co-evaporation with CH_2Cl_2, followed by drying under high vacuum. The crude product was purified by column chromatography on silica gel (SiO_2 60, column: 6.5 cm × 11 cm; CH_2Cl_2/MeOH, 9:1 v/v), affording the main product as a white solid (0.8472 g, 54 %).

R_f 0.82 (SiO_2 60, CH_2Cl_2/MeOH 9:1; v/v).

UV/Vis (MeOH): λ_{max}: 260 nm (ε=15156.1 L·mol^{-1}·cm^{-1}).

logP_{OW} (ALOGPS 3.01): 3.69 ± 0.74.

^{1}H NMR (500 MHz, DMSO-d_6) δ 8.34 (s, 1H, H-C(2), (1R) + (1S)), 8.15 (s, 1H, H-C(8), (1R) + (1S)), 7.29 (s, 2H, NH_2), 6.14 (d, *J* = 3.0 Hz, 1H, H-C(1′), (1S)), 6.12 (d, *J* = 3.0 Hz, 1H, H-C(1′), (1R)), 5.37 (dd, *J* = 3.0, 6.0 Hz, 1H, H-C(2′), (1R)), 5.33 (dd, *J* = 3.0, 6.0 Hz, 1H, H-C(2′), (1S)), 5.17 (t, *J* = 5.5 Hz, 1H, HO-C(5′), (1S)), 5.14 (t, *J* = 5.5 Hz, 1H, HO-C(5′), (1R)), 4.98 (dd, *J* = 2.5, 6.5 Hz, 1H, H-C(3′), (1R)), 4.95 (dd, *J* = 2.5, 6.0 Hz, 1H, H-C(3′), (1S)), 4.22–4.18 (m, 1H, H-C(4′), (1R) + (1S)), 3.57–3.50 (m, 2H, H_2-C(5′), (1R) + (1S)), 1.77–1.74 (m, 2H, H_2-C(1a″), (1R)), 1.59–1.56 (m, 2H, H_2-C(1b″), (1S)), 1.49 (s, 3H, H_3-C(acetal), (1S)), 1.47–1.23 (m, 17H, H_3-C(acetal), (1R), H_2-C(2a″)–H_2-C(8a″), (1R), H_2-C(2b″)–H_2-C(8b″), (1S)), 0.87–0.82 (m, 6H, H_3-C(9a″), H_3-C(9b″), (1R) + (1S)).

^{13}C NMR (126 MHz, DMSO-d_6) δ 156.07 (C(6), (1S)), 156.05 (C(6), (1R)), 152.53 (C(2), (1R) + (1S)), 148.81 (C(4), (1R)), 148.76 (C(4), (1S)), 139.63 (C(8), (1S)), 139.56 (C(8), (1R)), 119.04 (C(5), (1S)), 119.01 (C(5), (1R)), 115.00 (C(acetal), (1S)), 114.63 (C(acetal), (1R)), 89.60 (C(1′), (1S)), 89.36 (C(1′), (1R)), 86.48 (C(4′), (1S)), 86.48 (C(4′), (1R)), 83.53 (C(2′), (1S)), 83.12 (C(2′), (1R)), 81.59 (C(3′), (1S)), 81.10 (C(3′), (1R)), 61.54 (C(5′), (1R) + (1S)), 39.01 (C(1a″), (1R)), 38.18 (C(1b″), (1S)), 31.16 (C(3a″), (1R)), 31.12 (C(3b″), (1S)), 29.04–28.53 (8 × C, C(4a″)–C(7a″), (1R), C(4b″)–C(7b″), (1S)), 24.96 (C(acetal), (1S)), 23.76 (C(2b″), (1S)), 23.30 (C(acetal), (1R)), 23.18 (C(2a″), (1R)), 21.99 (C(8b″), (1S)), 21.93 (C(8a″), (1R)), 13.79 (C(9a″), (1R)), 13.76 (C(9b″), (1S)).

ESI – MS: m/z 420.26 $[M+H]^+$ (calcd for $C_{21}H_{33}N_5O_4$: 419.52).

Elemental analysis calcd (%) for $C_{21}H_{33}N_5O_4 \cdot 0.55\ H_2O$: C 59.24, H 8.075, N 15.78; found: C 58.74, H 8.00, N 16.31.

((2R/2S,3aR,4R,6R,6aR)-6-(6-amino-9H-purin-9-yl)-2-methyl-2-tridecyltetrahydrofuro[3,4-d][1,3]dioxol-4-yl)methanol **(2h, NL_5.7$_2$.0.0, diastereoisomeric mixture, (1R) + (1S))**

A solution of anhydrous adenosine (1, NS_5.0.0.0; 1.0 g, 3.74 mmol) in 10 mL dry, amine-free dimethylformamide was prepared. Pentadecan-2-one (0.906 g, 4 mmol), triethyl orthoformate (0.662 mL, 4 mmol), and 4 M HCl in 1,4-dioxane (4 mL) were then added. The reaction mixture was stirred at room temperature for 24 hours. Afterwards, it was partitioned between 80 mL CH_2Cl_2 and 40 mL saturated aqueous $NaHCO_3$ solution. The aqueous phase was extracted three times with 15 mL CH_2Cl_2 each. The combined organic layers were washed three times with 50 mL H_2O, dried over $MgSO_4$ for 30 minutes, filtered, and concentrated using a rotary evaporator. Residual DMF was removed by repeated co-evaporation with CH_2Cl_2, followed by overnight drying under high vacuum. The crude product was purified by column chromatography on silica gel (SiO_2 60, column: 5 cm × 7 cm; CH_2Cl_2/MeOH, 9:1 v/v), yielding the title compound as a white solid after evaporation of the main fractions and drying in high vacuum (1.1918 g, 67 %).

R_f 0.82 (SiO_2 60, CH_2Cl_2/MeOH 9:1; v/v).

UV/Vis (MeOH): λ_{max}: 260 nm (ε=14260.4 L·mol^{-1}·cm^{-1}).
logP_{OW} (ALOGPS 3.01): 4.41 ± 0.74.
^{1}H NMR (500 MHz, DMSO-d$_6$) δ 8.33 (s, 1H, H-C(2), (1R) + (1S)), 8.15 (s, 1H, H-C(8), (1R) + (1S)), 7.28 (s, 2H, NH_2), 6.14 (d, J = 3.0 Hz, 1H, H-C(1′), (1S)), 6.12 (d, J = 3.0 Hz, 1H, H-C(1′), (1R)), 5.37 (dd, J = 3.0, 6.5 Hz, 1H, H-C(2′), (1R)), 5.29 (dd, J = 3.5, 6.5 Hz, 1H, H-C(2′), (1S)), 5.16–5.13 (m, 2H, HO-C(5′), (1R) + (1S)), 4.98 (dd, J = 2.5, 6.5 Hz, 1H, H-C(3′), (1R)), 4.95 (dd, J = 2.5, 6.0 Hz, 1H, H-C(3′), (1S)), 4.22–4.18 (m, 1H, H-C(4′), (1R) + (1S)), 3.57–3.48 (m, 2H, H_2-C(5′), (1R) + (1S)), 1.77–1.73 (m, 2H, H_2-C(a1′′), (1R)), 1.57–1.40 (m, 5H, H_2-C(a1′′), H_3-C(b1′′), (1S)), 1.30–1.22 (m, 25H, H-C(b1′′), H_2-C(a2′′)–H_2-C(a12′′), (1R), H_2-C(a2′′)–H_2-C(a12′′), (1S)), 0.86–0.83 (m, 6H, H-C(a13′′), H-C(a13′′), (1R) + (1S)).
^{13}C NMR (126 MHz, DMSO-d$_6$) δ 156.05 (C(6), (1R) + (1S)), 152.54 (C(2), (1R) + (1S)), 148.82 (C(4), (1R) + (1S)), 139.62 (C(8), (1R) + (1S)), 119.01 (C(5), (1R) + (1S)), 115.02 (C(acetal), (1S)), 114.64 (C(acetal), (1R)), 89.61 (C(1′), (1S)), 89.37 (C(1′), (1R)), 86.63 (C(4′), (1S)), 86.48 (C(4′), (1R)), 83.57 (C(2′), (1S)), 83.14 (C(2′), (1R)), 81.61 (C(3′), (1S)), 81.11 (C(3′), (1R)), 61.52 (C(5′), (1R) + (1S)), 31.20 (C(a11′′), (1R) + (1S)), 29.06–28.59 (10 × C, C(a1′′)–C(a10′′), (1R) + (1S)), 23.31 (C(b1′′), (1R)), 23.18 (C(b1′′), (1S)), 21.98 (C(a12′′), (1R) + (1S)), 13.82 (C(a13′′), (1R) + (1S)).
ESI – MS: m/z 476.32 [M+H]$^+$ (calcd for $C_{25}H_{41}N_5O_4$: 475.62).
Elemental analysis calcd (%) for $C_{25}H_{41}N_5O_4 \cdot 0.35\ H_2O$: C 62.62, H 8.71, N 14.17; found: C 62.31, H 8.72, N 14.53.

((2R/2S,3aR,4R,6R,6aR)-6-(6-amino-9H-purin-9-yl)-2-ethyl-2-tridecyltetrahydrofuro[3,4-d][1,3]dioxol-4-yl)methanol **(2i, NL_5.8$_3$.0.0, diastereoisomeric mixture, (1R) + (1S))**

Anhydrous adenosine (1, NS_5.0.0.0; 0.8 g, 2.99 mmol) was first dissolved in dry, amine-free dimethylformamide. To this solution, hexadecan-3-one (1.08 g, 4.5 mmol), triethyl orthoformate (0.745 mL), and 4 M HCl in 1,4-dioxane (5 mL) were added. The resulting mixture was stirred at room temperature for 24 hours. Upon completion of the reaction, the mixture was partitioned between 60 mL CH_2Cl_2 and 40 mL saturated aqueous $NaHCO_3$ solution. The aqueous phase was then extracted three times with 12.5 mL CH_2Cl_2 each. The combined organic layers were dried over $MgSO_4$ for 30 minutes, filtered, and concentrated in a rotary evaporator. Any remaining DMF was removed by repeated co-evaporation with CH_2Cl_2, followed by overnight drying under high vacuum. Purification of the crude product was accomplished by column chromatography on silica gel (SiO_2 60, column: 5 cm × 7 cm; CH_2Cl_2/MeOH, 9:1 v/v), affording the title compound as an off-white solid after evaporation of the main fractions and drying under high vacuum (1.0541 g, 72 %).

R_f 0.82 (SiO_2 60, CH_2Cl_2/MeOH, 9:1; v/v).
UV/VIS (MeOH): λ_{max}: 259 nm (ε=15241.8 L·mol^{-1}·cm^{-1}).
logP_{OW} (ALOGPS 3.01): 4.62 ± 0.74.
^{1}H NMR (500 MHz, DMSO-d$_6$, 30 °C) δ 8.34 (s, 1H, H-C(2), (1R)), 8.33 (s, 1H, H-C(2), (1S)), 8.15 (s, 1H, H-C(8)), 7.28 (s, 2H, NH_2), 6.14 (d, J = 2.0 Hz, 1H, H-C(1′), (1R) + (1S)), 5.37 (dd, J = 2.5, 6.0 Hz, 1H, H-C(2′), (1R) + (1S)), 5.12 (t, J = 5.5 Hz, 1H, HO-C(5′), (1R)), 5.10 (t, J = 5.5 Hz, 1H, HO-C(5′), (1S)), 4.98 (dd, J = 2.5, 5.5 Hz, 1H, H-C(3′), (1R) + (1S)), 4.22–4.19 (m, 1H, H-C(4′), (1R) + (1S)), 3.55–3.54 (m, 2H, H_2-C(5′), (1R) + (1S)), 1.78–1.70 (m, 2H, H-C(a1″), H-C(b1″), (1R)), 1.61–1.57 (m, 2H, H-C(b1″), H-C(a1″), (1S)), 1.48–1.39 (m, 1H, H-C(a2″), (1R)), 1.36–1.17 (m, 23H, H-C(b2″), H_2-C(a3″)–H_2-C(a12″), (1R), H_2-C(b3″)–H_2-C(b12″), (1S)), 0.96 (t, J = 7.5 Hz, 3H, H_3-C(a2″), (1S)), 0.81 (t, J = 7.5 Hz, 3H, H_3-C(b2″), (1R)), 0.84 (t, J = 6.0 Hz, 3H, H_3-C(13″), (1R) + (1S)).
^{13}C NMR (126 MHz, DMSO-d$_6$, 30 °C) δ 156.22 (C(6), (1R)), 156.20 (C(6), (1S)), 152.86 (C(2), (1R) + (1S)), 149.10 (C(4), (1S)), 149.04 (C(4), (1R)), 140.06 (C(8), (1R)), 139.93 (C(8), (1S)), 119.19 (C(5), (1R)), 119.14 (C(5), (1S)), 117.38 (C(acetal), (1R)), 117.27 (C(acetal), (1S)), 89.87 (C(1′), (1R)), 89.81 (C(1′), (1S)), 87.11 (C(4′), (1S)), 87.08 (C(4′), (1R)), 83.97 (C(2′), (1S)), 83.90 (C(2′), (1R)), 81.79 (C(3′), (1R)), 81.77 (C(3′), (1S)), 61.83 (C(5′), (1R)), 61.79 (C(5′), (1S)), 36.26 (C(a1″), (1R)), 36.07 (C(b1″), (1S)), 31.47 (C(a3″), (1R), C(b3″), (1S)), 29.45–28.79 (7 × C, C(a4″)–C(a10″), (1R)), 29.45–

28.79 (7 × C, C(b4″)–C(b10″), (1S)), 23.77 (C(a2″), (1R)), 23.51 (C(b2″), (1S)), 22.26 (C(a12″), (1R), C(b12″), (1S)), 14.05 (C(b2″), (1R), C(a2″), (1S)), 8.48 (C(b13″), (1S)), 7.88 (C(a13″), (1R)).
ESI – MS: m/z 490.32 $[M+H]^+$ (calcd for $C_{26}H_{43}N_5O_4$: 489.65).
Elemental analysis calcd (%) for $C_{26}H_{43}N_5O_4 \cdot 0.25\ H_2O$: C 63.61, H 9.109, N 13.69; found: C 63.20, H 8.87, N 14.17.

((2R/2S,3aR,4R,6R,6aR)-6-(6-amino-9H-purin-9-yl)-2-methyl-2-heptadecyltetrahydrofuro[3,4-d][1,3]dioxol-4-yl)methanol **(2j, NL_5.3₂.0.0, diastereoisomeric mixture, (1R) + (1S))**

Dry adenosine (1, NS_5.0.0.0; 0.5 g, 1.87 mmol) was dissolved in 8.5 mL of dry, amine-free dimethylformamide. Following dissolution, nonadecan-2-one (0.56 g, 2 mmol), triethyl orthoformate (0.35 mL), and 4 M HCl in 1,4-dioxane (3 mL) were added to the mixture. The resulting solution was stirred at room temperature for 24 hours. Upon completion of the reaction, the mixture was partitioned between 60 mL CH_2Cl_2 and 40 mL saturated aqueous $NaHCO_3$ solution. The aqueous phase was extracted three times with 12.5 mL CH_2Cl_2 each, and the organic layer was subsequently washed twice with 50 mL H_2O. The combined organic layers were dried over MgSO4 for 30 minutes, filtered, and concentrated using a rotary evaporator. Any residual DMF was removed via repeated co-evaporation with CH_2Cl_2, followed by overnight drying under high vacuum. Purification by column chromatography on silica gel (SiO_2 60, column: 5 cm × 7 cm; CH_2Cl_2/MeOH, 9:1 v/v) afforded the title compound as a white solid (0.818 g, 82 %) after evaporation of the main fractions and final high vacuum drying.

R_f 0.84 (SiO_2 60, CH_2Cl_2/MeOH 9:1; v/v).
UV/Vis (MeOH): λ_{max}: 260 nm (ε=12434.8 $L \cdot mol^{-1} \cdot cm^{-1}$).
logP_{OW} (ALOGPS 3.01): 5.18 ± 0.74.
^{1}H NMR (500 MHz, DMSO-d_6, 30 °C) δ 8.33 (s, 1H, H-C(2)), 8.15 (s, 1H, H-C(8)), 7.28 (s, 2H, NH_2), 6.14 (d, *J* = 3.0 Hz, 1H, H-C(1′), (1S)), 6.11 (d, *J* = 3.0 Hz, 1H, H-C(1′), (1R)), 5.37 (dd, *J* = 3.0, 6.5 Hz, 1H, H-C(2′), (1R)), 5.33 (dd, *J* = 3.0, 6.5 Hz, 1H, H-C(2′), (1S)), 5.17 (t, *J* = 5.5 Hz, 1H, HO-C(5′), (1S)), 5.14 (t, *J* = 5.5 Hz, 1H, HO-C(5′), (1R)), 4.98 (dd, *J* = 2.5, 6.0 Hz, 1H, H-C(3′), (1R)), 4.95 (dd, *J* = 2.5, 6.0 Hz, 1H, H-C(3′), (1S)), 4.23–4.18 (m, 1H, H-C(4′), (1R) + (1S)), 3.57–3.51 (m, 2H, H_2-C(5′), (1R) + (1S)), 1.75 (dt, *J* = 9.0 Hz, 2H, H_2C(a1″), (1R)), 1.57 (dt, *J* = 10.0 Hz, 2H, H_2C(a1″), (1S)), 1.50–1.40 (m, 3H, H_3C(acetal), (1S)), 1.36–1.29 (m, 3H, H_3C(acetal), (1R)), 1.28–1.17 (m, 30H, H_2-C(a2″)–H_2-C(a16″), (1R)), 1.28–1.17 (m, 30H, H_2-C(b2″)–H_2-C(b16″), (1S)), 0.85 (t, *J* = 5.0 Hz, 3H, H_3-C(17″), (1R) + (1S)).
^{13}C NMR (126 MHz, DMSO-d_6, 30 °C) δ 156.12 (C(6), (1R)), 156.14 (C(6), (1S)), 152.62 (C(2), (1R) + (1S)), 148.86 (C(4), (1R)), 148.80 (C(4), (1S)), 139.74 (C(8), (1S)), 139.63 (C(8), (1R)), 119.10 (C(5), (1S)), 119.05 (C(5), (1R)), 115.04 (C(acetal), (1S)), 114.66 (C(acetal), (1R)), 89.68 (C(1′), (1S)), 89.43 (C(1′), (1R)), 86.64 (C(4′), (1S)), 86.54 (C(4′), (1R)), 83.56 (C(2′), (1S)), 83.20 (C(2′), (1R)), 81.67 (C(3′), (1S)), 81.17 (C(3′), (1R)), 61.61 (C(5′), (1S)), 61.57 (C(5′), (1R)), 35.77 (C(a1″), (1R)), 35.73 (C(b1″), (1S)), 31.30 (C(a3″), (1R)), 30.76 (C(b3″), (1S)), 29.17–28.71 (11 × C, C(a4″)–C(a15″), (1R)), 29.17–28.71 (11 × C, C(b4″)–C(b15″), (1S)), 25.01 (C(b1″), (1R)), 23.90 (C(a1″), (1S)), 23.35 (C(a2″), (1R)), 23.28 (C(b2″), (1S)), 22.09 (C(a16″), (1R)), 22.04 (C(b16″), (1S)), 13.92 (C(17″), (1R) + (1S)).
ESI – MS: m/z 532.25 $[M+H]^+$ (calcd for $C_{29}H_{49}N_5O_4$: 531.73).
Elemental analysis calcd (%) for $C_{29}H_{49}N_5O_4 \cdot 0.6\ H_2O$: C 63.96; H 9.24; N 13.15; found: C 64.20, H 9.33, N 12.91.

ethyl 3-((2R/2S,3aR,4R,6R,6aR)-4-(6-amino-9H-purin-9-yl)-6-(hydroxymethyl)-2-methyltetrahydrofuro[3,4-d][1,3]dioxol-2-yl)propanoate **(3, NL_5.1.0.0, diastereoisomeric mixture, (1R) + (1S))** *(adapted from Frank Seela & Cramer, 1975)*

Dry adenosine (1, NS_5.0.0.0, 0.75 g; 2.71 mmol) was first dissolved in 10 mL of dry, amine-free DMF. Ethyl levulinate (0.77 mL, 5.43 mmol), orthoformic acid triethyl ester (0.89 mL, 5.43 mmol), and 2 mL of 4 M HCl in 1,4-dioxane were then added sequentially. After stirring the mixture at room temperature for 24 hours, the reaction was partitioned between 75 mL CH_2Cl_2 and 30 mL saturated aqueous sodium hydrogen carbonate. The aqueous phase was extracted twice with 25 mL CH_2Cl_2 each. The combined organic layers

were concentrated by rotary evaporation, and the oily residue was thoroughly evaporated several times with CH_2Cl_2 to remove remaining DMF. Precipitation in dry diethyl ether, followed by filtration and drying under high vacuum, afforded the product as a white solid (0.67 g, 71%).

R_f 0.65 (SiO_2 60, CH_2Cl_2/MeOH 9:1; v/v).

UV/Vis (MeOH): λ_{max}: 260 nm (ε=10455.7 l·mol^{-1}·cm^{-1}).

logP_{OW} (ALOGPS 3.01): 0.87 ± 0.74.

^{1}H NMR (500 MHz, DMSO-d$_6$) δ 8.38 (s, 1H, H-C(2)), 8.21 (s, 1H, H-C(8)), 7.42 (s, 2H, NH_2), 6.17 (d, *J* = 2.5 Hz, 1H, H-C(1′), (1R) + (1S)), 5.45 (dd, *J* = 2.5, 6.3 Hz, 1H, H-C(2′), (1R) + (1S)), 5.11 (t, *J* = 5.4 Hz, 1H, HO-C(5′), (1R) + (1S)), 5.00 (dd, *J* = 6.5, 2.4 Hz, 1H, H-C(3′), (1R) + (1S)), 4.25 (Ψq, *J* = 2.5, 5.0 Hz, 1H, H-C(4′), (1R) + (1S)), 4.09 (q, *J* = 7.1 Hz, 2H, H_2-C(4a″), (1R)), 4.05 (q, *J* = 7.0 Hz, 2H, H_2-C(4b″), (1S)), 3.55–3.47 (m, 2H, H_2-C(5′), (1R) + (1S)), 2.50 (t, *J* = 7.6 Hz, 2H, H_2-C(2a″), (1R)), 2.35 (t, *J* = 7.6 Hz, 2H, H_2-C(2b″), (1S)), 2.11 (t, *J* = 7.7 Hz, 2H, H_2-C(1a″), (1R)), 1.93 (t, *J* = 7.7 Hz, 2H, H_2-C(1b″), (1S)), 1.53 (s, 3H, H_3-C(1a″), (1S)), 1.33 (s, 3H, H_3-C(1b″), (1R)), 1.22 (t, *J* = 7.1 Hz, 3H, H_3-C(5a″), (1R)), 1.16 (t, *J* = 7.1 Hz, 3H, H_3-C(5b″), (1S)).

^{13}C NMR (126 MHz, DMSO-d$_6$) δ 172.52 (C(3a″), (1R)), 172.39 (C(3b″), (1S)), 156.79 (C(6), (1R) + (1S)), 152.23 (C(2), (1R) + (1S)), 148.77 (C(4), (1R) + (1S)), 139.75 (C(8), (1R)), 139.80 (C(8), (1S)), 119.01 (C(5), (1R) + (1S)), 114.11 (C(acetal), (1S)), 113.73 (C(acetal), (1R)), 89.66 (C(1′), (1S)), 89.40 (C(1′), (1R)), 86.74 (C(4′), (1S)), 86.58 (C(4′), (1R)), 83.82 (C(2′), (1S)), 83.39 (C(2′), (1R)), 81.83 (C(3′), (1S)), 81.30 (C(3′), (1R)), 61.51 (C(5′), (1S)), 61.43 (C(5′), (1R)), 59.85 (C(4a″), (1R)), 59.79 (C(4b″), (1S)), 33.43 (C(2a″), (1R)), 33.28 (C(2b″), (1S)), 28.96 (C(1b″), (1S)), 28.16 (C(1a″), (1R)), 24.87 (C(1a″), (1S)), 23.47 (C(1b″), (1R)), 13.99 (C(5a″), (1R)), 13.91 (C(5b″), (1S)).

ESI – MS: m/z 394.18 [M+H]$^+$ (calcd for $C_{17}H_{23}N_5O_6$: 393.39).

Elemental analysis calcd (%) for $C_{17}H_{23}N_5O_6$: C 51.94, H 5.86, N 17.91; found: C 51.90, H 5.89, N 17.80.

2.2.2. O-2′,3′-Nucleolipids of 1,N^6-Ethenodenosine

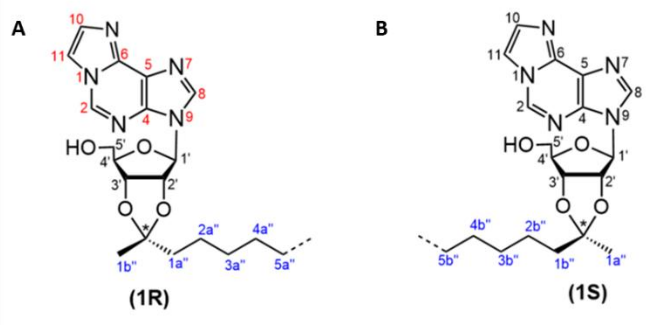

Supplementary Figure S4. Schematic illustration of the numbering of asymmetrical O-2′,3′-ketalized 1,N^6-ethenoadenosine (εAdo) derivatives. (**A**) general numbering scheme for 1R diastereoisomer. (**B**) general numbering scheme for 1S diastereoisomer.

((2R/2S,3aR,4R,6R,6aR)-6-(3H-imidazo[2,1-i]purin-3-yl)-2-methyl-2-pentyltetrahydrofuro[3,4-d][1,3]dioxol-4-yl)methanol Hydrochloride **(7f, εNL_5.4$_2$.0.0, diastereoisomeric mixture, (1R) + (1S))**

Synthesis pathway 1: Compound 2f (NL_5.4$_2$.0.0, 0.1817 g, 0.5 mmol) was dissolved in 10 mL of distilled chloroacetaldehyde solution (2–3 M). The pH of the reaction mixture was adjusted to 4–4.5 by adding 200 µl of ammonium bicarbonate solution (1 M). The mixture was stirred for 24 hours at 37 °C. The solvent was removed using a rotary evaporator (water bath not exceeding 35 °C), and the residue was repeatedly evaporated with ethanol to thoroughly eliminate any remaining aqueous chloroacetaldehyde solution. The crude product was dried overnight under high vacuum. The main product was purified via gradient column chromatography on silica gel (SiO_2 60, column: 6 cm x 5 cm; CH_2Cl_2/MeOH, 9:1, 87:13, 85:15, 8:2

v/v) and then freeze-dried from 1,4-dioxane. The isolated, solvent-free product was obtained as a white solid (0.142 g, 73 %).

Synthesis pathway 2: Anhydrous ethenoadenosine (6, εNS_5.0.0.0, 0.174 g, 0.6 mmol) was dissolved in 7 mL of dry, amine-free dimethylformamide. Heptan-2-one (0.0655 mL, 0.573 mmol), triethyl orthoformate (0.12 mL), and 4 M HCl in 1,4-dioxane (2 mL) were then added. The solution was stirred for 24 hours at room temperature. The reaction mixture was then partitioned between 80 mL of CH_2Cl_2 and 40 mL of saturated aqueous $NaHCO_3$. The aqueous phase was extracted three times with 15 mL CH_2Cl_2 each, until no fluorescence remained. The combined organic phases were dried over $MgSO_4$ for 1 hour. The filtered crude product was concentrated using a rotary evaporator and dried overnight under high vacuum. The main product was purified by column chromatography on silica gel (SiO_2 60, column: 7 cm x 5 cm; CH_2Cl_2/MeOH, 93:7 v/v) and freeze-dried from 1,4-dioxane. The isolated, solvent-free product was obtained as a white solid (0.086 g, 37 %).

R_f 0.68 (SiO_2 60, CH_2Cl_2/MeOH 93:7; v/v).

UV/Vis (MeOH): λ_{max}: 229 nm (ε=32714.0 L·mol^{-1}·cm^{-1}).

Fluorescence (MeOH): λ_{Em}: 409 nm, λ_{Ex}: 235 nm.

logP_{OW} (ALOGPS 3.01): 1.74 ± 0.74.

^{1}H NMR (500 MHz, DMSO-d$_6$) δ 9.30 (s, 1H, H-C(2), (1R) + (1S)), 8.53 (s, 1H, H-C(8), (1R)), 8.52 (s, 1H, H-C(8), (1S)), 8.09 (d, *J* = 1.5 Hz, 1H, H-C(10), (1R) + (1S)), 7.57 (d, *J* = 1.5 Hz, 1H, H-C(11), (1R) + (1S)), 6.29 (d, *J* = 2.5 Hz, 1H, H-C(1′), (1S)), 6.27 (d, *J* = 2.5 Hz, 1H, H-C(1′), (1R)), 5.40 (dd, *J* = 3.0, 6.5 Hz, 1H, H-C(2′), (1R)), 5.37 (dd, *J* = 2.5, 6.0 Hz, 1H, H-C(2′), (1S)), 5.05 (t, *J* = 5.5 Hz, 1H, HO-C(5′), (1R) + (1S)), 5.02 (dd, *J* = 3.0, 6.5 Hz, 1H, H-C(3′), (1R)), 5.01 (dd, *J* = 3.0, 6.5 Hz, 1H, H-C(3′), (1S)), 4.26–4.23 (m, 1H, H-C(4′), (1R) + (1S)), 3.55–3.53 (m, 2H, H_2-C(5′), (1R) + (1S)), 1.79–1.76 (m, 2H, H_2-C(1a″), (1R)), 1.61–1.58 (m, 2H, H_2-C(1b″), (1S)), 1.52 (s, 3H, H_3-C(1a″), (1S)), 1.36–1.24 (m, H_3-C(1b″), H_2-C(2a″)–H_2-C(4a″), (1R), H_2-C(2b″)–H_2-C(4b″), (1S)), 0.91 (t, *J* = 7.0 Hz, 3H, H_3-C(5a″), (1R)), 0.86 (t, *J* = 7.0 Hz, 3H, H_3-C(5b″), (1S)).

^{13}C NMR (126 MHz, DMSO-d$_6$) δ 140.39 (C(6), (1R) + (1S)), 139.91 (C(8), (1S)), 139.87 (C(8), (1R)), 137.90 (C(2), (1R) + (1S)), 137.08 (C(4), (1R) + (1S)), 132.74 (C(10), (1R) + (1S)), 123.10 (C(5), (1R) + (1S)), 115.13 (C(acetal), (1S)), 114.73 (C(acetal), (1R)), 112.18 (C(11), (1R) + (1S)), 89.92 (C(1′), (1S)), 89.71 (C(1′), (1R)), 87.02 (C(4′), (1S)), 86.90 (C(4′), (1R)), 84.17 (C(2′), (1S)), 83.76 (C(2′), (1R)), 81.62 (C(3′), (1S)), 81.14 (C(3′), (1R)), 61.45 (C(5′), (1R) + (1S)), 38.93 (C(1a″), (1R)), 38.22 (C(1b″), (1S)), 31.28 (C(3a″), (1R), C(3b″), (1S)), 24.97 (C(1a″), (1S)), 23.45 (C(2b″), (1S)), 23.38 (C(2a″), (1R)), 22.82 (C(1b″), (1R)), 21.96 (C(4a″), (1R)), 21.92 (C(4b″), (1S)), 13.81 (C(5a″), (1R)), 13.77 (C(5b″), (1S)).

ESI – MS: m/z 388.20 $[M+H]^+$, 775.76 $[2M+H]^+$ (calcd for $C_{19}H_{25}N_5O_4$: 387.43).

Elemental analysis calcd (%) for $C_{19}H_{25}N_5O_4$ · 0.3 HCl · 0.05 H_2O · 0.1 Heptan-2-one: C 57.61, H 6.64, N 17.14; found: C 57.61, H 6.58, N 17.05.

((2R/2S,3aR,4R,6R,6aR)-6-(3H-imidazo[2,1-i]purin-3-yl)-2-methyl-2-nonyltetrahydrofuro[3,4-d][1,3]dioxol-4-yl)methanol Hydrochloride **(7g, εNL_5.5₂.0.0, diastereoisomeric mixture, (1R) + (1S))**

Synthesis pathway 1: Compound 2g (NL_5.5$_2$.0.0, 0.1018 g, 0.24 mmol) was dissolved in 10 mL of distilled chloroacetaldehyde solution (2–3 M). The pH of the reaction mixture was adjusted to 4–4.5 by adding 200 μL ammonium bicarbonate solution (1 M). The mixture was stirred for 24 hours at 37 °C. The solvent was removed using a rotary evaporator (water bath not exceeding 35 °C), and the residue was repeatedly evaporated with ethanol to thoroughly eliminate remaining aqueous chloroacetaldehyde. The crude product was dried overnight under high vacuum. The main product was purified by gradient column chromatography on silica gel (SiO_2 60, column: 6 cm x 5 cm; CH_2Cl_2/MeOH, 9:1, 87:13, 85:15, 8:2 v/v) and then freeze-dried from 1,4-dioxane. The isolated, solvent-free product was obtained as a white solid (0.062 g, 65 %).

Synthesis pathway 2: Anhydrous ethenoadenosine (6, εNS_5.0.0.0, 0.174 g, 0.6 mmol) was dissolved in 7 mL of dry, amine-free dimethylformamide. Heptan-2-one (0.0655 mL, 0.573 mmol), triethyl orthoformate (0.12 mL), and 4 M HCl in 1,4-dioxane (2 mL) were added. The solution was stirred for 24 hours at room temperature. The reaction mixture was then partitioned between 80 mL of CH_2Cl_2 and 40 mL of saturated aqueous $NaHCO_3$. The aqueous phase was extracted three times with 15 mL CH_2Cl_2 each, until no fluorescence remained. The combined organic phases were dried over MgSO4 for 1 hour. The filtered crude product was concentrated using a rotary evaporator and dried overnight under high vacuum. The main product was purified by column chromatography on silica gel (SiO_2 60, column: 7 cm x 5 cm; CH_2Cl_2/MeOH, 93:7 v/v) and freeze-dried from 1,4-dioxane. The isolated, solvent-free product was obtained as a white solid (0.039 g, 41 %).

R_f 0.68 (SiO_2 60, CH_2Cl_2/MeOH 93:7; v/v).

UV/Vis (MeOH): λ_{max}: 229 nm (ε=32714.0 L·mol^{-1}·cm^{-1}).

Fluorescence (MeOH): λ_{Em}: 409 nm, λ_{Ex}: 235 nm.

logP_{OW} (ALOGPS 3.01): 3.36 ± 0.74.

^{1}H NMR (500 MHz, DMSO-d$_6$) δ 9.30 (s, 1H, H-C(2), (1R) + (1S)), 8.52 (s, 1H, H-C(8), (1R) + (1S)), 8.09 (s, 1H, H-C(10), (1R) + (1S)), 7.57 (s, 1H, H-C(11), (1R) + (1S)), 6.29 (d, *J* = 2.5 Hz, 1H, H-C(1′), (1R)), 6.27 (d, *J* = 2.5 Hz, 1H, H-C(1′), (1S)), 5.40 (dd, *J* = 2.5, 6.5 Hz, 1H, H-C(2′), (1S)), 5.36 (dd, *J* = 3.0, 6.5 Hz, 1H, H-C(2′), (1R)), 5.05 (t, *J* = 5.0 Hz, 1H, HO-C(5′), (1R) + (1S)), 5.01 (dd, *J* = 2.5, 6.5 Hz, 1H, H-C(3′), (1S)), 4.98 (dd, *J* = 2.5, 6.0 Hz, 1H, H-C(3′), (1R)), 4.26–4.24 (m, 1H, H-C(4′), (1R) + (1S)), 3.56–3.52 (m, 2H, H$_2$-C(5′), (1R) + (1S)), 1.79–1.76 (m, 2H, H$_2$-C(1a″), (1R)), 1.61–1.58 (m, 2H, H$_2$-C(1b″), (1S)), 1.51 (s, 3H, H$_3$-C(1a″), (1S)), 1.49–1.23 (m, 17H, H$_3$-C(1b″), H$_2$-C(2a″)–H$_2$-C(8a″), (1R), H$_2$-C(2b″)–H$_2$-C(8b″), (1S)), 0.87–0.82 (m, 6H, H$_3$-C(9a″), H$_3$-C(9b″), (1R) + (1S)).

^{13}C NMR (126 MHz, DMSO-d$_6$) δ 140.33 (C(6), (1R) + (1S)), 139.87 (C(8), (1S)), 139.82 (C(8), (1R)), 137.84 (C(2), (1R) + (1S)), 137.02 (C(4), (1R) + (1S)), 132.68 (C(10), (1R) + (1S)), 123.06 (C(5), (1R) + (1S)), 115.09 (C(acetal), (1R)), 114.68 (C(acetal), (1S)), 112.13 (C(11), (1R) + (1S)), 89.85 (C(1′), (1R)), 89.66 (C(1′), (1S)), 86.96 (C(4′), (1R)), 86.85 (C(4′), (1S)), 84.12 (C(2′), (1R)), 83.68 (C(2′), (1S)), 81.55 (C(3′), (1R)), 81.07 (C(3′), (1S)), 61.39 (C(5′), (1R) + (1S)), 38.97 (C(1b″), (1S)), 38.19 (C(1a″), (1R)), 31.16 (C(3b″), (1S)), 31.12 (C(3a″), (1R)), 29.02–28.52 (8 × C, C(4a″)–C(7a″), (1R), C(4b″)–C(7b″), (1S)), 24.92 (C(1b″), (1R)), 23.73 (C(2b″), (1S)), 23.32 (C(1a″), (1S)), 23.13 (C(2a″), (1R)), 21.95 (C(8b″), (1S)), 21.93 (C(8a″), (1R)), 13.80 (C(9a″), (1R)), 13.77 (C(9b″), (1S)).

ESI – MS: m/z 444.26 [M+H]$^+$, 887.51 [2M+H]$^+$ (calcd for $C_{23}H_{33}N_5O_4$: 443.54).

Elemental analysis calcd (%) for $C_{23}H_{33}N_5O_4$ · 3 HCl: C 50.36, H 6.13, N 12.64; found: C 49.96, H 6.56, N 12.67.

((2R/2S,3aR,4R,6R,6aR)-6-(3H-imidazo[2,1-i]purin-3-yl)-2-methyl-2-tridecyltetrahydrofuro[3,4-d][1,3]dioxol-4-yl)methanol Hydrochloride **(7h, εNL_5.7$_2$.0.0, diastereoisomeric mixture, (1R) + (1S))**

Synthesis pathway 1: Anhydrous compound 2h (NL_5.7$_2$.0.0, 0.1154 g, 0.24 mmol) was dissolved in 10 mL of distilled chloroacetaldehyde solution (2–3 M). The pH of the reaction mixture was adjusted to 4–4.5 by adding 200 µL of ammonium hydrogen carbonate solution (1 M). The mixture was stirred for 24 hours at 37 °C. The solvent was removed using a rotary evaporator (water bath not exceeding 35 °C), and the residue was repeatedly evaporated with ethanol to ensure complete removal of aqueous chloroacetaldehyde. The crude product was dried overnight under high vacuum. The main product was purified by column chromatography on silica gel (SiO_2 60, column: 8 cm x 5 cm; CH_2Cl_2/MeOH, 9:1 v/v). The isolated, solvent-free product was obtained as a white solid (0.072 g, 60 %) under high vacuum.

Synthesis pathway 2: Dry ethenoadenosine (6, εNS_5.0.0.0, 0.2002 g, 0.69 mmol) was dissolved in 8 mL of dry, amine-free dimethylformamide and 1 mL of distilled CH_2Cl_2. Pentadecan-2-one (0.1573 g, 0.7 mmol), triethyl orthoformate (0.16 mL), and 4 M HCl in 1,4-dioxane (3 mL) were then added. The solution was stirred for 24 hours at room temperature. The reaction mixture was then partitioned between 60 mL of CH_2Cl_2 and 40 mL of saturated aqueous $NaHCO_3$. The aqueous phase was extracted with CH_2Cl_2 until

fluorescence disappeared. The combined organic phases were washed with 100 mL of H_2O and dried over $MgSO_4$ for 1 hour. The filtered crude product was concentrated using a rotary evaporator. The remaining DMF was removed at 30 °C using a Kugelrohr tube apparatus. The main product was purified by column chromatography on silica gel (SiO_2 60, column: 9.0 cm x 5 cm; CH_2Cl_2/MeOH, 9:1 v/v). The isolated, solvent-free product was obtained as a white solid under high vacuum (0.1378 g, 40 %).

R_f 0.76 (SiO_2 60, CH_2Cl_2/MeOH 9:1; v/v).

UV/Vis (MeOH): λ_{max}: 229 nm (ε=30843.8 L·mol^{-1}·cm^{-1}).

Fluorescence (MeOH): λ_{Em}: 408 nm, λ_{Ex}: 235 nm.

logP_{OW} (ALOGPS 3.01): 4.48 ± 0.74.

^{1}H NMR (500 MHz, DMSO-d$_6$) δ 9.30 (s, 1H, H-C(2), (1R) + (1S)), 8.53 (s, 1H, H-C(8), (1R) + (1S)), 8.09 (d, *J* = 1.5 Hz, 1H, H-C(10), (1R) + (1S)), 7.57 (d, *J* = 1.5 Hz, 1H, H-C(11), (1R) + (1S)), 6.29 (d, *J* = 2.5 Hz, 1H, H-C(1′), (1S)), 6.28 (d, *J* = 2.5 Hz, 1H, H-C(1′), (1R)), 5.40 (dd, *J* = 3.0, 6.5 Hz, 1H, H-C(2′), (1R)), 5.36 (dd, *J* = 2.5, 6.0 Hz, 1H, H-C(2′), (1S)), 5.05 (t, *J* = 5.0 Hz, 1H, HO-C(5′), (1R) + (1S)), 5.01 (dd, *J* = 3.0, 6.5 Hz, 1H, H-C(3′), (1R)), 4.98 (dd, *J* = 2.5, 6.0 Hz, 1H, H-C(3′), (1S)), 4.25–4.23 (m, 1H, H-C(4′), (1R) + (1S)), 3.58–3.50 (m, 2H, H_2-C(5′), (1R) + (1S)), 1.79–1.76 (m, 2H, H_2-C(1a″), (1R)), 1.61–1.44 (m, 5H, H_2-C(1b″), H_3-C(1a″), (1S)), 1.30–1.22 (m, 25H, H_3-C(1b″), H_2-C(2a″)–H_2-C(12a″), (1R), H_2-C(2b″)–H_2-C(12b″), (1S)), 0.86–0.83 (m, 6H, H_3-C(13a″), (1R), H_3-C(13b″), (1S)).

^{13}C NMR (126 MHz, DMSO-d$_6$) δ 140.36 (C(6), (1R) + (1S)), 139.87 (C(8), (1S)), 139.80 (C(8), (1R)), 137.86 (C(2), (1R) + (1S)), 137.01 (C(4), (1R) + (1S)), 132.73 (C(10), (1R) + (1S)), 123.10 (C(5), (1R) + (1S)), 115.09 (C(acetal), (1R)), 114.68 (C(acetal), (1S)), 112.11 (C(11), (1R) + (1S)), 89.86 (C(1′), (1S)), 89.69 (C(1′), (1R)), 86.98 (C(4′), (1S)), 86.86 (C(4′), (1R)), 84.14 (C(2′), (1S)), 83.72 (C(2′), (1R)), 81.57 (C(3′), (1S)), 81.08 (C(3′), (1R)), 61.41 (C(5′), (1R) + (1S)), 39.22 (C(1a″), (1R)), 38.20 (C(1b″), (1S)), 31.16 (C(11a″), (1R), C(11b″), (1S)), 29.03–28.57 (9 × C, C(2a″)–C(10a″), (1R), C(2b″)–C(10b″), (1S)), 23.33 (C(1b″), (1R)), 23.13 (C(1a″), (1S)), 21.95 (C(12a″), (1R), C(12b″), (1S)), 13.79 (C(13a″), (1R), C(13b″), (1S)).

ESI – MS: m/z 500.32 [M+H]$^+$, 999.64 [2M+H]$^+$ (calcd for $C_{27}H_{41}N_5O_4$: 499.65).

Elemental analysis calcd (%) for $C_{27}H_{41}N_5O_4$ · 0.3 HCl · 0.05 H_2O · 0.1 Pentadecan-2-one: C, 64.09, H, 8.33, N, 13.06; found: C 64.09, H 8.38, N 13.11.

((2R/2S,3aR,4R,6R,6aR)-6-(3H-imidazo[2,1-i]purin-3-yl)-2-ethyl-2-tridecyltetrahydrofuro[3,4-d][1,3]dioxol-4-yl)methanol Hydrochloride **(7i, εNL_5.8$_3$.0.0, diastereoisomeric mixture, (1R) + (1S))**

Synthesis pathway 1: Anhydrous compound 2i (NL_5.8$_3$.0.0, 0.130 g, 0.45 mmol) was dissolved in 10 mL of distilled chloroacetaldehyde solution (2–3 M). The pH of the mixture was adjusted to 4–4.5 by adding 200 µL of 1 M ammonium hydrogen carbonate solution. The reaction mixture was stirred for 24 hours at 37 °C. The solvent was removed via rotary evaporation (water bath below 35 °C), and the residue was co-evaporated with ethanol. The crude product was then partitioned between CH_2Cl_2 and aqueous $NaHCO_3$ solution. Extraction was repeated until the aqueous layer showed no further fluorescence. The combined organic layers were dried over $MgSO_4$ (30 min), filtered, and concentrated using a rotary evaporator. The resulting residue was additionally dried overnight under high vacuum. Purification by column chromatography (SiO_2 60, column: 5.5 cm x 4.5 cm; CH_2Cl_2/MeOH, 9:1 v/v) yielded the target compound as a white solid (0.1109 g, 48 %).

Synthesis pathway 2: Anhydrous 1,N^6-ethenoadenosine (6, **ε**NS_5.0.0.0, 0.173 g, 0.594 mmol) was dissolved in 5 mL of dry, amine-free dimethylformamide. Hexadecan-3-one (0.168 g, 0.7 mmol), triethyl orthoformate (0.12 mL), and 4 M HCl in 1,4-dioxane (1.5 mL) were then added. The mixture was stirred for 24 hours at room temperature. The reaction mixture was then partitioned between 60 mL of CH_2Cl_2 and 40 mL of saturated aqueous $NaHCO_3$ solution. The aqueous phase was extracted repeatedly with CH2Cl2 until no fluorescence remained. The combined organic layers were concentrated via rotary evaporation, and any residual DMF was removed using a Kugelrohr tube apparatus at 30 °C. The crude product was purified by

column chromatography (SiO_2 60, column: 5.5 cm x 4.5 cm; CH_2Cl_2/MeOH, 9:1 v/v), yielding the title compound as a white solid after drying under high vacuum (0.1067 g, 35 %).

R_f 0.78 (SiO_2 60, CH_2Cl_2/MeOH 9:1; v/v).

UV/Vis (MeOH): λ_{max}: 229 nm (ε=36445.9 L·mol^{-1}·cm^{-1}).

Fluorescence (MeOH): λ_{Em}: 408 nm, λ_{Ex}: 235 nm.

logP_{OW} (ALOGPS 3.01): 4.62 ± 0.74.

^{1}H NMR (500 MHz, DMSO-d$_6$, 30 °C) δ 9.30 (s, 1H, H-C(2), (1R)), 9.30 (s, 1H, H-C(2), (1S)), 8.53 (s, 1H, H-C(8), (1R)), 8.53 (s, 1H, H-C(8), (1S)), 8.09 (d, *J* = 1.0 Hz, 1H, H-C(10), (1R) + (1S)), 7.56 (d, *J* = 1.5 Hz, 1H, H-C(11), (1R) + (1S)), 6.29 (d, *J* = 2.0 Hz, 1H, H-C(1′), (1R) + (1S)), 5.40 (dd, *J* = 2.5, 6.0 Hz, 1H, H-C(2′), (1R) + (1S)), 5.04 (t, *J* = 5.0 Hz, 1H, HO-C(5′), (1R) + (1S)), 5.01–4.99 (m, 1H, H-C(3′), (1R) + (1S)), 4.27–4.23 (m, 1H, H-C(4′), (1R) + (1S)), 3.57–3.49 (m, 2H, H_2-C(5′), (1R) + (1S)), 1.79–1.72 (m, 2H, H_2-C(1a″), H_2-C(1b″), (1R)), 1.63–1.56 (m, 2H, H_2-C(1b″), H_2-C(1a″), (1S)), 1.46–1.43 (m, 2H, H_2-C(2b″), (1S)), 1.34–1.29 (m, 2H, H_2-C(2a″), (1R)), 1.28–1.17 (m, 23H, H_2-C(3a″)–H_2-C(12a″), (1R), H_2-C(3b″)–H_2-C(12b″), (1S)), 0.98 (t, *J* = 7.5 Hz, 3H, H_3-C(2b″), (1R)), 0.85 (t, *J* = 3.5 Hz, 3H, H_3-C(a2″), (1S)), 0.84 (t, *J* = 2.5 Hz, 3H, H_3-C(13a″), (1R)), 0.82 (t, *J* = 2.5 Hz, 3H, H_3-C(13b″), (1S)).

^{13}C NMR (126 MHz, DMSO-d$_6$, 30 °C) δ 140.37 (C(6), (1R) + (1S)), 139.89 (C(8), (1R)), 139.86 (C(8), (1S)), 137.86 (C(4), (1R) + (1S)), 137.02 (C(2), (1R)), 137.00 (C(2), (1S)), 132.73 (C(10), (1R) + (1S)), 123.10 (C(5), (1R) + (1S)), 117.02 (C(acetal), (1S)), 116.92 (C(acetal), (1R)), 112.10 (C(11), (1R) + (1S)), 89.79 (C(1′), (1R) + (1S)), 87.23 (C(4′), (1S)), 87.15 (C(4′), (1R)), 84.19 (C(2′), (1S)), 84.16 (C(2′), (1R)), 81.54 (C(3′), (1R) + (1S)), 61.44 (C(5′), (1R) + (1S)), 36.05 (C(1a″), (1R)), 35.77 (C(1b″), (1S)), 31.16 (C(11a″), (1R), C(11b″), (1S)), 29.10–28.56 (8 × C, C(3a″)–C(10a″), (1R)), 29.10–28.56 (8 × C, C(3b″)–C(10b″), (1S)), 23.51 (C(2a″), (1R)), 22.82 (C(2b″), (1S)), 21.95 (C(12a″), (1R), C(12b″), (1S)), 13.78 (C(2b″), (1R), C(2a″), (1S)), 8.23 (C(13b″), (1S)), 7.60 (C(13a″), (1R)).

ESI – MS: m/z 514.25 $[M+H]^+$, 1027.51 $[2M+H]^+$ (calcd for $C_{28}H_{43}N_5O_4$: 513.67).

Elemental analysis calcd(%) for $C_{28}H_{43}N_5O_4$ · 0.2 HCl · 0.05 H_2O · 0.05 Hexadecan-3-one: C 64.75, H 8.39, N 13.20; found: C 64.79, H 8.48, N 13.12.

((2R/2S,3aR,4R,6R,6aR)-6-(3H-imidazo[2,1-i]purin-3-yl)-2-methyl-2-heptadecyl tetrahydrofuro[3,4-d][1,3]dioxol-4-yl)methanol Hydrochloride **(7j, εNL_5.3$_2$.0.0, diastereoisomeric mixture, (1R) + (1S))**

Synthesis pathway 1: Anhydrous compound 2j (NL_5.3$_2$.0.0, 0.15 g, 0.306 mmol) was dissolved in 13 mL of distilled chloroacetaldehyde solution (2–3 M). The pH of the solution was adjusted to 4–4.5 by adding 200 μL of 1 M ammonium hydrogen carbonate solution. The mixture was stirred for 24 hours at room temperature, and the solvent was removed using a rotary evaporator (water bath below 35 °C). The residue was co-evaporated with ethanol and partitioned between 60 mL CH_2Cl_2 and 40 mL saturated aqueous NaHCO3. Extraction of the aqueous phase was repeated until no fluorescence remained. The combined organic layers were dried over $MgSO_4$ (30 min), filtered, concentrated by rotary evaporation, and dried under high vacuum overnight. Purification by column chromatography (SiO_2 60, column: 5.5 cm x 5 cm; CH_2Cl_2/MeOH, 9:1 v/v) afforded the main product as a white solid (0.0799 g, 47 %).

Synthesis pathway 2: Dry ethenoadenosine (6, εNS_5.0.0.0, 0.2008 g, 0.687 mmol) was dissolved in 8 mL of dry, amine-free dimethylformamide. 2-Nonadecanone (0.1962 g, 0.69 mmol), triethyl orthoformate (0.16 mL), dichloromethane (0.5 mL), and 4 M HCl in 1,4-dioxane (3 mL) were then added. The mixture was stirred for 24 hours at room temperature. The reaction mixture was then partitioned between 60 mL of CH2Cl2 and 40 mL of saturated aqueous $NaHCO_3$. The aqueous phase was extracted with CH_2Cl_2 until no fluorescence remained. The combined organic layers were washed with 60 mL H_2O and dried over $MgSO_4$ for 1 hour. The filtered crude product was concentrated using a rotary evaporator. The remaining DMF was removed at 30 °C using a Kugelrohr tube apparatus. Purification by column chromatography on silica gel (SiO_2 60, column: 8 cm x 5 cm; CH_2Cl_2/MeOH, 9:1 v/v) yielded the isolated, solvent-free product as a white solid under high vacuum (0.0991 g, 26 %).

R_f 0.81 (SiO_2 60, CH_2Cl_2/MeOH 9:1; v/v).

UV/Vis (MeOH): λ_{max}: 229 nm (ε=32046.5 L·mol^{-1}·cm^{-1}).

Fluorescence (MeOH): λ_{Em}: 409 nm, λ_{Ex}: 235 nm.
logP_{OW} (ALOGPS 3.01): 5.44 ± 0.74.
^{1}H NMR (500 MHz, DMSO-d_6, 30 °C) δ 9.31 (s, 1H, H-C(2), (1R) + (1S)), 8.53 (s, 1H, H-C(8), (1R) + (1S)), 8.10 (d, *J* = 1.0 Hz, 1H, H-C(10), (1R) + (1S)), 7.57 (d, *J* = 1.5 Hz, 1H, H-C(11), (1R) + (1S)), 6.27 (d, *J* = 2.5 Hz, 1H, H-C(1′), (1R) + (1S)), 5.41 (dd, *J* = 2.5, 6.5 Hz, 1H, H-C(2′), (1R)), 5.37 (dd, *J* = 5.0, 5.0 Hz, 1H, H-C(2′), (1S)), 5.06 (t, *J* = 5.0 Hz, 1H, HO-C(5′), (1R) + (1S)), 5.02 (dd, *J* = 2.5, 6.5 Hz, 1H, H-C(3′), (1R)), 4.99 (dd, *J* = 5.0, 5.0 Hz, 1H, H-C(3′), (1S)), 4.26–4.24 (m, 1H, H-C(4′), (1R) + (1S)), 3.57–3.53 (m, 2H, H_2-C(5′), (1R) + (1S)), 1.80–1.76 (m, 2H, H-C(1a″), (1R)), 1.52–1.46 (m, 2H, H-C(1b″), (1S)), 1.35–1.22 (m, 33H, H_3-C(1b″), (1R), H_3-C(1a″), (1S), H_2-C(2a″)–H_2-C(16a″), (1R), H_2-C(2b″)–H_2-C(16b″), (1S)), 0.85 (t, *J* = 6.5 Hz, 6H, H_3-C(17a″), (1R), H_3-C(17b″), (1S)).
^{13}C NMR (126 MHz, DMSO-d_6, 30 °C) δ 140.37 (C(6), (1R) + (1S)), 139.88 (C(8), (1S)), 139.80 (C(8), (1R)), 137.86 (C(4), (1R)), 137.84 (C(4), (1S)), 137.01 (C(2), (1R) + (1S)), 132.72 (C(10), (1R) + (1S)), 123.10 (C(5), (1R) + (1S)), 115.09 (C(acetal), (1S)), 114.67 (C(acetal), (1R)), 112.11 (C(11), (1R) + (1S)), 89.86 (C(1′), (1S)), 89.69 (C(1′), (1R)), 86.98 (C(4′), (1S)), 86.86 (C(4′), (1R)), 84.15 (C(2′), (1S)), 83.73 (C(2′), (1R)), 81.57 (C(3′), (1S)), 81.09 (C(3′), (1R)), 61.53 (C(5′), (1S)), 61.41 (C(5′), (1R)), 35.66 (C(1a″), (1R), C(1b″), (1S)), 31.16 (C(3a″), (1R)), 30.04 (C(3b″), (1S)), 29.04–28.56 (11 × C, C(4a″)–C(15a″), (1R)), 29.04–28.56 (11 × C, C(4b″)–C(15b″), (1S)), 24.93 (C(1b″), (1R)), 23.75 (C(1a″), (1S)), 23.33 (C(2a″), (1R)), 23.14 (C(2b″), (1S)), 21.96 (C(16a″), (1R), C(16b″), (1S)), 13.79 (C(17a″), (1R), C(17b″), (1S)).
ESI – MS: m/z 556.26 $[M+H]^+$, 1111.60 $[2M+H]^+$ (calcd for $C_{31}H_{49}N_5O_4$: 555.75).
Elemental analysis calcd (%) for $C_{31}H_{49}N_5O_4 \cdot 0.05\ HCl \cdot 0.65\ H_2O$: C 65.58, H 9.09, N 12.12; found: C 65.40, H 8.91, N 12.30.

ethyl 3-((2R/2S,3aR,4R,6R,6aR)-4-(hydroxymethyl)-6-(3H-imidazo[2,1-i]purin-3-yl)-2-methyltetrahydrofuro[3,4-d][1,3]dioxol-2-yl)propanoate Hydrochloride **(8, εNL_5.1.0.0, diastereoisomeric mixture, (1R) + (1S))**

Synthesis pathway 1: Anhydrous compound 5 (NL_5.1.0.0, 0.318 g, 0.5 mmol) was dissolved in 10 mL of distilled chloroacetaldehyde solution (2–3 M) and 10 mL of ethanol. The pH was adjusted to 4–4.5 by adding 200 µL of 1 M ammonium hydrogen carbonate solution. The reaction mixture was stirred for 24 hours at 37 °C. The solvent was removed using a rotary evaporator (water bath below 35 °C), and the residue was co-evaporated and dried overnight under high vacuum. The crude product was purified by column chromatography (SiO_2 60, column: 6 cm x 5 cm; CH_2Cl_2/MeOH, 9:1 v/v), yielding the main product as a white solid (0.1398 g, 67 %).

Synthesis pathway 2: Dry ethenoadenosine (6, εNS_5.0.0.0, 0.2006 g, 0.687 mmol) was dissolved in 7 mL of dry, amine-free dimethylformamide. Ethyl levulinate (0.1006 mL, 0.69 mmol), triethyl orthoformate (0.12 mL), dichloromethane (1 mL), and 4 M HCl in 1,4-dioxane (2 mL) were then added. The mixture was stirred for 24 hours at room temperature. The reaction mixture was then partitioned between 60 mL of CH2Cl2 and 40 mL of saturated aqueous $NaHCO_3$. The aqueous phase was extracted with CH_2Cl_2 until no fluorescence remained. The combined organic layers were washed with 60 mL H_2O and dried over $MgSO_4$ for 1 hour. The filtered crude product was concentrated using a rotary evaporator and dried overnight under high vacuum. Purification by column chromatography on silica gel (SiO_2 60, column: 6 cm x 5 cm; CH_2Cl_2/MeOH, 9:1 v/v) yielded the isolated, solvent-free product as a white solid under high vacuum (0.0991 g, 47 %).

R_f 0.59 (SiO_2 60 CH_2Cl_2/MeOH 9:1; v/v).
UV/Vis (MeOH): λ_{max}: 229 nm (ε=28063.8 L·mol^{-1}·cm^{-1}).
Fluorescence (MeOH): λ_{Em}: 409 nm, λ_{Ex}: 234 nm.
logP_{OW} (ALOGPS 3.01): 0.59 ± 0.74.
^{1}H NMR (500 MHz, DMSO-d_6) δ 9.30 (s, 1H, H-C(2), (1R) + (1S)), 8.51 (s, 1H, H-C(8), (1R) + (1S)), 8.09 (d, *J* = 1.5 Hz, 1H, H-C(10), (1R) + (1S)), 7.57 (d, *J* = 1.5 Hz, 1H, H-C(11), (1R) + (1S)), 6.29 (d, *J* = 2.5 Hz, 1H, H-C(1′), (1R) + (1S)), 5.45 (dd, *J* = 2.5, 6.5 Hz, 1H, H-C(2′), (1R)), 5.39 (dd, *J* = 3.0, 7.5 Hz, 1H, H-C(2′), (1S)), 5.06–5.02 (m, 2H, H-C(3′), HO-

C(5′), (1R) + (1S)), 4.33–4.24 (m, 1H, H-C(4′), (1R) + (1S)), 4.09 (q, J = 7.0 Hz, 2H, H_2-C(4a″), (1R), H_2-C(4b″), (1S)), 3.56–3.42 (m, 2H, H_2-C(5′), (1R) + (1S)), 2.47–2.31 (m, 2H, H_2-C(2a″), (1R), H_2-C(2b″), (1S)), 2.12–1.91 (m, 2H, H_2-C(1a″), (1R), H_2-C(1b″), (1S)), 1.53 (s, 3H, H_3-C(1a″), (1S)), 1.33 (s, 3H, H_3-C(1b″), (1R)), 1.21 (t, J = 7.0 Hz, 3H, H_3-C(5a″), (1R)), 1.06 (t, J = 7.0 Hz, 3H, H_3-C(5b″), (1S)).

^{13}C NMR (126 MHz, DMSO-d_6) δ 172.54 (C(3a″), (1R)), 172.45 (C(3b″), (1S)), 140.39 (C(6), (1R) + (1S)), 139.94 (C(8), (1S)), 139.85 (C(8), (1R)), 137.88 (C(2), (1R) + (1S)), 137.06 (C(4), (1R) + (1S)), 132.77 (C(10), (1R) + (1S)), 123.15 (C(5), (1R) + (1S)), 114.24 (C(acetal), (1S)), 113.81 (C(acetal), (1R)), 112.12 (C(11), (1R) + (1S)), 89.92 (C(1′), (1S)), 89.66 (C(1′), (1R)), 87.05 (C(4′), (1S)), 86.89 (C(4′), (1R)), 84.42 (C(2′), (1S)), 83.92 (C(2′), (1R)), 81.79 (C(3′), (1S)), 81.27 (C(3′), (1R)), 61.36 (C(5′), (1R) + (1S)), 59.84 (C(4a″), (1R), C(4b″), (1S)), 33.37 (C(1a″), (1R), C(1b″), (1S)), 28.96 (C(1a″), (1S)), 28.14 (C(1b″), (1R)), 24.93 (C(2b″), (1S)), 23.51 (C(2a″), (1R)), 13.98 (C(5a″), (1R), C(5b″), (1S)).

ESI – MS: m/z 418.17 $[M+H]^+$, 835.34 $[2M+H]^+$ (calcd for $C_{19}H_{23}N_5O_6$: 417.42).

Elemental analysis calcd (%) for $C_{19}H_{23}N_5O_6$ · 0.9 HCl · 0.05 H_2O · 0.2 Ethyl levulinate: C 51.05, H 5.53, N 14.50; found: C 51.05, H 5.54, N 14.59.

3. Cytotoxic Activity of Nucleolipid Derivatives

Table S1. Cytotoxicity of Nucleolipid Derivatives in BT4Ca, GOS-3, U87, U251, and PMA-Differentiated THP-1 Cells Determined by PrestoBlue™ Assay
Cytotoxicity values are expressed as percentage cytotoxicity (100 − viability [%]) relative to untreated control cells. Heatmap coloring indicates the degree of cytotoxicity relative to the negative control (equal DMSO concentration, 0.5%) and the reference compounds 5-FUrd and TMZ. Yellow to dark red colors represent increasing cytotoxicity compared to the negative control (25–50%, 50–75%, 75–90%, and >90%, respectively). Shades of green indicate enhanced cytotoxicity relative to 5-FUrd or TMZ (5–15%, 15–25%, 25–50%, and >50%, respectively). Statistical significance was calculated relative to the negative control ($p \leq 0.05$, $p \leq 0.01$, $p \leq 0.001$), relative to 5-FUrd (#$p \leq 0.05$, ##$p \leq 0.01$, ###$p \leq 0.001$), and relative to TMZ (°$p \leq 0.05$, °°$p \leq 0.01$, °°°$p \leq 0.001$). Values are given as mean ± SEM from n = 4 independent experiments performed in quadruplicate.

NL	Conc. [µM]	BT4Ca			GOS-3			U87			U251			THP-1			
		Cytotoxicity vs. Control [%]	Cytotoxicity vs. 5-FUrd [%]	P	Cytotoxicity vs. Control [%]	Cytotoxicity vs. 5-FUrd [%]	P	Cytotoxicity vs. Control [%]	Cytotoxicity vs. TMZ [%]	P	Cytotoxicity vs. Control [%]	Cytotoxicity vs. TMZ [%]	P	Cytotoxicity vs. Control [%]	Cytotoxicity vs. 5-FUrd [%]	Cytotoxicity vs. TMZ [%]	P
5-FUrd (NS_4.0.0.0)	1.56	67.9		**	27.8		***							-1.3			
	3.12	66.9		**	37.6		**							5.4			
	6.25	79.3		***	31.5		**							3.8			
	12.5	79.8		**	33.6		*							5.2			
	25	82.1		**	34.8		***							8.3			
	50	88.5		***	43.0		*							9.6			
TMZ	1.56							-8.8			-5.8			-7.8			
	3.12							-0.1			5.0			-1.1			
	6.25							-0.3			5.2			-6.4			
	12.5							5.2			3.4			-2.6			
	25							3.6			8.2			4.9			
	50							8.0			5.8			-0.7			
NL_5.4.0.0 (2a)	1.56																
	3.12	=			=												
	6.25		<			<								=	=	=	
	12.5																
	25	20.3			22.8												
	50	12.9			17.3												

NL	Conc. [μM]	BT4Ca			GOS-3			U87			U251			THP-1			
		Cytotoxicity vs. Control [%]	Cytotoxicity vs. 5-FUrd [%]	P	Cytotoxicity vs. Control [%]	Cytotoxicity vs. 5-FUrd [%]	P	Cytotoxicity vs. Control [%]	Cytotoxicity vs. TMZ [%]	P	Cytotoxicity vs. Control [%]	Cytotoxicity vs. TMZ [%]	P	Cytotoxicity vs. Control [%]	Cytotoxicity vs. 5-FUrd [%]	Cytotoxicity vs. TMZ [%]	P
NL_5.5.0.0 (2b)	1.56																
	3.12	=															
	6.25		<					=	=		=	=					
	12.5	23.8		**	=	<								=	=	=	
	25	35.3		***				17.2	13.6								
	50	90.7	2.3	**				87.0	79.0	*** °°°	49.9	44.1	*** °°°				
NL_5.7.0.0 (2c)	1.56																
	3.12	=						=	=		=	=					
	6.25		<		=	<								=	=	=	
	12.5	15.3		*				22.4	17.2	*** °	51.0	47.6	** °°				
	25	92.0	9.9	***	59.9	25.2	**	94.4	90.8	*** °°°	90.5	82.3	*** °°°				
	50	90.5	2.0	***	83.4	40.5	*** #	92.1	84.1	*** °°°	86.5	80.7	*** °°°	100.0	90.4	100.7	*** ### °°°
NL_5.9.0.0 (2d)	1.56																
	3.12	=			=	<		=	=		=	=					
	6.25		<								21.4	16.2	** °	=	=	=	
	12.5	24.2		*	40.2	6.5	**	74.3	69.1	* °	99.1	95.7	*** °°°				
	25	84.2	=	***	92.0	57.2	*** ###	98.2	94.6	*** °°°	99.0	90.8	*** °°°	100.0	91.6	95.1	*** ### °°°
	50	84.8		***	95.4	52.5	*** ##	96.8	88.8	*** °°°	97.3	91.5	*** °°°	95.4	85.9	96.1	*** ### °°°
NL_5.3.0.0 (2e)	1.56																
	3.12	=						=	=		=	=					
	6.25				=									=	=	=	
	12.5		<			<		32.8	27.6	*** °°	25.6	22.2	*** °°				
	25	47.7		*				75.1	71.5	*** °°°	80.1	71.9	*** °°°				
	50	71.5		**	24.5			87.2	79.2	*** °°°	88.5	82.6	*** °°°	70.9	61.3	71.6	* # °

NL	Conc. [µM]	BT4Ca			GOS-3			U87			U251			THP-1			
		Cytotoxicity vs. Control [%]	Cytotoxicity vs. 5-FUrd [%]	P	Cytotoxicity vs. Control [%]	Cytotoxicity vs. 5-FUrd [%]	P	Cytotoxicity vs. Control [%]	Cytotoxicity vs. TMZ [%]	P	Cytotoxicity vs. Control [%]	Cytotoxicity vs. TMZ [%]	P	Cytotoxicity vs. Control [%]	Cytotoxicity vs. 5-FUrd [%]	Cytotoxicity vs. TMZ [%]	P
NL_5.4$_2$.0.0 (2f)	1.56																
	3.12	=															
	6.25		<		=	<								=	=	=	
	12.5																
	25	11.3															
	50	13.2															
NL_5.5$_2$.0.0 (2g)	1.56	13.4		*				=	=		10.4	16.2					
	3.12	16.5		*	=	<					21.5	16.5		=	=	=	
	6.25	24.7		*				18.5	18.7	** °°	19.5	14.3					
	12.5	64.4	<	***	40.3	6.6	***	24.9	19.7	* °	24.8	21.4	** °°	21.0	15.8	23.6	*** # °°
	25	67.5		***	38.4	=	***	45.7	42.1	*** °°°	41.3	33.2	*** °°	47.0	38.7	42.2	*** ## °°°
	50	67.4		***	40.1		***	65.8	57.8	*** °°°	76.1	70.3	** °°	47.1	37.5	47.8	*** ### °°°
NL_5.7$_2$.0.0 (2h)	1.56	=						=	=		=	=					
	3.12	19.7	<		=	<								=	=	=	
	6.25	42.8		*				32.0	32.3	** °°	19.1	13.9	** °				
	12.5	80.0		**	42.8	9.2	***	54.7	49.5	*** °°°	61.8	58.3	*** °°	31.4	26.2	34.0	** ## °°
	25	81.6	=	***	88.1	53.3	*** ###	92.5	88.9	*** °°°	96.5	88.3	*** °°°	51.7	43.4	46.8	** # °°
	50	88.7		***	95.9	52.9	*** #	92.6	84.6	*** °°°	94.1	88.3	*** °°°	100.0	90.4	100.6	*** ### °°°
NL_5.8$_2$.0.0 (2i)	1.56																
	3.12	=						=			=	=		=	=	=	
	6.25		<		=	<			=								
	12.5	60.9		*				15.3			25.9	22.4	*** °°°	20.1	14.9	22.7	
	25	83.0	=	**				67.5	63.9	*** °°°	85.2	77.1	*** °°°	92.3	83.9	87.4	*** ### °°°
	50	91.6		***	100.0	57.0	* #	92.1	84.1	*** °°°	89.4	83.6	*** °°°	98.5	88.9	99.2	*** ### °°°

NL	Conc. [μM]	BT4Ca			GOS-3			U87			U251			THP-1			
		Cytotoxicity vs. Control [%]	Cytotoxicity vs. 5-FUrd [%]	P	Cytotoxicity vs. Control [%]	Cytotoxicity vs. 5-FUrd [%]	P	Cytotoxicity vs. Control [%]	Cytotoxicity vs. TMZ [%]	P	Cytotoxicity vs. Control [%]	Cytotoxicity vs. TMZ [%]	P	Cytotoxicity vs. Control [%]	Cytotoxicity vs. 5-FUrd [%]	Cytotoxicity vs. TMZ [%]	P
NL_5.3$_2$.0.0 (2j)	1.56	=	<		=	<		=	=		=	=		=	=	=	
	3.12																
	6.25																
	12.5																
	25	32.2						23.7	20.1	*** °°	34.8	26.7	*** °°°				
	50	35.9		**				59.1	51.1	*** °°°	74.1	68.3	*** °°°				
NL_5.1.0.0 (3)	1.56	=	<		=	<								=	=	=	
	3.12																
	6.25																
	12.5																
	25	14.2															
	50	12.5															
NL_5.cycl7.0.0 (4)	1.56	=	<		=	<		=	=		=	=		=	=	=	
	3.12																
	6.25																
	12.5																
	25																
	50																
NL_5.cycl8.0.0 (5)	1.56	=	<		=	<								=	=	=	
	3.12																
	6.25																
	12.5																
	25	18.8															
	50	13.6															

NL	Conc. [µM]	BT4Ca			GOS-3			U87			U251			THP-1			
		Cytotoxicity vs. Control [%]	Cytotoxicity vs. 5-FUrd [%]	P	Cytotoxicity vs. Control [%]	Cytotoxicity vs. 5-FUrd [%]	P	Cytotoxicity vs. Control [%]	Cytotoxicity vs. TMZ [%]	P	Cytotoxicity vs. Control [%]	Cytotoxicity vs. TMZ [%]	P	Cytotoxicity vs. Control [%]	Cytotoxicity vs. 5-FUrd [%]	Cytotoxicity vs. TMZ [%]	P
	1.56																
	3.12																
εNL_5.4.0.0 (7a)	6.25	=	<		=	<								=	=	=	
	12.5																
	25																
	50																
	1.56				=												
	3.12	=												=	=	=	
εNL_5.5.0.0 (7b)	6.25		<		13.0	<		=	=		=	=					
	12.5	68.5		*	14.4									73.7	68.6	76.3	*** ### °°°
	25	94.2	12.2	***	56.3	21.5	** #	23.1	19.5	** °	21.5	13.4	** °	100.0	91.6	95.1	*** ### °°°
	50	97.7	13.8	***	100.0	57.0	** ##	55.7	47.7	*** °°°	68.6	62.8	** °°°	98.5	88.9	99.2	*** ### °°°
	1.56																
	3.12							=	=		=	=					
εNL_5.7.0.0 (7c)	6.25	=	<		=	<								=	=	=	
	12.5							18.5	13.3		22.7	19.3	* °				
	25	90.4	8.4	***	66.5	31.8	*** ##	90.8	87.2	*** °°°	90.3	82.1	*** °°°	51.5	43.2	46.6	** # °°
	50	89.2	0.8	***	82.2	39.2	*** #	89.1	81.1	*** °°°	91.3	85.4	*** °°°	100.0	90.4	100.7	*** ### °°°
	1.56							=	=		=	=					
	3.12	=	<		=	<											
εNL_5.9.0.0 (7d)	6.25							18.2	18.4		34.4	29.2	*** °°	=	=	=	
	12.5	80.1		***	32.6	=	**	95.1	89.9	*** °°°	98.0	94.6	*** °°°				
	25	76.9	=	**	92.9	58.2	*** ###	94.9	91.3	*** °°°	97.8	89.6	*** °°°	100.0	91.6	95.1	*** ### °°°
	50	78.3		***	89.3	46.4	*** ##	94.4	86.3	*** °°°	95.9	90.1	*** °°°	100.0	90.4	100.7	*** ### °°°

NL	Conc. [µM]	BT4Ca			GOS-3			U87			U251			THP-1			
		Cytotoxicity vs. Control [%]	Cytotoxicity vs. 5-FUrd [%]	P	Cytotoxicity vs. Control [%]	Cytotoxicity vs. 5-FUrd [%]	P	Cytotoxicity vs. Control [%]	Cytotoxicity vs. TMZ [%]	P	Cytotoxicity vs. Control [%]	Cytotoxicity vs. TMZ [%]	P	Cytotoxicity vs. Control [%]	Cytotoxicity vs. 5-FUrd [%]	Cytotoxicity vs. TMZ [%]	P
εNL_5.3.0.0 (7e)	1.56	=	<		=	<		=	=		=	=		=	=	=	
	3.12																
	6.25																
	12.5	31.8		**				68.3	63.1	*** °°°	81.1	77.7	** °°	21.6	16.5	24.2	
	25	94.1	12.1	***	99.4	64.7	*** ###	92.6	89.0	*** °°°	90.1	81.9	*** °°°	96.5	88.2	91.7	*** ### °°°
	50	93.2	4.7	***	100.0	57.0	*** ##	90.7	82.7	*** °°°	92.0	86.1	*** °°°	98.4	88.8	99.1	*** ### °°°
εNL_5.4$_2$.0.0 (7f)	1.56	=	<		=	<								=	=	=	
	3.12																
	6.25																
	12.5				15.6												
	25				21.7												
	50				19.3												
εNL_5.5$_2$.0.0 (7g)	1.56	20.0	<		=	<		=	=		=	=		=	=	=	
	3.12	18.7															
	6.25	15.1															
	12.5	49.4		**	17.1			25.4	20.2	** °	20.3	16.8	** °°				
	25	65.7		***	41.9	7.2	***	66.8	63.2	*** °°°	77.1	68.9	*** °°°	27.5	19.2	22.7	* # °
	50	81.2	=	***	63.4	20.4	**	94.3	86.3	*** °°°	93.9	88.0	*** °°°	79.8	70.2	80.5	*** ### °°°
εNL_5.7$_2$.0.0 (7h)	1.56	22.1	<	*	11.8	<		12.0	20.7		10.6	16.4		=	=	=	
	3.12	36.9		*	16.7			20.7	20.8		19.5	14.5					
	6.25	50.7		**	27.5	=	***	33.1	33.4	* °	32.1	26.9	*** °°				
	12.5	84.6	4.8	***	58.5	24.9	***	83.0	77.8	** °°	88.2	84.7	*** °°°	20.0	14.8	22.6	
	25	93.6	11.6	***	94.5	59.7	*** ###	95.1	91.5	*** °°°	95.5	87.4	*** °°°	94.6	86.2	89.7	** ## °°
	50	91.0	2.6	***	89.7	46.8	*** #	93.9	85.9	*** °°°	93.4	87.5	*** °°°	100.0	90.5	100.7	*** ### °°°

NL	Conc. [μM]	BT4Ca			GOS-3			U87			U251			THP-1			
		Cytotoxicity vs. Control [%]	Cytotoxicity vs. 5-FUrd [%]	P	Cytotoxicity vs. Control [%]	Cytotoxicity vs. 5-FUrd [%]	P	Cytotoxicity vs. Control [%]	Cytotoxicity vs. TMZ [%]	P	Cytotoxicity vs. Control [%]	Cytotoxicity vs. TMZ [%]	P	Cytotoxicity vs. Control [%]	Cytotoxicity vs. 5-FUrd [%]	Cytotoxicity vs. TMZ [%]	P
εNL_5.8$_3$.0.0 (7i)	1.56				11.5												
	3.12	=	<		18.3	<		=	=		=	=		=	=	=	
	6.25				13.7												
	12.5	68.7		**	38.1	4.5	**	52.4	47.2	** °°°	59.0	55.6	*** °°°				
	25	90.5	8.4	***	77.8	43.1	** #	94.8	91.2	*** °°°	91.4	83.3	*** °°°	72.3	64.0	67.5	*** ### °°°
	50	91.1	2.6	***	85.3	42.3	*** #	91.4	83.4	*** °°°	88.1	82.2	*** °°°	100.0	90.4	100.6	*** ### °°°
εNL_5.3$_2$.0.0 (7j)	1.56				=												
	3.12	=			19.4	<		=	=		=	=					
	6.25				17.1									=	=	=	
	12.5	34.6	<	*	33.5	=	*	22.0	16.8	** °	33.4	30.0	* °				
	25	69.2		**	51.0	16.3	**	48.5	44.9	*** °°°	56.8	48.7	** °°	27.5	19.1	22.6	*** ### °°
	50	76.0		***	55.6	12.7	***	79.9	71.8	*** °°°	91.1	85.3	*** °°°	45.1	35.5	45.8	*** ### °°°
εNL_5.1.0.0 (8)	1.56																
	3.12	=															
	6.25																
	12.5	25.2	<		=	<								=	=	=	
	25	23.5															
	50	21.3															
εNL_5.cycl7.0.0 (9)	1.56																
	3.12	=	<		=	<		=	=		=	=					
	6.25				19.1			36.0	36.3	* °	46.5	41.4	** °°	=	=	=	
	12.5	71.1		**	35.0	1.4	**	85.0	79.7	*** °°°	91.0	87.6	*** °°°				
	25	79.7	=	**	91.1	56.4	*** ###	94.7	91.1	*** °°°	96.0	87.8	*** °°°	75.2	66.8	70.3	*** ## °°°
	50	79.2		***	95.0	52.1	*** ##	97.9	89.9	*** °°°	96.7	90.9	*** °°°	100.0	90.4	100.7	*** ### °°°

<table>
<tr><th>NL</th><th>Conc. [µM]</th><th colspan="3">BT4Ca</th><th colspan="3">GOS-3</th><th colspan="3">U87</th><th colspan="3">U251</th><th colspan="4">THP-1</th></tr>
<tr><th></th><th></th><th>Cytotoxicity vs. Control [%]</th><th>Cytotoxicit y vs. 5-FUrd [%]</th><th>P</th><th>Cytotoxicit y vs. Control [%]</th><th>Cytotoxicit y vs. 5-FUrd [%]</th><th>P</th><th>Cytotoxicit y vs. Control [%]</th><th>Cytotoxicit y vs. TMZ [%]</th><th>P</th><th>Cytotoxicit y vs. Control [%]</th><th>Cytotoxicit y vs. TMZ [%]</th><th>P</th><th>Cytotoxicit y vs. Control [%]</th><th>Cytotoxicit y vs. 5-FUrd [%]</th><th>Cytotoxicit y vs. TMZ [%]</th><th>P</th></tr>
<tr><td rowspan="6">εNL_5.cycl8.0.0 (10)</td><td>1.56</td><td rowspan="6">=</td><td rowspan="6"><</td><td rowspan="6"></td><td rowspan="4">=</td><td rowspan="6"><</td><td rowspan="6"></td><td rowspan="6"></td><td rowspan="6"></td><td rowspan="6"></td><td rowspan="6"></td><td rowspan="6"></td><td rowspan="6"></td><td rowspan="6">=</td><td rowspan="6">=</td><td rowspan="6">=</td><td rowspan="6"></td></tr>
<tr><td>3.12</td></tr>
<tr><td>6.25</td></tr>
<tr><td>12.5</td></tr>
<tr><td>25</td><td>21.7</td></tr>
<tr><td>50</td><td>16.9</td></tr>
</table>
